# A Combined Network Pharmacology and Molecular Docking Approach to Investigate Candidate Active Components and Multitarget Mechanisms of Hemerocallis Flowers on Antidepressant Effect

**DOI:** 10.1155/2021/7127129

**Published:** 2021-06-30

**Authors:** Tiancheng Ma, Yu Sun, Chang Jiang, Weilin Xiong, Tingxu Yan, Bo Wu, Ying Jia

**Affiliations:** ^1^School of Traditional Chinese Materia Medica, Shenyang Pharmaceutical University, Wenhua Road 103, Shenyang 110016, China; ^2^Research Institute of Medicine and Pharmacy, Qiqihar Medical University, Bukui North Street 333, Qiqihar 161006, China; ^3^School of Functional Food and Wine, Shenyang Pharmaceutical University, Wenhua Road 103, Shenyang 110016, China

## Abstract

**Objective:**

The purpose of our research is to systematically explore the multiple mechanisms of *Hemerocallis fulva* Flowers (HF) on depressive disorder (DD).

**Methods:**

The components of HF were searched from the literature. The targets of components were obtained from PharmMapper. After that, Cytoscape software was used to build a component-target network. The targets of DD were collected from DisGeNET, PharmGKB, TTD, and OMIM. Protein-protein interactions (PPIs) among the DD targets were executed to screen the key targets. Afterward, the GO and KEGG pathway enrichment analysis were performed by the KOBAS database. A compound-target-KEGG pathway network was built to analyze the key compounds and targets. Finally, the potential active substances and targets were validated by molecular docking.

**Results:**

A total of 55 active compounds in HF, 646 compound-related targets, and 527 DD-related targets were identified from public databases. After treated with PPI, 219 key targets of DD were acquired. The gene enrichment analysis suggested that HF probably benefits DD patients by modulating pathways related to the nervous system, endocrine system, amino acid metabolism, and signal transduction. The network analysis showed the critical components and targets of HF on DD. Results of molecular docking increased the reliability of this study.

**Conclusions:**

It predicted and verified the pharmacological and molecular mechanism of HF against DD from a holistic perspective, which will also lay a foundation for further experimental research and rational clinical application of DD.

## 1. Introduction

Depressive disorder (DD) is a severe and occasionally fatal mental disorder that occurs in 4.4% to 20% of the general population. It happens at any time peaking in older adulthood, and it is more prevalent in women than in men. According to the world health organization (WHO) report, depression will be the second most burdensome disease in terms of treatment and care costs by 2020 [1, 2]. Treatment for depression is necessary because depression can interfere with one's daily life, which can significantly affect relationships with family and friends. However, the specific pathophysiology basis of the development of depression remains unclear [3]. As a critical component of complementary and alternative medicine, Traditional Chinese Medicine (TCM) plays an essential role in treating depression.

TCM is a multicomponent, multitarget, and multipathway therapy, which can achieve its unique therapeutic effect by adjusting the biological network of the human body system. Therefore, it is difficult to detect the exact mechanism of TCM only through traditional experimental methods [4]. With the rapid development of bioinformatics, network pharmacology has become a new and efficient method to systematically reveal the molecular and pharmacological mechanisms of TCM [5]. Network pharmacology can reflect and elucidate the relationship among multiple components, multiple targets, and multiple diseases. At the same time, it abstracts the relationship into a network model and illustrates the action of drugs on the human biological network from a systematic perspective [6].

*Hemerocallis fulva* L. is a perennial herb of the Liliaceae family [7], which is indigenous to Asia, and its flowers are used as ornamental flowers, as well as for food and medicine. It was first recorded in the publication Supplement to Compendium of Materia Medica (Bencao Gangmu Shiyi). The root, seeding, and flower of *Hemerocallis fulva* L. are considered to have sweet, cool, and nontoxic properties and to be associated with the spleen, liver, and bladder meridians [8]. Previous investigations revealed its predominant chemical constituents including alkaloids [9], flavones [10], terpenes [11], steroidal saponins [12], and phenolic glycosides [13]. According to previous studies, *Hemerocallis fulva* Flowers (HF) have been used to treat various diseases including depression [14, 15], inflammation [16], insomnia [17], hepatosis [18], and cancer [19].

**Figure 1:**
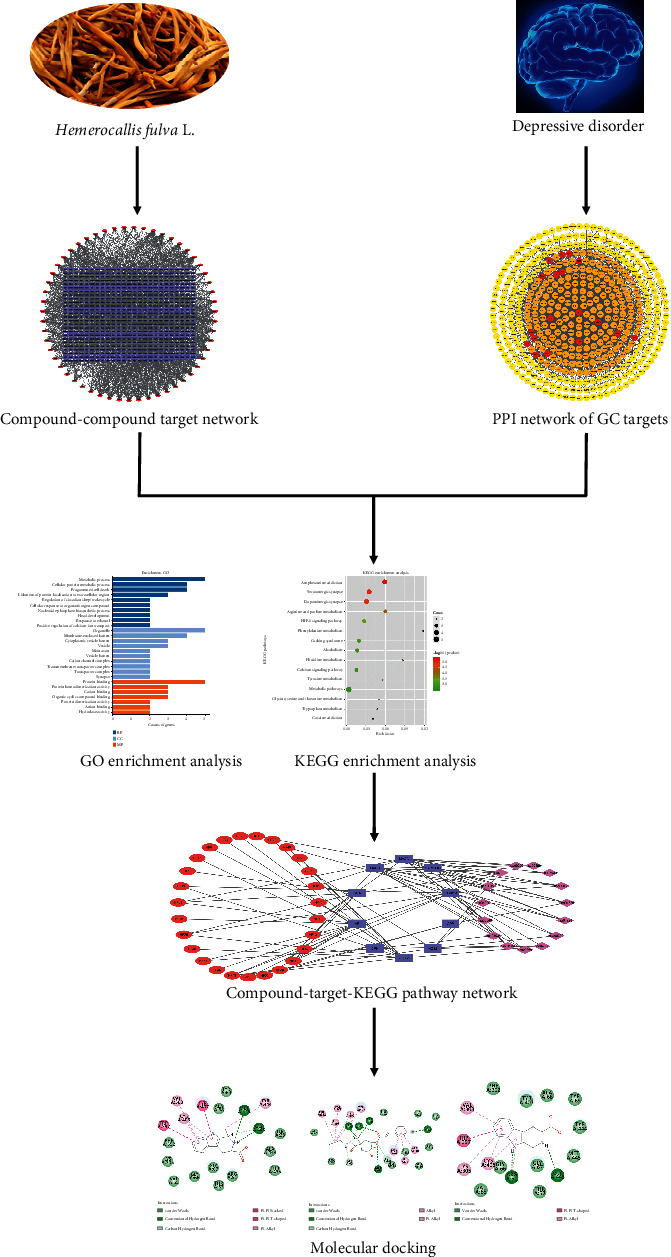
Workflow of the network pharmacology analysis of HF constituents against DD.

However, there were few research studies on the network pharmacology of HF, and the mechanism of HF in treating depression was not very clear. In this study, we proposed a network pharmacology method aiming at uncovering the active substances and mechanisms of HF in the treatment of DD. Firstly, the components from HF were searched from the literature. The components were filtered by the metrics of oral bioavailability and drug-likeness. The targets were predicted by PharmMapper. Secondly, protein-protein interactions (PPIs) among the targets associated with DD searched from the DisGeNET, PharmGKB, TTD, and OMIM databases were predicted by the STRING database, and the key targets of DD were acquired. Then, the intersection of the compound-compound target network and PPI network of DD targets was taken to find overlapping targets. After that, the GO and KEGG pathway enrichment analysis were performed by KOBAS. The compound-target-pathway (C-T-P) network was analyzed to obtain the key potential active substances and targets. Lastly, the key targets were further validated by molecular docking. The whole framework is shown in Figure 1.

Based on network pharmacology technology, this study aimed to explore the overall regulatory role of multicomponent, multitarget antidepressants in the molecular-level system of HF and to provide a theoretical basis for further experimental study and rational clinical application of HF.

## 2. Materials and Methods

### 2.1. Chemical Database Collection and Active Components Screening

To collect the compounds of HF, we searched the TCM Integrated Database [20] (TCMID, http://www.megabionet.org/tcmid/), which records a great deal of information about herbal ingredients, and the Traditional Chinese Medicine Systems Pharmacology Database [21] (TCMSP, http://lsp.nwu.edu.cn/), a unique systemic pharmacology platform for Chinese herbal medicine. Unfortunately, few compounds were collected, except for some simple amino acid compounds. So, we consulted a lot of studies [9, 10, 22–28] to collect the compounds of HF. The chemical structural formulas of HF compounds were drawn with ChemBioDraw Ultra 14.0.

Absorption, distribution, metabolism, and excretion (ADME) are used in drug discovery. Appropriate ADME screening can ensure that these candidate compounds have suitable pharmacokinetic properties. In this study, two ADME-related parameters were employed to screen out the active compounds in HF, including gastrointestinal (GI) absorption and drug-likeness evaluated by Lipinski's rule. GI absorption and drug-likeness were obtained from SwissADME (http://www.swissadme.ch/index.php) [29]. GI absorption is a pharmacokinetic behavior that is critical to evaluate the various stages of the drug discovery process. It can be calculated using an accurate prediction model, the Brain Or IntestinaL EstimateD permeation method (BOILED-Egg) [30]. Lipinski's rule of five is a rule of thumb to evaluate if a chemical compound with certain pharmacological or biological activities could be a likely orally active drug in humans [31, 32]. All screen-out compounds should follow Lipinski's rule, and the GI absorption value of compounds should be high.

### 2.2. Predicted Compound Targets for HF

The chemical structural formulas of HF active compounds were saved as “mol2.” formatted files and treated with the function of MM2 to optimize the 3D molecular structures by ChemBio3D Ultra 14.0. The 3D molecular structure files of HF were imported into PharmMapper [33] (http://lilab.ecust.edu.cn/pharmmapper/), which was an online server that utilized pharmacophore mapping to approach for potential drug target identification. In this study, the targets of each compound obtained from PharmMapper were selected as potential targets. The details about the selected targets are described in Table S1.

### 2.3. Depressive Disorder Targets

The genes associated with targets of DD were collected from DisGeNET [34] (http://www.disgenet.org/), PharmGKB [35] (https://www.pharmgkb.org/), TTD [36] (http://db.idrblab.net/ttd/), and OMIM (https://omim.org/). We searched these platforms with the keywords “depressive disorder,” “depression,” or “major depressive disorder.” Humans' protein targets were selected in this study. The details about the selected targets are described in Table S2.

### 2.4. Protein-Protein Interaction

STRING is a database, which can predict protein-protein interactions. The interactions include direct (physical) and indirect (functional) associations. They stem from computational prediction, from knowledge transfer between organisms, and from interactions aggregated from other (primary) databases. The data of protein-protein interaction (PPI) were obtained from the STRING database [37] (https://string-db.org/, ver. 11.0), with the species limited to “*Homo sapiens*.” PPIs with comprehensive scores >0.7 were reserved in this study.

### 2.5. Network Construction

Network construction was executed as follows: (1) compound-compound target network was structured by connecting active compounds and corresponding targets; (2) PPI network of DD targets was built by linking DD targets; (3) compound-DD target-KEGG pathway network was established by connecting compounds, overlapping targets between compound targets and core DD targets, and top 15 KEGG pathways. In network interactions, nodes represent compounds, targets, and KEGG pathways, while edges represent the interaction of each other. The network visualization software Cytoscape [38] (http://cytoscape.org/, ver. 3.7.2) was used to show all the above networks. The software is well suited for visualizing molecular interactions in networks. Besides, the tool of NetworkAnalyzer [39] provides a powerful set of data integration, analysis, and visualization capabilities for analyzing complex networks. For each node in the interactive network, three metrics are calculated to evaluate its topology characteristics. “Degree” is defined as the number of edges of node *i*. “Node betweenness” represents the number of shortest paths between pairs of nodes passing through node *i*. “Closeness” is the reciprocal of the sum of distances between node *i* and the other nodes.

### 2.6. Gene Ontology and KEGG Pathway Enrichment

Gene Ontology (GO) is a bioinformatics project aimed to unify the characteristics of genes and genetic products in all species. Semantic lexical criteria that define and describe the function of genes and proteins can be updated for further study. GO has three classifications, including molecular function (MF), biological process (BP), and cell component (CC). MF describes the activity of gene products in molecular biology, such as catalytic or binding activity. BP is a multistep process composed of ordered molecular functions. CC refers to organelles or gene product groups and represents the role of gene products. Kyoto Encyclopedia of Genes and Genomes (KEGG) is a database resource for understanding high-level functions and utilities of the biological system, such as the cell, the organism, and the ecosystem, from molecular-level information, especially large-scale molecular datasets generated by genome sequencing and other high-throughput experimental technologies.

KOBAS is a widely used gene set enrichment (GSE) analysis tool [40]. The current version is KOBAS 3.0, which was released at the end of 2019, covering 5,945 species with incorporated knowledge. The enrichment module can accept the gene list or gene expression data as input and generate the enriched gene set, corresponding name, *p* value, enrichment probability, and enrichment score according to the results of various methods. In this study, KOBAS was applied to perform GO and KEGG pathway enrichment analysis. Enriched GO terms and pathways were defined as those *p* value <0.05. The *p*value was corrected using the method introduced by Benjamini and Hochberg [41]. It controlled the false discovery rate, which was the expected percentage of rejected assumptions. Therefore, this method is more effective than others. The horizontal bar of GO enrichment and bubble chart of KEGG pathway enrichment were plotted by using the bioinformatic tool (http://www.bioinformatics.com.cn/), which was a free online data analysis platform.

### 2.7. Molecular Docking Verification

Molecular docking plays an important role in rational drug design. It is often used to predict the binding sites and binding postures between candidate drugs and targets, as well as to estimate the binding affinity of molecules [42, 43]. Surflex-Dock is an accurate docking program based on a protomol that can be automatically generated or user-defined. In this study, the Surflex-Dock plug-in included in the Sybyl-X (version 2.0, TRIPOS Inc.) was used to perform molecular docking. The binding ability was evaluated using a scoring function analysis, and the higher docking score represented better binding ability. The files of protein molecular structures were obtained from the PDB database (https://www.rcsb.org/). For docking studies with Surflex-Dock, the ligand-binding site protomol was generated using the ligand from the PDB file. The visualization of intermolecular forces between the candidate compound and their potential target was performed on Discovery Studio 2020 program.

## 3. Results

### 3.1. Compounds in HF and Pharmacokinetic Evaluation

After consulting literature, 81 herbal compounds with structural information were gathered. After the GI absorption and drug-likeness process, some compounds that did not meet the ADME criteria were added back into the database because of their high bioactivity proved in previous studies [14, 44–52]. Eventually, 55 compounds were reserved, which meant that these compounds might be the active compounds of HF. The detailed information is shown in Table 1.

### 3.2. Compound-Compound Target Network Analysis

**Figure 2:**
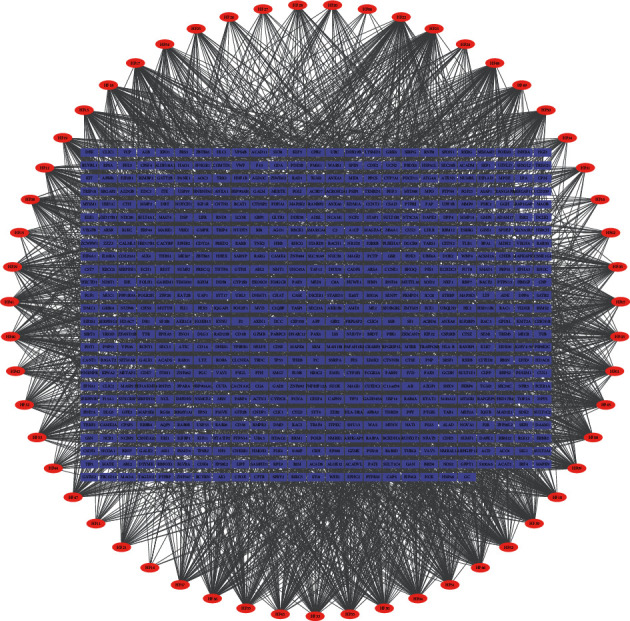
Compound-compound target network (red ellipses represent compounds contained in HF, blue rectangles represent compound targets).

The compound-compound target network is depicted in Figure 2, including 701 nodes (55 active compound nodes and 646 compound target nodes) and 3112 edges. In this network, the rectangle represented the target, and the oval represented the compound. We found that many targets were hit by multiple compounds; for example, vascular endothelial growth factor A (VEGFA) and albumin (ALB) were together modulated by multiple ingredients, including hesperidin and quercetin-3-O-*β*-D-glucopyranoside. The average number of targets per component is 56.6, and the mean degree of components per target is 4.8. It clearly showed that HF fit the multicomponent and multitarget characteristics of TCM. Consequently, we not only obtained an approximate observation of the relationship between bioactive compounds and compound targets, but also discovered the potential pharmacological effects of HF from this network.

### 3.3. Disease PPI Network Construction

**Figure 3:**
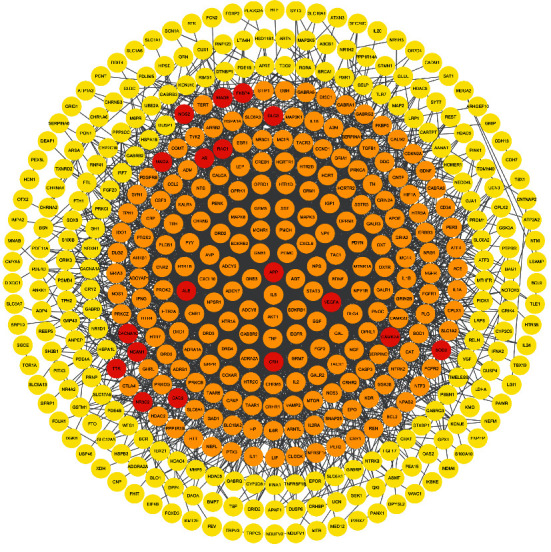
PPI network of DD targets (yellow circles represent nonkey targets of DD, orange circles represent key targets of DD, red circles represent overlapping targets between the compound target of HF and key targets of DD).

Based on the gene database DisGeNET, PharmGKB, TTD, and OMIM, there were a total of 527 candidate targets relevant to depressive disorder. After PPI was acquired, there were 446 nodes and 3905 edges in the DD target PPI network (Figure 3). The results of network analysis showed that there were 219 nodes whose degree value was higher than the median value, which meant that these targets were likely to be the key targets in the development of DD. The targets in the interior circle showed more interactions with targets than those in the exterior. After the intersection process, we found that there were 18 overlapping targets between compound targets of HF and key targets of DD. They are ALB, amyloid-beta precursor protein (APP), androgen receptor (AR), voltage-dependent L-type calcium channel subunit alpha-1C (CACNA1C), calcium-dependent protein kinase II alpha (CAMK2A), corticoliberin (CRH), discs large MAGUK scaffold protein 3 (DLG3), FK506 binding protein 4 (FKBP4), glutamate decarboxylase 2 (GAD2), monoamine oxidase A (MAOA), monoamine oxidase B (MAOB), neural cell adhesion molecule 1 (NCAM1), nitric oxide synthase 2 (NOS2), nuclear receptor subfamily 3 group C member 2 (NR3C2), Rac family small GTPase 1 (RAC1), superoxide dismutase 2 (SOD2), transthyretin (TTR), and VEGFA, which meant that these targets might be the key targets for HF treating DD.

### 3.4. Potential Synergistic Mechanisms Analysis of HF Target-DD Target Network

#### 3.4.1. GO Enrichment Analysis

**Figure 4:**
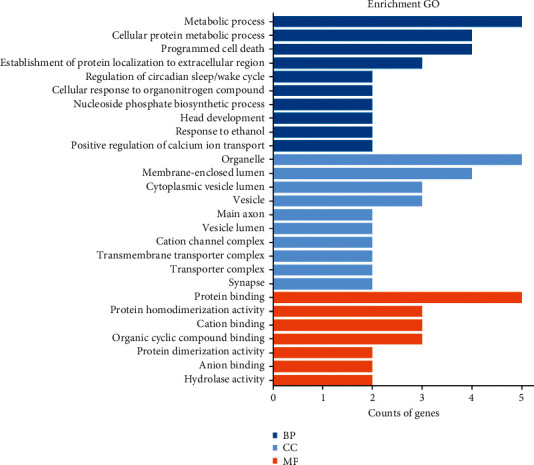
GO enrichment analysis for 18 overlapping targets.

After GO enrichment analysis of 18 overlapping targets, a total of 42 GO entries were found with the corrected *p*value <0.05.  Figure 4 lists the top10 entries of each category, namely, BP, CC, and MF. BP included regulation of circadian sleep/wake cycle (GO: 0042749), programmed cell death (GO: 0012501), head development (GO: 0060322), metabolic process (GO: 0008152), and positive regulation of calcium ion transport (GO: 0051928). CC included main axon (GO: 0044304), vesicle lumen (GO: 0031983), cation channel complex (GO: 0034703), synapse (GO: 0045202), and vesicle (GO: 0031982). MF included protein binding (GO: 0005515), cation binding (GO: 0043169), and hydrolase activity (GO: 0016787). The GO entries mentioned above were strongly associated with the central nervous system and mental diseases. This demonstrated that HF probably worked by engaging in the above biological processes, cellular components, and molecular functions.

#### 3.4.2. KEGG Pathway Analysis to Explore the Therapeutic Mechanisms of HF on DD

**Figure 5:**
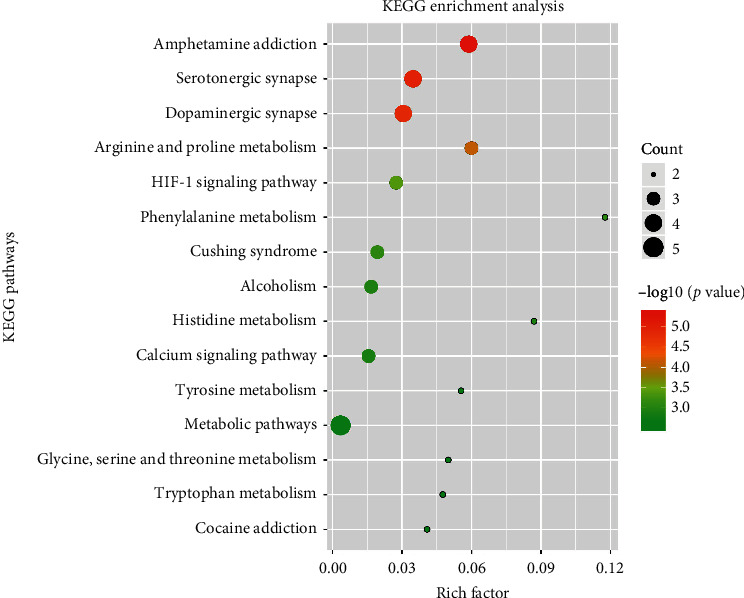
KEGG enrichment analysis for 18 overlapping targets.

The 18 overlapping targets were further mapped to 34 pathways with *p* < 0.05. The 34 pathways belonged to four categories: human diseases (9/34), organismal systems (13/34), environmental information processing (4/34), and metabolism (8/34). Thus, our findings showed that HF integrated multiple signaling pathways to the nervous system, endocrine system, amino acid metabolism, signal transduction, and substance dependence. Based on the results of pathway analysis, it was found that these high-degree pathways were closely related to the DD, such as serotonergic synapse and dopaminergic synapse. The top 15 KEGG pathways are shown in Figure 5.

#### 3.4.3. Compound-Target-KEGG Pathway Network Analysis

**Figure 6:**
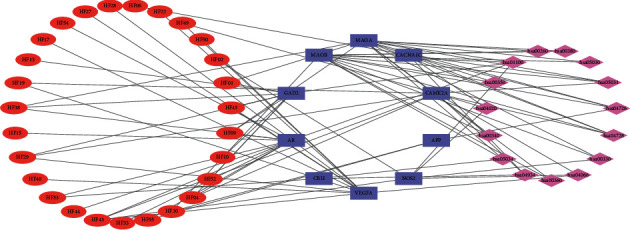
Compound-target-KEGG pathway network (red ellipses represent compounds contained in HF, blue rectangles represent compound targets, purple rhombuses represent KEGG pathway).

The details of KEGG pathways are described in Table 2, and the compound-target-pathway (C-T-P) network is shown in Figure 6, which contained 52 nodes including top 15 KEGG pathways, 10 targets related to top 15 KEGG pathways and 27 compounds, and 178 edges. The network was calculated to find the major nodes of compounds and targets. Finally, 12 compound nodes with their values of degree more than average were selected as crucial compound nodes, namely, naringin, (S)-2,4-dibutoxy-3-(hydroxymethyl)cyclopent-2-en-1-one, 5-O-p-coumaroylquinic acid butyl ester, hesperidin, lycoperodine-1, (-)-(1S,3S)-1-methyl-1,2,3,4-tetrahydro-*β*-carboline-3-carboxylic acid, hydroxydihydrobovolide, epigallocatechin gallate (EGCG), prunasin, quercetin-3-O-*β*-D-galactopyranoside, vitexin, and *α*-linolenic acid. It indicated that these compounds might play a more important role than others in the treatment of depressive disorder with HF. Five target nodes with values of degree, betweenness, and closeness more than average were selected as key target nodes, namely, MAOA, MAOB, AR, CAMK2A, and GAD2. It indicated that these targets may be crucial to the treatment of depressive disorder with HF.

### 3.5. Molecular Docking Results and Analysis

**Figure 7:**
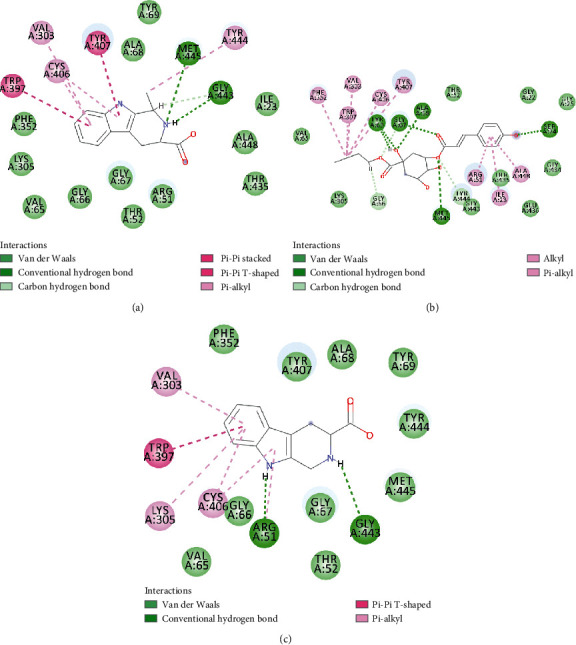
2D diagram of docking between compounds of HF and MAOA. (a) (-)-(1S,3S)-1-Methyl-1,2,3,4-tetrahydro-*β*-carboline-3-carboxylic acid. (b) 5-O-p-Coumaroylquinic acid butyl ester. (c) Lycoperodine-1.

To verify the top 5 key targets and their interacting compounds, the molecular docking simulation was carried out by the Surflex-Dock method. As the result in Table 3illustrated, most compounds had strong interactions with their targets. The average docking scores of compounds were more than 6. The results confirmed that most of these potential active compounds had strong binding affinities with the key targets, and the present network pharmacology method was reasonable. We take lycoperodine-1, (-)-(1s,3s)-1-methyl-1,2,3,4-tetrahydro-*β*-carboline-3-carboxylic acid, and 5-O-p-coumaroylquinic acid butyl ester as examples to show the visualization of intermolecular forces between compounds and MAOA target, which are displayed in Figure 7.

## 4. Discussion

Researchers have conducted a large number of studies to explore the pathogenesis of depression, but its exact pathogenesis is still unclear. The supposed pathogenesis of depression is the epigenetic hypothesis, neurotransmitter hypothesis, neurotrophic regeneration hypothesis, neurokinin hypothesis, neuroendocrine dysfunction hypothesis, and immune system abnormality hypothesis [53]. The research strategy of network pharmacology provides a unique and innovative pathway to study the mechanism of action of multicomponent and multitarget. HF is not only a nutrient food but also a drug with antidepressant activity. To elucidate the beneficial effects of HF on DD, the putative active ingredients and underlying mechanisms were comprehensively investigated using network pharmacology.

In the present study, a total of 55 active compounds in HF were screened by pharmacokinetic analysis, 646 compound-related targets were predicted by PharmMapper, and 527 DD-related targets were identified from public databases. After treated with PPI, 219 key targets in the development of DD were acquired. Among these targets, 18 targets were shared between compound-related and DD-related targets. Most entries from GO enrichment play important parts in the central nervous system, which affect different steps of synthesis or transportation of neurotransmitters. The KEGG pathway analysis proved that bioactive compounds from HF exerted a synergistic effect on the treatment of DD through numerous pathways and brain amino acid metabolism, such as amphetamine addiction, serotonergic synapse, dopaminergic synapse, arginine and proline metabolism, HIF-1 signaling pathway, phenylalanine metabolism, alcoholism, calcium signaling pathway, tyrosine metabolism, and tryptophan metabolism.

Furthermore, the top 5 key targets and top 12 key compounds were screened from the C-T-P network. These five screening targets have been shown to be highly correlated with depression. Monoamine oxidases (MAOs) play a crucial role during the development of various mental diseases [8]. There are two MAO isozymes, MAOA and MAOB. They are flavoenzymes, which bind to the outer mitochondrial membrane and catalyze the oxidative transformations of neurotransmitters like serotonin, norepinephrine, and dopamine [54]. However, deficiency of serotonin can lead to depression, so MAOA and MAOB were two important targets for DD. Hung et al. [55] found that loss of AR accelerated the development of depressive-like behaviors in mice under chronic mild stress (CMS), and mice with low androgen were more prone to depression-like behaviors [56]. The gene of CAMK2A has been reported to be associated with depression, and the expression of CAMK2A was significantly elevated in the depression tissues by 29% [57]. The role of CAMK2A as a signaling molecule inside neurons could influence the function of the brain in learning and memory. The translation regulation of the protein encoded by the gene could prove the long-term regulation of potentiation and depression [58]. Glutamate decarboxylase (GAD) is a rate-limiting enzyme for the conversion of glutamic acid to gamma-aminobutyric acid (GABA). GAD2 is an important enzyme that regulates depression-related neurotransmitters, which shows enhanced availability in situations of stress, responding to short-term demands for GABA [59]. GABAergic dysfunction in schizophrenia and mood disorders was reflected by decreased levels of GAD2 [60]. Therefore, HF may improve depression symptoms by regulating including but not limited to MAOA, MAOB, AR, CAMK2A, and GAD2.

Meanwhile, some of the screened compounds had been shown to antidepressant activity. It reported that naringin was a neuroactive flavonoid. It possessed functional beneficial neurobehavioral effects including anxiolytic, antidepressant, and memory enhancing [46]. Hesperidin, a well-known flavanone glycoside mostly found in citrus fruits, showed neuroprotective and antidepressant activity [61]. EGCG could attenuate the depressive status of mice, and the underlying mechanism may be related to the reduction of serum cortisol (CORT) and adrenocorticotropic hormone (ACTH), downregulation of malonyldialdehyde (MDA), interleukin-1 beta (IL-1*β*), interleukin 6 (IL-6), indoleamine 2,3-dioxygenase (IDO), and upregulation of superoxide dismutase (SOD) and glutathione peroxidase (GSH-PX) in the hippocampus [62]. Quercetin-3-O-*β*-D-galactopyranoside may improve depression-like effects by regulating the hypothalamic-pituitary-adrenal (HPA) axis and reducing the level of oxidative stress in the hippocampus [63]. Can et al. found that the antidepressant-like effect of vitexin was mediated through an increase in catecholamine levels in the synaptic cleft [64]. In a study of 150 elderly males from Crete, omega-3 *α*-linolenic acid adipose tissue stores were negatively correlated with depression [65]. The antidepressant-like effect of *Hemerocallis* extract was mainly related to flavonoids [14], which were similar to our findings. Our study suggested that, along with these reported compounds, some other putative active ingredients in HF might also possess antidepressant-like effects with diverse underlying mechanisms. Besides that, we also found some potential antidepressant compounds that have not been reported in the existing literature. They were (-)-(1S,3S)-1-methyl-1,2,3,4-tetrahydro-*β*-carboline-3-carboxylic acid, (S)-2,4-dibutoxy-3-(hydroxymethyl)cyclopent-2-en-1-one, 5-O-p-coumaroylquinic acid butyl ester, lycoperodine-1, hydroxydihydrobovolide, and prunasin. Subsequent experiments could verify their activity.

Molecular docking experiment is a new method that uses computer simulation of compound structure and related disease targets to execute molecular docking, calculate and analyze the bioactivity of the compound, and screen the material basis of pharmacodynamics. This method could quickly and efficiently discover some new bioactive lead compounds from the database. In this study, molecular docking results showed that targets predicted by PharmMapper were reasonable. Most of the screened compounds and their corresponding targets scored well. The structures of lycoperodine-1 and (-)-(1S,3S)-1-methyl-1,2,3,4-tetrahydro-*β*-carboline-3-carboxylic acid are similar to that of harmine, which was a carboline alkaloid and also was a MAOA inhibitor [66]. The similar scores of these compounds are also due to their structural similarity, which can prove the reliability of the molecular docking results.

## 5. Conclusion

In summary, the present study is the first one that combines active components, target prediction, network analysis, and gene enrichment analysis by a network pharmacology method to elucidate the molecular and pharmacological mechanism of HF against DD from a systematic perspective. In this research, we showed multiple targets of HF against DD for the first time. Based on this neural network model, active components, targets, and pathways in depressive disorder were initially explored to provide a preliminary theoretical basis for the design of subsequently targeted drugs. The results of molecular docking confirmed that the present network pharmacology method was reasonable. Nonetheless, more experiments should be implemented to verify the validity of our findings in further pharmacological and molecular research. Moreover, we hope that our study will be useful for antidepression drug discovery.

## Figures and Tables

**Table 1:** The information of active components in *Hemerocallis fulva* flowers (HF).

ID	Name	Molecular formula	Molecular weight	Structure	GI absorption	Conform to rules of Lipinski
HF01	(-)-(1S,3S)-1-Methyl-1, 2, 3, 4-tetrahydro-*β*-carboline-3-carboxylic acid	C_13_H_14_N_2_O_2_	230.3	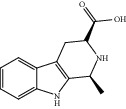	High	Yes

HF02	(+)-Dehydrovomifoliol	C_13_H_18_O_3_	222.3	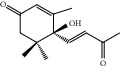	High	Yes

HF03	(3S,5 R)-Butyl-3-hydroxy-2-oxopyrrolidine-5-carboxylate	C_9_H_15_NO_4_	201.2	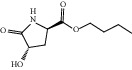	High	Yes

HF04	(S)-2,4-Dibutoxy-3-(hydroxymethyl) cyclopent-2-en-1-one	C_14_H_24_O_4_	256.3	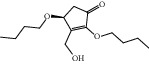	High	Yes

HF05	(S)-Abscisic acid	C_15_H_20_O_4_	264.3	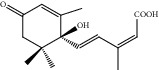	High	Yes

HF06	2′-Deoxyadenosine	C_10_H_13_N_5_O_3_	251.1	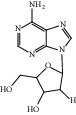	High	Yes

HF07	5, 6-Epoxy-3-hydroxy-7-megastigmen-9-ene	C_13_H_20_O_3_	224.3	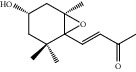	High	Yes

HF08	5-O-p-Coumaroyl-1,5-quinide lactone	C_16_H_16_O_7_	320.3	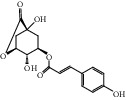	High	Yes

HF09	5-O-p-Coumaroylquinic acid butyl ester	C_20_H_26_O_8_	394.4	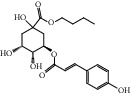	High	Yes

HF10	5-O-p-Coumaroylquinic acid methyl ester	C_17_H_20_O_8_	352.3	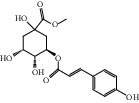	High	Yes

HF11	Apigenin	C_15_H_10_O_5_	270.3	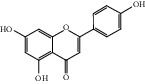	High	Yes

HF12	Catechin	C_15_H_14_O_6_	290.1	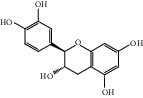	High	Yes

HF13	Chlorogenic acid	C_16_H_18_O_9_	354.3	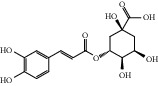	Low	No

HF14	Chrysin	C_15_H_10_O_4_	254.3	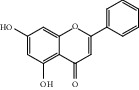	High	Yes

HF15	Daidzein	C_15_H_10_O_4_	254.3	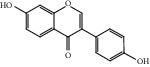	High	Yes

HF16	Dehydrololiolide	C_11_H_14_O_3_	194.2	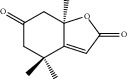	High	Yes

HF17	Ellagic acid	C_14_H_6_O_8_	302.2	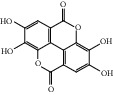	High	Yes

HF18	(-)-Epicatechin	C_15_H_14_O_6_	290.1	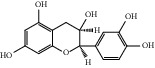	High	Yes

HF19	Epigallocatechin gallate	C_22_H_18_O_11_	458.4	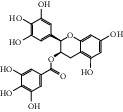	Low	No

HF20	Fulvanine D	C_10_H_15_NO_5_	229.2	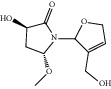	High	Yes

HF21	Galangin	C_15_H_10_O_5_	270.3	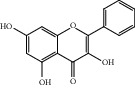	High	Yes

HF22	Hemerocallisamine I	C_13_H_18_N_2_O_6_	298.3	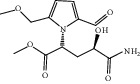	High	Yes

HF23	Hemerocallisamine II	C_10_H_15_NO_2_	181.2	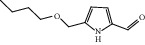	High	Yes

HF24	Hemerocallisamine III	C_10_H_15_NO_4_	213.2	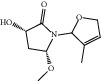	High	Yes

HF25	Hemerocallisamine IV	C_10_H_15_NO_4_	213.2	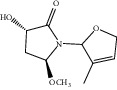	High	Yes

HF26	Hemerocallisamine V	C_9_H_13_NO_4_	199.2	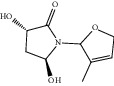	High	Yes

HF27	Hemerocallisamine VI	C_9_H_11_NO_4_	197.2	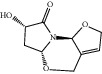	High	Yes

HF28	Hemerocallisamine VII	C_11_H_17_NO_5_	243.2	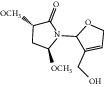	High	Yes

HF29	Hesperidin	C_28_H_34_O_15_	610.6	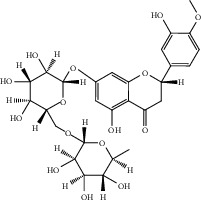	Low	No

HF30	Hydroxydihydrobovolide	C_11_H_18_O_3_	198.3	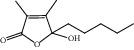	High	Yes

HF31	Isololiolide	C_11_H_16_O_3_	196.3	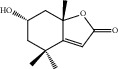	High	Yes

HF32	Kaempherol	C_15_H_10_O_6_	286.25	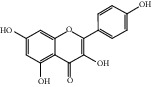	High	Yes

HF33	Linoleic acid	C_18_H_32_O_2_	280.5	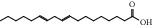	High	No

HF34	Loliolide	C_11_H_16_O_3_	196.3	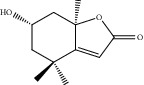	High	Yes

HF35	Longitubanine A	C_10_H_16_N_2_O_5_	244.3	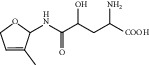	High	Yes

HF36	Longitubanine B	C_10_H_16_N_2_O_4_	228.3	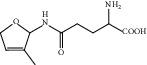	High	Yes

HF37	Luteolin	C_15_H_10_O_6_	286.0	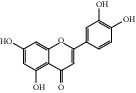	High	Yes

HF38	Lycoperodine-1	C_12_H_12_N_2_O_2_	216.2	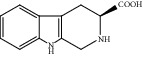	High	Yes

HF39	Methyl 2,4-dihydroxy-6-(4-hydroxyphenethyl)-3-methylbenzoate	C_17_H_18_O_5_	302.3	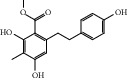	High	Yes

HF40	Morin	C_15_H_10_O_7_	302.3	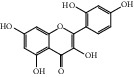	High	Yes

HF41	Myricetin	C_15_H_10_O_8_	318.3	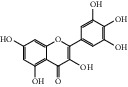	Low	No

HF42	Naringenin	C_15_H_12_O_5_	272.3	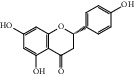	High	Yes

HF43	Naringin	C_27_H_32_O_14_	580.6	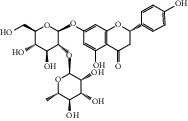	Low	No

HF44	Pinocembrin	C_15_H_12_O_4_	256.3	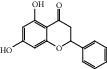	High	Yes

HF45	Prunasin	C_14_H_17_NO_6_	295.3	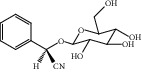	High	Yes

HF46	Pseudolaroside C	C_14_H_18_O_8_	314.3	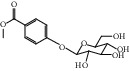	High	Yes

HF47	Quercetin	C_15_H_10_O_7_	302.3	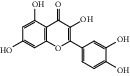	High	Yes

HF48	Quercetin-3-O-rutinoside	C_27_H_30_O_16_	610.5	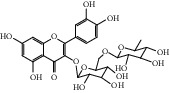	Low	No

HF49	Quercetin-3-O-*β*-D-galactopyranoside	C_21_H_20_O_12_	464.4	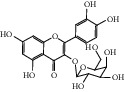	Low	No

HF50	Quercetin-3-O-*β*-D-glucopyranoside	C_21_H_20_O_12_	464.4	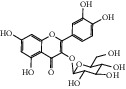	Low	No

HF51	Rosin	C_15_H_20_O_6_	296.3	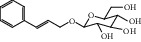	High	Yes

HF52	Salidroside	C_14_H_20_O_7_	300.3	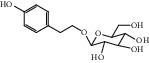	High	Yes

HF53	Vitexin	C_21_H_20_O_10_	432.4	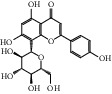	Low	No

HF54	Wogonin	C_16_H_12_O_5_	284.3	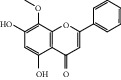	High	Yes

HF55	*α*-Linolenic acid	C_18_H_30_O_2_	278.4	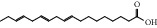	High	No

**Table 2:** Pathways associated with 18 overlapping targets according to enrichment analysis base on KEGG.

ID	Pathway	*p*value	Count	Gene IDs
hsa05031	Amphetamine addiction	7.39*E*-09	4	MAOB, MAOA, CACNA1C, CAMK2A
hsa04726	Serotonergic synapse	5.65*E*-08	4	MAOB, APP, CACNA1C, MAOA
hsa04728	Dopaminergic synapse	9.39*E*-08	4	MAOB, MAOA, CACNA1C, CAMK2A
hsa00330	Arginine and proline metabolism	6.34*E*-07	3	MAOB, NOS2, MAOA
hsa04066	HIF-1 signaling pathway	6.10*E*-06	3	NOS2, VEGFA, CAMK2A
hsa00360	Phenylalanine metabolism	1.64*E*-05	2	MAOB, MAOA
hsa04934	Cushing syndrome	1.71*E*-05	3	CRH, CACNA1C, CAMK2A
hsa05034	Alcoholism	2.65*E*-05	3	MAOB, CRH, MAOA
hsa00340	Histidine metabolism	2.88*E*-05	2	MAOB, MAOA
hsa04020	Calcium signaling pathway	3.25*E*-05	3	NOS2, CACNA1C, CAMK2A
hsa00350	Tyrosine metabolism	6.73*E*-05	2	MAOB, MAOA
hsa01100	Metabolic pathways	6.98*E*-05	5	MAOB, NOS2, AR, MAOA, GAD2
hsa00260	Glycine, serine, and threonine metabolism	8.23*E*-05	2	MAOB, MAOA
hsa00380	Tryptophan metabolism	9.04*E*-05	2	MAOB, MAOA
hsa05030	Cocaine addiction	1.22*E*-04	2	MAOB, MAOA

**Table 3:** Results of molecular docking of 5 key targets.

ID	Name	Target	PDB ID	Total score
HF01	(-)-(1s, 3s)-1-Methyl-1,2,3,4-tetrahydro-*β*-carboline-3-carboxylic acid	MAOA	2bxr	6.764
HF09	5-O-p-Coumaroylquinic acid butyl ester	MAOA	2bxr	9.395
HF38	Lycoperodine-1	MAOA	2bxr	6.035
HF30	Hydroxydihydrobovolide	MAOB	2bk3	6.579
HF04	(s)-2,4-Dibutoxy-3-(hydroxymethyl)cyclopent-2-en-1-one	AR	3b68	8.112
HF09	5-o-p-Coumaroylquinic acid butyl ester	AR	3b68	7.554
HF38	Lycoperodine-1	AR	3b68	4.619
HF43	Naringin	AR	3b68	-0.167
HF53	Vitexin	AR	3b68	4.508
HF55	*α*-Linolenic acid	AR	3b68	10.392
HF04	(s)-2,4-Dibutoxy-3-(hydroxymethyl)cyclopent-2-en-1-one	CAMK2A	2vz6	7.689
HF19	Epigallocatechin gallate	CAMK2A	2vz6	8.921
HF29	Hesperidin	CAMK2A	2vz6	7.035
HF43	Naringin	CAMK2A	2vz6	4.630
HF49	Quercetin-3-o-*β*-d-galactopyranoside	CAMK2A	2vz6	5.367
HF01	(-)-(1s,3s)-1-Methyl-1,2,3,4-tetrahydro-*β*-carboline-3-carboxylic acid	GAD2	2okk	5.580
HF04	(s)-2,4-Dibutoxy-3-(hydroxymethyl)cyclopent-2-en-1-one	GAD2	2okk	6.110
HF09	5-O-p-Coumaroylquinic acid butyl ester	GAD2	2okk	5.097
HF29	Hesperidin	GAD2	2okk	4.159
HF38	Lycoperodine-1	GAD2	2okk	4.555
HF45	Prunasin	GAD2	2okk	4.916

## Data Availability

The data used to support the findings of this study are available from the corresponding author upon request.

## Supplementary Materials

Supplementary Materials Table S1: the results of compound targets predicted by PharmMapper. Table S2: targets of depressive disorder obtained from the database.
