# Supplementary material for: A Combined Network Pharmacology and Molecular Docking Approach to Investigate Candidate Active Components and Multitarget Mechanisms of Hemerocallis Flowers on Antidepressant Effect
Source: Evid Based Complement Alternat Med. 2021 Jun 30;2021:7127129. doi: 10.1155/2021/7127129 (PMC8266453; doi:10.1155/2021/7127129)
Supplement: Supplementary Materials — Table S1: the results of compound targets predicted by PharmMapper. Table S2: targets of depressive disorder obtained from the database. [file 7127129.f1.pdf]

Table S1 The results of Compound target predicted by PharmMapper

No.	compound	PBD ID	Entry	Gene names
1	Naringin	2dgu	O60506	SYNCRIP
2	Naringin	1nu9	P00734	F2
3	Naringin	1x65	O75534	CSDE1
4	Naringin	2bc9	P32455	GBP1
5	Naringin	2ep8	O00541	PES1
6	Naringin	1zag	P25311	AZGP1
7	Naringin	2c9o	Q9Y265	RUVBL1
8	Naringin	1b4f	P29323	EPHB2
9	Naringin	2v9k	Q3MIT2	PUS10
10	Naringin	1d5b	P01834	IGKC
11	Naringin	2cpt	O75351	VPS4B
12	Naringin	3bkb	P07332	FES
13	Naringin	2jun	O15344	MID1
14	Naringin	1l9x	Q92820	GGH
15	Naringin	1w98	P24864	CCNE1
16	Naringin	2j91	P30566	ADSL
17	Naringin	1xap	P10826	RARB
18	Naringin	2e1o	Q03014	HHEX
19	Naringin	3epz	P26358	DNMT1
20	Naringin	1wi3	Q9UPW6	SATB2
21	Naringin	2a98	Q96DU7	ITPKC
22	Naringin	1inz	Q9Y6I3	EPN1
23	Naringin	2da7	O60315	ZEB2
24	Naringin	2pp4	Q06455	RUNX1T1
25	Naringin	1qib	P08253	MMP2
26	Naringin	2r55	Q9NSY2	STARD5
27	Naringin	1zvs	P61769	B2M
28	Naringin	2nzt	P52789	HK2
29	Naringin	2dlw	O60496	DOK2
30	Naringin	1u3r	Q92731	ESR2
31	Naringin	1bj1	P15692	VEGFA
32	Naringin	2ee4	Q13017	ARHGAP5
33	Naringin	2csh	O43298	ZBTB43
34	Naringin	2om5	Q02246	CNTN2
35	Naringin	3b68	P10275	AR
36	Naringin	3ifb	P12104	FABP2
37	Naringin	2o06	P19623	SRM
38	Naringin	1mje	P60896	SEM1
39	Naringin	3eay	Q9BQF6	SENP7
40	Naringin	2iwg	P19474	TRIM21
41	Naringin	2odq	P06681	C2
42	Naringin	3e7g	P35228	NOS2
43	Naringin	2djp	Q96S90	LYSMD1
44	Naringin	2ql6	Q9NWW6	NMRK1
45	Naringin	2pph	Q99759	MAP3K3
46	Naringin	2bsk	P62072	TIMM10
47	Naringin	2dll	Q15306	IRF4
48	Naringin	2i03	P27487	DPP4
49	Naringin	2frs	P29373	CRABP2
50	Naringin	2hzp	Q16719	KYNU
51	Naringin	2bu7	Q15119	PDK2
52	Naringin	1y8o	Q15120	PDK3

53	Naringin	2c0o	P08631	HCK
54	Naringin	2e8o	Q9Y3Z3	SAMHD1
55	Naringin	1igr	P08069	IGF1R
56	Naringin	2vz6	Q9UQM7	CAMK2A
57	Naringin	1k5g	P43487	RANBP1
58	Naringin	1spj	P06870	KLK1
59	Naringin	3fy2	P29320	EPHA3
60	Naringin	2yuc	Q9BUZ4	TRAF4
61	Naringin	2cls	Q92730	RND1
62	Naringin	1wl4	Q9BWD1	ACAT2
63	longitubanines A	1n8z	P04626	ERBB2
64	longitubanines A	2nn6	Q06265	EXOSC9
65	longitubanines A	3dpl	Q93034	CUL5
66	longitubanines A	2raj	Q9Y5X1	SNX9
67	longitubanines A	2q5e	O14595	CTDSP2
68	longitubanines A	1zbu	Q8IV48	ERI1
69	longitubanines A	3eg9	Q15436	SEC23A
70	longitubanines A	2ch9	O76096	CST7
71	longitubanines A	1z68	Q12884	FAP
72	longitubanines A	1jv1	Q16222	UAP1
73	longitubanines A	2hak	Q9P0L2	MARK1
74	longitubanines A	1ivh	P26440	IVD
75	longitubanines A	3f5n	Q99574	SERPINI1
76	longitubanines A	1lar	P10586	PTPRF
77	longitubanines A	1x66	Q01543	FLI1
78	longitubanines A	2d8n	P35243	RCVRN
79	longitubanines A	2h60	P51532	SMARCA4
80	longitubanines A	2jis	Q9Y600	CSAD
81	longitubanines A	2ann	P51513	NOVA1
82	longitubanines A	1xdt	Q99075	HBEGF
83	longitubanines A	2avd	Q86VU5	COMTD1
84	longitubanines A	1x6a	P53671	LIMK2
85	longitubanines A	2ast	P46527	CDKN1B
86	longitubanines A	2csh	O43298	ZBTB43
87	longitubanines A	1x5m	Q9HB71	CACYBP
88	longitubanines A	1rt9	P00491	PNP
89	longitubanines A	2da7	O60315	ZEB2
90	longitubanines A	2hye	Q16531	DDB1
91	longitubanines A	2h08	P60891	PRPS1
92	longitubanines A	2j91	P30566	ADSL
93	longitubanines A	1vyh	P68402	PAFAH1B2
94	longitubanines A	3bhy	O43293	DAPK3
95	longitubanines A	1egw	Q02078	MEF2A
96	longitubanines A	2e29	Q9BQ39	DDX50
97	longitubanines A	2ija	P18440	NAT1
98	longitubanines A	1pkx	P31939	ATIC
99	longitubanines A	2csw	O76064	RNF8
100	longitubanines A	2pzg	P13569	CFTR
101	longitubanines A	2aff	P46013	MKI67
102	longitubanines A	1w9c	O14980	XPO1
103	longitubanines A	2bc9	P32455	GBP1
104	longitubanines A	2wgh	P23921	RRM1
105	longitubanines A	2f3i	P52434	POLR2H
106	longitubanines A	3bsz	P02766	TTR

107	longitubanines A	2p39	Q9GZV9	FGF23
108	longitubanines A	2coz	Q5VT06	CEP350
109	longitubanines A	3bky	P11836	MS4A1
110	longitubanines A	3enm	P52564	MAP2K6
111	longitubanines A	2nmp	P32929	CTH
112	longitubanines A	3bg9	P11498	PC
113	longitubanines A	1cf4	Q07912	TNK2
114	longitubanines A	2coa	Q9BZL6	PRKD2
115	longitubanines A	2cmw	Q9HCP0	CSNK1G1
116	longitubanines A	1jrh	P15260	IFNGR1
117	longitubanines A	2ad1	O75897	SULT1C4
118	longitubanines A	2zfh	O60888	CUTA
119	longitubanines A	1w45	P13928	ANXA8
120	longitubanines A	2e6o	O60381	HBP1
121	longitubanines A	3bg1	P55735	SEC13
122	longitubanines A	3g1n	Q7Z6Z7	HUWE1
123	longitubanines A	2aex	P36551	CPOX
124	longitubanines A	2iwg	P19474	TRIM21
125	longitubanines A	2cke	Q9UIK4	DAPK2
126	longitubanines A	2r37	P22352	GPX3
127	longitubanines A	2uzg	Q8TEY7	USP33
128	longitubanines A	1pve	P54727	RAD23B
129	longitubanines A	2ahx	Q15303	ERBB4
130	longitubanines A	1lfg	P02788	LTF
131	longitubanines A	3dkm	Q9ULT8	HECTD1
132	longitubanines A	1us1	Q16853	AOC3
133	longitubanines B	1ujv	Q86UL8	MAGI2
134	longitubanines B	2nn6	Q06265	EXOSC9
135	longitubanines B	2p0c	Q12866	MERTK
136	longitubanines B	2pxx	P0DPD7	EEF1AKMT4
137	longitubanines B	1xjv	Q9NUX5	POT1
138	longitubanines B	1zbu	Q8IV48	ERI1
139	longitubanines B	1fqv	Q13309	SKP2
140	longitubanines B	1egw	Q02078	MEF2A
141	longitubanines B	2yt4	Q8WYQ5	DGCR8
142	longitubanines B	3eh2	P53992	SEC24C
143	longitubanines B	1ln3	Q9UKL6	PCTP
144	longitubanines B	3fe1	P17066	HSPA6
145	longitubanines B	1jpk	P06132	UROD
146	longitubanines B	1k78	Q02548	PAX5
147	longitubanines B	3bsq	P49862	KLK7
148	longitubanines B	1lar	P10586	PTPRF
149	longitubanines B	2ann	P51513	NOVA1
150	longitubanines B	1fxy	P00742	F10
151	longitubanines B	2e44	O00425	IGF2BP3
152	longitubanines B	1d6v	P01834	IGKC
153	longitubanines B	2raj	Q9Y5X1	SNX9
154	longitubanines B	1qo5	P05062	ALDOB
155	longitubanines B	2dkp	Q9HAU0	PLEKHA5
156	longitubanines B	2h32	P12018	VPREB1
157	longitubanines B	3dlj	Q96KN2	CNDP1
158	longitubanines B	1nu9	P00734	F2
159	longitubanines B	1z0j	Q9H1K0	RBSN
160	longitubanines B	1nuf	Q08188	TGM3

161	longitubanines B	1x5m	Q9HB71	CACYBP
162	longitubanines B	3bhy	O43293	DAPK3
163	longitubanines B	2v62	Q86Y07	VRK2
164	longitubanines B	2da7	O60315	ZEB2
165	longitubanines B	1olz	Q92854	SEMA4D
166	longitubanines B	2csh	O43298	ZBTB43
167	longitubanines B	2bc9	P32455	GBP1
168	longitubanines B	1ow1	Q96T58	SPEN
169	longitubanines B	3bg9	P11498	PC
170	longitubanines B	2ad1	O75897	SULT1C4
171	longitubanines B	2ys5	O43559	FRS3
172	longitubanines B	2j7q	P0CG48	UBC
173	longitubanines B	2f8n	O75367	MACROH2A1
174	longitubanines B	1wuu	P51570	GALK1
175	longitubanines B	2dmz	Q8NI35	PATJ
176	longitubanines B	1fg9	P15260	IFNGR1
177	longitubanines B	1rgo	P47974	ZFP36L2
178	longitubanines B	2j91	P30566	ADSL
179	longitubanines B	2ysd	Q96QZ7	MAGI1
180	longitubanines B	1w98	P24864	CCNE1
181	longitubanines B	2p39	Q9GZV9	FGF23
182	longitubanines B	1fe8	P04275	VWF
183	longitubanines B	2fxr	P20231	TPSB2
184	longitubanines B	2cpt	O75351	VPS4B
185	longitubanines B	2ak4	P01889	HLA-B
186	longitubanines B	2w96	P11802	CDK4
187	longitubanines B	1fsu	P15848	ARSB
188	longitubanines B	2cmw	Q9HCP0	CSNK1G1
189	longitubanines B	3e0l	Q9Y2T3	GDA
190	longitubanines B	1xdt	Q99075	HBEGF
191	longitubanines B	3enm	P52564	MAP2K6
192	longitubanines B	2ep8	O00541	PES1
193	longitubanines B	2rli	O43819	SCO2
194	longitubanines B	1jv1	Q16222	UAP1
195	longitubanines B	2d9p	Q9H361	PABPC3
196	longitubanines B	3g1n	Q7Z6Z7	HUWE1
197	longitubanines B	2h8r	P35680	HNF1B
198	longitubanines B	2eb1	Q9UPY3	DICER1
199	longitubanines B	1l9x	Q92820	GGH
200	longitubanines B	2arp	P08476	INHBA
201	longitubanines B	2eje	P78347	GTF2I
202	longitubanines B	2ejr	O60341	KDM1A
203	longitubanines B	2d9w	O60496	DOK2
204	longitubanines B	2coa	Q9BZL6	PRKD2
205	longitubanines B	1ntg	P54577	YARS1
206	longitubanines B	2c0o	P08631	HCK
207	Luteolin	2de0	Q9BYC5	FUT8
208	Luteolin	3c4m	P01270	PTH
209	Luteolin	3fmo	Q9UMR2	DDX19B
210	Luteolin	2fy1	P0DJD3	RBMY1A1
211	Luteolin	2dky	Q96QB1	DLC1
212	Luteolin	2dba	Q9H3U1	UNC45A
213	Luteolin	3bo5	Q53H47	SETMAR
214	Luteolin	1x66	Q01543	FLI1

215	Luteolin	1d6v	P01834	IGKC
216	Luteolin	2kg4	P24522	GADD45A
217	Luteolin	1kgd	O14936	CASK
218	Luteolin	1x86	Q9NZN5	ARHGEF12
219	Luteolin	2coa	Q9BZL6	PRKD2
220	Luteolin	2cra	Q92826	HOXB13
221	Luteolin	2ppi	Q9H2C0	GAN
222	Luteolin	2csw	O76064	RNF8
223	Luteolin	1itv	P14780	MMP9
224	Luteolin	1wi5	Q14690	PDCD11
225	Luteolin	7ics	P06746	POLB
226	Luteolin	2cmw	Q9HCP0	CSNK1G1
227	Luteolin	1wry	O75368	SH3BGRL
228	Luteolin	2fy4	P28329	CHAT
229	Luteolin	1vyh	P68402	PAFAH1B2
230	Luteolin	2j7q	P0CG48	UBC
231	Luteolin	2vcy	Q9BV79	MECR
232	Luteolin	2ys5	O43559	FRS3
233	Luteolin	2enk	Q6PML9	SLC30A9
234	Luteolin	2dnk	Q9BZC1	CELF4
235	Luteolin	1lo1	O95718	ESRRB
236	Luteolin	2cpt	O75351	VPS4B
237	Luteolin	2bxg	P02768	ALB
238	Luteolin	2ep8	O00541	PES1
239	Luteolin	3enm	P52564	MAP2K6
240	Luteolin	1x7z	P12694	BCKDHA
241	Luteolin	2pom	Q15750	TAB1
242	Luteolin	1fe8	P04275	VWF
243	Luteolin	2nn6	Q06265	EXOSC9
244	Luteolin	1w6j	P48449	LSS
245	Luteolin	1w60	P12004	PCNA
246	Luteolin	2c9o	Q9Y265	RUVBL1
247	Luteolin	2w96	P11802	CDK4
248	Luteolin	3ch4	Q15126	PMVK
249	Luteolin	1uf0	O15075	DCLK1
250	Luteolin	2csh	O43298	ZBTB43
251	Luteolin	2ast	P46527	CDKN1B
252	Luteolin	1inz	Q9Y6I3	EPN1
253	Luteolin	2aa7	P08235	NR3C2
254	Luteolin	2d86	Q9UKW4	VAV3
255	Luteolin	1imh	O94916	NFAT5
256	Luteolin	1rgo	P47974	ZFP36L2
257	Chrysin	1x86	Q9NZN5	ARHGEF12
258	Chrysin	2cra	Q92826	HOXB13
259	Chrysin	2ppi	Q9H2C0	GAN
260	Chrysin	2enk	Q6PML9	SLC30A9
261	Chrysin	2coa	Q9BZL6	PRKD2
262	Chrysin	1d5b	P01834	IGKC
263	Chrysin	2vcy	Q9BV79	MECR
264	Chrysin	1fe8	P04275	VWF
265	Chrysin	2ys5	O43559	FRS3
266	Chrysin	2cpt	O75351	VPS4B
267	Galangin	2cop	Q9BR61	ACBD6
268	Galangin	2eon	Q9Y2L8	ZKSCAN5

269	Galangin	2dkx	O95238	SPDEF
270	Galangin	3fmo	Q9UMR2	DDX19B
271	Galangin	2h63	P53004	BLVRA
272	Galangin	1z68	Q12884	FAP
273	Galangin	2ahx	Q15303	ERBB4
274	Galangin	2cra	Q92826	HOXB13
275	Galangin	1x86	Q9NZN5	ARHGEF12
276	Galangin	2coa	Q9BZL6	PRKD2
277	Galangin	2nn6	Q06265	EXOSC9
278	Galangin	2ppi	Q9H2C0	GAN
279	Galangin	2a2d	Q01415	GALK2
280	Galangin	2v62	Q86Y07	VRK2
281	Galangin	1kv3	P21980	TGM2
282	Galangin	1vyh	P68402	PAFAH1B2
283	Galangin	2coo	P11182	DBT
284	Galangin	1wji	Q9H7E2	TDRD3
285	Galangin	2enj	Q04759	PRKCQ
286	Galangin	3dpl	Q93034	CUL5
287	Galangin	1d5b	P01834	IGKC
288	Galangin	1wi5	Q14690	PDCD11
289	Galangin	2f3i	P52434	POLR2H
290	Galangin	2ejr	O60341	KDM1A
291	Galangin	2aff	P46013	MKI67
292	Galangin	2ys5	O43559	FRS3
293	Galangin	3eh2	P53992	SEC24C
294	Galangin	2pom	Q15750	TAB1
295	Galangin	1x7z	P12694	BCKDHA
296	Galangin	2w96	P11802	CDK4
297	Galangin	2vcy	Q9BV79	MECR
298	Galangin	2cpt	O75351	VPS4B
299	Galangin	2jrj	Q96PM5	RCHY1
300	Galangin	1fe8	P04275	VWF
301	Galangin	1wry	O75368	SH3BGRL
302	Galangin	3ifb	P12104	FABP2
303	Galangin	1onq	P06126	CD1A
304	Galangin	2kbq	Q9Y6N9	USH1C
305	Galangin	1h2t	P52298	NCBP2
306	Galangin	1fsu	P15848	ARSB
307	Galangin	2hak	Q9P0L2	MARK1
308	Apigenin	2de0	Q9BYC5	FUT8
309	Apigenin	3fmo	Q9UMR2	DDX19B
310	Apigenin	1x86	Q9NZN5	ARHGEF12
311	Apigenin	2ppi	Q9H2C0	GAN
312	Apigenin	2cra	Q92826	HOXB13
313	Apigenin	1kgd	O14936	CASK
314	Apigenin	1d5b	P01834	IGKC
315	Apigenin	2coa	Q9BZL6	PRKD2
316	Apigenin	2enk	Q6PML9	SLC30A9
317	Apigenin	7ics	P06746	POLB
318	Apigenin	2j7q	P0CG48	UBC
319	Apigenin	1x7z	P12694	BCKDHA
320	Apigenin	2ys5	O43559	FRS3
321	Apigenin	2fy4	P28329	CHAT
322	Apigenin	2vcy	Q9BV79	MECR

323	Apigenin	2bxg	P02768	ALB
324	Apigenin	2cpt	O75351	VPS4B
325	Apigenin	1fe8	P04275	VWF
326	Apigenin	2i6a	P55263	ADK
327	Apigenin	2c9o	Q9Y265	RUVBL1
328	Apigenin	2pom	Q15750	TAB1
329	Apigenin	1s9i	P36507	MAP2K2
330	Apigenin	2w96	P11802	CDK4
331	Apigenin	1inz	Q9Y6I3	EPN1
332	Quercetin	3fer	O75369	FLNB
333	Quercetin	2de0	Q9BYC5	FUT8
334	Quercetin	3fmo	Q9UMR2	DDX19B
335	Quercetin	1wm5	P19878	NCF2
336	Quercetin	3cki	P78536	ADAM17
337	Quercetin	2h63	P53004	BLVRA
338	Quercetin	3bo5	Q53H47	SETMAR
339	Quercetin	1d6v	P01834	IGKC
340	Quercetin	2ahx	Q15303	ERBB4
341	Quercetin	2c9o	Q9Y265	RUVBL1
342	Quercetin	2coa	Q9BZL6	PRKD2
343	Quercetin	1x86	Q9NZN5	ARHGEF12
344	Quercetin	1kgd	O14936	CASK
345	Quercetin	2ppi	Q9H2C0	GAN
346	Quercetin	3bg1	P55735	SEC13
347	Quercetin	1szb	O00187	MASP2
348	Quercetin	2ad1	O75897	SULT1C4
349	Quercetin	1wi5	Q14690	PDCD11
350	Quercetin	2coo	P11182	DBT
351	Quercetin	1d5b	P01834	IGKC
352	Quercetin	7ics	P06746	POLB
353	Quercetin	2csw	O76064	RNF8
354	Quercetin	1vyh	P68402	PAFAH1B2
355	Quercetin	1wry	O75368	SH3BGRL
356	Quercetin	2enk	Q6PML9	SLC30A9
357	Quercetin	2fy4	P28329	CHAT
358	Quercetin	2dnk	Q9BZC1	CELF4
359	Quercetin	3enm	P52564	MAP2K6
360	Quercetin	2j7q	P0CG48	UBC
361	Quercetin	2i7t	Q9UKF6	CPSF3
362	Quercetin	2wgh	P23921	RRM1
363	Quercetin	2f3i	P52434	POLR2H
364	Quercetin	2bxg	P02768	ALB
365	Quercetin	2vcy	Q9BV79	MECR
366	Quercetin	2aff	P46013	MKI67
367	Quercetin	2ejr	O60341	KDM1A
368	Quercetin	1wji	Q9H7E2	TDRD3
369	Quercetin	2z7x	Q15399	TLR1
370	Quercetin	2cpt	O75351	VPS4B
371	Quercetin	2ys5	O43559	FRS3
372	Quercetin	1lo1	O95718	ESRRB
373	Quercetin	3eh2	P53992	SEC24C
374	Quercetin	2c0o	P08631	HCK
375	Quercetin	3ifb	P12104	FABP2
376	Quercetin	3d8e	O00213	APBB1

377	Quercetin	1fsu	P15848	ARSB
378	Quercetin	3fay	P46940	IQGAP1
379	Quercetin	2hak	Q9P0L2	MARK1
380	Quercetin	2nzt	P52789	HK2
381	Quercetin	2da7	O60315	ZEB2
382	Quercetin	2csh	O43298	ZBTB43
383	Quercetin	1inz	Q9Y6I3	EPN1
384	Quercetin	1lar	P10586	PTPRF
385	Quercetin	1avf	P20142	PGC
386	Quercetin	1wi3	Q9UPW6	SATB2
387	Quercetin	2d86	Q9UKW4	VAV3
388	Quercetin	2fc9	P19338	NCL
389	Quercetin	1v16	P12694	BCKDHA
390	Pinocembrin	2dba	Q9H3U1	UNC45A
391	Pinocembrin	1uw4	Q9BZI7	UPF3B
392	Pinocembrin	2cra	Q92826	HOXB13
393	Pinocembrin	1d6v	P01834	IGKC
394	Pinocembrin	1so0	Q96C23	GALM
395	Pinocembrin	2bjn	Q86SZ2	TRAPPC6B
396	Pinocembrin	1wh0	O94966	USP19
397	Pinocembrin	1jfi	Q01658	DR1
398	Pinocembrin	2qq5	Q96LJ7	DHRS1
399	Pinocembrin	2cue	P26367	PAX6
400	Pinocembrin	2coa	Q9BZL6	PRKD2
401	Pinocembrin	1x86	Q9NZN5	ARHGEF12
402	Pinocembrin	3dpl	Q93034	CUL5
403	Pinocembrin	2ppi	Q9H2C0	GAN
404	Pinocembrin	2csw	O76064	RNF8
405	Pinocembrin	2ija	P18440	NAT1
406	Pinocembrin	1d5b	P01834	IGKC
407	Pinocembrin	2eb1	Q9UPY3	DICER1
408	Pinocembrin	1wry	O75368	SH3BGRL
409	Pinocembrin	1wi3	Q9UPW6	SATB2
410	Pinocembrin	3b68	P10275	AR
411	Pinocembrin	1lo1	O95718	ESRRB
412	Pinocembrin	1iau	P10144	GZMB
413	Pinocembrin	2vcy	Q9BV79	MECR
414	Pinocembrin	2fvl	P17516	AKR1C4
415	Pinocembrin	3ch6	P28845	HSD11B1
416	Pinocembrin	3gtu	P28161	GSTM2
417	Pinocembrin	1fe8	P04275	VWF
418	Pinocembrin	3ezt	Q9BY41	HDAC8
419	Pinocembrin	1rly	Q00403	GTF2B
420	Pinocembrin	2ql6	Q9NWW6	NMRK1
421	Pinocembrin	2dmq	Q9NQ69	LHX9
422	Pinocembrin	2aug	Q14449	GRB14
423	vitexin	2dba	Q9H3U1	UNC45A
424	vitexin	2qfd	O95786	DDX58
425	vitexin	1ujv	Q86UL8	MAGI2
426	vitexin	2ytr	Q96SE7	ZNF347
427	vitexin	1wym	P37802	TAGLN2
428	vitexin	1xjv	Q9NUX5	POT1
429	vitexin	2di8	O75369	FLNB
430	vitexin	3epz	P26358	DNMT1

431	vitexin	1uw4	Q9BZI7	UPF3B
432	vitexin	2fg5	Q13636	RAB31
433	vitexin	2p0r	O14815	CAPN9
434	vitexin	1z68	Q12884	FAP
435	vitexin	1cok	O15350	TP73
436	vitexin	1ua2	P50613	CDK7
437	vitexin	3d3l	P18054	ALOX12
438	vitexin	2wbi	Q709F0	ACAD11
439	vitexin	2iwg	P19474	TRIM21
440	vitexin	1fo3	Q9UKM7	MAN1B1
441	vitexin	2j7q	P0CG48	UBC
442	vitexin	1w98	P24864	CCNE1
443	vitexin	2yu4	Q96MF7	NSMCE2
444	vitexin	1k9k	P06703	S100A6
445	vitexin	1wh0	O94966	USP19
446	vitexin	2jun	O15344	MID1
447	vitexin	2j91	P30566	ADSL
448	vitexin	1unh	Q00535	CDK5
449	vitexin	3bsz	P02766	TTR
450	vitexin	2dlw	O60496	DOK2
451	vitexin	1ow1	Q96T58	SPEN
452	vitexin	3bkb	P07332	FES
453	vitexin	2a2d	Q01415	GALK2
454	vitexin	2dnk	Q9BZC1	CELF4
455	vitexin	2bu7	Q15119	PDK2
456	vitexin	2nn6	Q06265	EXOSC9
457	vitexin	2bc9	P32455	GBP1
458	vitexin	1q8k	P05198	EIF2S1
459	vitexin	2raj	Q9Y5X1	SNX9
460	vitexin	1b4f	P29323	EPHB2
461	vitexin	1jrh	P15260	IFNGR1
462	vitexin	2aff	P46013	MKI67
463	vitexin	2csw	O76064	RNF8
464	vitexin	2pom	Q15750	TAB1
465	vitexin	2da7	O60315	ZEB2
466	vitexin	2r55	Q9NSY2	STARD5
467	vitexin	2qqh	P07357	C8A
468	vitexin	2c0o	P08631	HCK
469	vitexin	1igr	P08069	IGF1R
470	vitexin	1inz	Q9Y6I3	EPN1
471	vitexin	1wch	Q12923	PTPN13
472	vitexin	2a98	Q96DU7	ITPKC
473	vitexin	1lar	P10586	PTPRF
474	vitexin	1h2t	P52298	NCBP2
475	vitexin	1mzd	P49863	GZMK
476	vitexin	3eht	P06850	CRH
477	vitexin	3czu	P20827	EFNA1
478	vitexin	3cww	P14735	IDE
479	vitexin	3eay	Q9BQF6	SENP7
480	vitexin	1exv	P06737	PYGL
481	vitexin	3b68	P10275	AR
482	vitexin	1onq	P06126	CD1A
483	vitexin	2r37	P22352	GPX3
484	vitexin	1zag	P25311	AZGP1

485	vitexin	3bpt	Q6NVY1	HIBCH
486	vitexin	1wi3	Q9UPW6	SATB2
487	vitexin	3eg9	Q15436	SEC23A
488	vitexin	2i7n	Q8TE04	PANK1
489	vitexin	1ntg	P54577	YARS1
490	vitexin	3cb2	P23258	TUBG1
491	vitexin	2yum	Q8IYH5	ZZZ3
492	Kaempherol	3fer	O75369	FLNB
493	Kaempherol	2de0	Q9BYC5	FUT8
494	Kaempherol	2eon	Q9Y2L8	ZKSCAN5
495	Kaempherol	3fmo	Q9UMR2	DDX19B
496	Kaempherol	3cki	P78536	ADAM17
497	Kaempherol	2h63	P53004	BLVRA
498	Kaempherol	2ahx	Q15303	ERBB4
499	Kaempherol	1s9i	P36507	MAP2K2
500	Kaempherol	1x86	Q9NZN5	ARHGEF12
501	Kaempherol	2cra	Q92826	HOXB13
502	Kaempherol	2ppi	Q9H2C0	GAN
503	Kaempherol	2a2d	Q01415	GALK2
504	Kaempherol	1kv3	P21980	TGM2
505	Kaempherol	2coo	P11182	DBT
506	Kaempherol	2coa	Q9BZL6	PRKD2
507	Kaempherol	1d5b	P01834	IGKC
508	Kaempherol	2v62	Q86Y07	VRK2
509	Kaempherol	1wji	Q9H7E2	TDRD3
510	Kaempherol	2enj	Q04759	PRKCQ
511	Kaempherol	2j21	P04637	TP53
512	Kaempherol	2f3i	P52434	POLR2H
513	Kaempherol	2ejr	O60341	KDM1A
514	Kaempherol	2aff	P46013	MKI67
515	Kaempherol	2i7t	Q9UKF6	CPSF3
516	Kaempherol	2j7q	P0CG48	UBC
517	Kaempherol	2wgh	P23921	RRM1
518	Kaempherol	2pom	Q15750	TAB1
519	Kaempherol	2vcy	Q9BV79	MECR
520	Kaempherol	1wi5	Q14690	PDCD11
521	Kaempherol	2w96	P11802	CDK4
522	Kaempherol	1w6j	P48449	LSS
523	Kaempherol	2c9o	Q9Y265	RUVBL1
524	Kaempherol	2cpt	O75351	VPS4B
525	Kaempherol	2ys5	O43559	FRS3
526	Kaempherol	3enm	P52564	MAP2K6
527	Kaempherol	2jrj	Q96PM5	RCHY1
528	Kaempherol	1fe8	P04275	VWF
529	Kaempherol	2c0o	P08631	HCK
530	Kaempherol	3ifb	P12104	FABP2
531	Kaempherol	3eh2	P53992	SEC24C
532	Kaempherol	1vyh	P68402	PAFAH1B2
533	Kaempherol	2dl8	O75044	SRGAP2
534	Kaempherol	1hlg	P07098	LIPF
535	Kaempherol	2kbq	Q9Y6N9	USH1C
536	Kaempherol	1fsu	P15848	ARSB
537	Kaempherol	1onq	P06126	CD1A
538	Kaempherol	2hak	Q9P0L2	MARK1

539	Kaempherol	1inz	Q9Y6I3	EPN1
540	Naringenin	2dba	Q9H3U1	UNC45A
541	Naringenin	3fmo	Q9UMR2	DDX19B
542	Naringenin	3c4m	P01270	PTH
543	Naringenin	2aug	Q14449	GRB14
544	Naringenin	2aii	Q8NBK3	SUMF1
545	Naringenin	1fcx	P13631	RARG
546	Naringenin	2p0r	O14815	CAPN9
547	Naringenin	1x86	Q9NZN5	ARHGEF12
548	Naringenin	2raj	Q9Y5X1	SNX9
549	Naringenin	2ppi	Q9H2C0	GAN
550	Naringenin	2f3i	P52434	POLR2H
551	Naringenin	1wry	O75368	SH3BGRL
552	Naringenin	2ozb	P55769	SNU13
553	Naringenin	1pkx	P31939	ATIC
554	Naringenin	3bfx	O00338	SULT1C2
555	Naringenin	1zag	P25311	AZGP1
556	Naringenin	1nrg	Q9NVS9	PNPO
557	Naringenin	2ep8	O00541	PES1
558	Naringenin	1pve	P54727	RAD23B
559	Naringenin	1wuu	P51570	GALK1
560	Naringenin	2eb1	Q9UPY3	DICER1
561	Naringenin	1d5b	P01834	IGKC
562	Naringenin	2ija	P18440	NAT1
563	Naringenin	2csw	O76064	RNF8
564	Naringenin	3bg0	P55735	SEC13
565	Naringenin	1inz	Q9Y6I3	EPN1
566	Naringenin	1fe8	P04275	VWF
567	Naringenin	1mj4	P51687	SUOX
568	Naringenin	3c5k	Q9UBN7	HDAC6
569	Naringenin	1wi3	Q9UPW6	SATB2
570	Naringenin	3enm	P52564	MAP2K6
571	Naringenin	1w98	P24864	CCNE1
572	Naringenin	1vyh	P68402	PAFAH1B2
573	Naringenin	2eov	Q5JVG2	ZNF484
574	Naringenin	2dl8	O75044	SRGAP2
575	Morin	2vre	Q13011	ECH1
576	Morin	3fer	O75369	FLNB
577	Morin	1snl	Q02818	NUCB1
578	Morin	3cki	P78536	ADAM17
579	Morin	1s9i	P36507	MAP2K2
580	Morin	2h63	P53004	BLVRA
581	Morin	1ivh	P26440	IVD
582	Morin	2q5e	O14595	CTDSP2
583	Morin	1mje	P60896	SEM1
584	Morin	2ahx	Q15303	ERBB4
585	Morin	2pkt	O00151	PDLIM1
586	Morin	1kv3	P21980	TGM2
587	Morin	1aoa	P13797	PLS3
588	Morin	1so0	Q96C23	GALM
589	Morin	2p0r	O14815	CAPN9
590	Morin	1exv	P06737	PYGL
591	Morin	3dl2	Q8IX04	UEVLD
592	Morin	2iki	P15121	AKR1B1

593	Morin	2psn	P06733	ENO1
594	Morin	2f57	Q9P286	PAK5
595	Morin	2ppi	Q9H2C0	GAN
596	Morin	1x86	Q9NZN5	ARHGEF12
597	Morin	1nfb	P12268	IMPDH2
598	Morin	1fg9	P15260	IFNGR1
599	Morin	2c2h	P60763	RAC3
600	Morin	2yu4	Q96MF7	NSMCE2
601	Morin	2ig7	Q9Y259	CHKB
602	Morin	1l9x	Q92820	GGH
603	Morin	3e4e	P05181	CYP2E1
604	Morin	2wgh	P23921	RRM1
605	Morin	1rxt	P30419	NMT1
606	Morin	1qib	P08253	MMP2
607	Morin	2fxr	P20231	TPSB2
608	Morin	2coo	P11182	DBT
609	Morin	2coa	Q9BZL6	PRKD2
610	Morin	1zag	P25311	AZGP1
611	Morin	1jey	P12956	XRCC6
612	Morin	1wji	Q9H7E2	TDRD3
613	Morin	2enj	Q04759	PRKCQ
614	Morin	1pkx	P31939	ATIC
615	Morin	2vr2	Q14117	DPYS
616	Morin	2e6i	Q08881	ITK
617	Morin	2gl8	P48443	RXRG
618	Morin	1d5b	P01834	IGKC
619	Morin	2dmz	Q8NI35	PATJ
620	Morin	1vyh	P68402	PAFAH1B2
621	Morin	1w45	P13928	ANXA8
622	Morin	1lw3	Q13614	MTMR2
623	Morin	2fg5	Q13636	RAB31
624	Morin	2yt7	O96018	APBA3
625	Morin	1lo1	O95718	ESRRB
626	Morin	2p39	Q9GZV9	FGF23
627	Morin	2ysd	Q96QZ7	MAGI1
628	Morin	3dpl	Q93034	CUL5
629	Morin	1ef7	Q9UBR2	CTSZ
630	Morin	2eje	P78347	GTF2I
631	Morin	2c9o	Q9Y265	RUVBL1
632	Morin	1oll	O76036	NCR1
633	Morin	3ebb	P55072	VCP
634	Morin	1w60	P12004	PCNA
635	Morin	2j7q	P0CG48	UBC
636	Morin	2ejr	O60341	KDM1A
637	Morin	2f3i	P52434	POLR2H
638	Morin	2nn6	Q06265	EXOSC9
639	Morin	2aff	P46013	MKI67
640	Morin	2pla	Q8N335	GPD1L
641	Morin	2fw2	Q9Y6F7	CDY2A
642	Morin	1bj1	P15692	VEGFA
643	Morin	2coz	Q5VT06	CEP350
644	Morin	2bu7	Q15119	PDK2
645	Morin	1lar	P10586	PTPRF
646	Morin	1mzd	P49863	GZMK

647	Morin	2c0o	P08631	HCK
648	Morin	1gqm	P80511	S100A12
649	Morin	3ifb	P12104	FABP2
650	Morin	3cok	O00444	PLK4
651	Morin	1qfw	P01215	CGA
652	Morin	2a98	Q96DU7	ITPKC
653	Morin	3eh2	P53992	SEC24C
654	Morin	3d4j	P53602	MVD
655	Myricetin	3fer	O75369	FLNB
656	Myricetin	2de0	Q9BYC5	FUT8
657	Myricetin	1x66	Q01543	FLI1
658	Myricetin	3cki	P78536	ADAM17
659	Myricetin	2aug	Q14449	GRB14
660	Myricetin	2ocb	Q9NP90	RAB9B
661	Myricetin	3bo5	Q53H47	SETMAR
662	Myricetin	2aii	Q8NBK3	SUMF1
663	Myricetin	1wwv	Q969H4	CNKSR1
664	Myricetin	2h63	P53004	BLVRA
665	Myricetin	1wm5	P19878	NCF2
666	Myricetin	1wx7	Q9H347	UBQLN3
667	Myricetin	1ln3	Q9UKL6	PCTP
668	Myricetin	2ahx	Q15303	ERBB4
669	Myricetin	3dpl	Q93034	CUL5
670	Myricetin	2coa	Q9BZL6	PRKD2
671	Myricetin	1x86	Q9NZN5	ARHGEF12
672	Myricetin	1wi5	Q14690	PDCD11
673	Myricetin	3bg1	P55735	SEC13
674	Myricetin	1kgd	O14936	CASK
675	Myricetin	1uwy	P14384	CPM
676	Myricetin	2ppi	Q9H2C0	GAN
677	Myricetin	2grp	P46060	RANGAP1
678	Myricetin	2csw	O76064	RNF8
679	Myricetin	1szb	O00187	MASP2
680	Myricetin	3bfx	O00338	SULT1C2
681	Myricetin	1b4f	P29323	EPHB2
682	Myricetin	2coo	P11182	DBT
683	Myricetin	1fe8	P04275	VWF
684	Myricetin	2fy4	P28329	CHAT
685	Myricetin	2dnk	Q9BZC1	CELF4
686	Myricetin	2ad1	O75897	SULT1C4
687	Myricetin	1ow1	Q96T58	SPEN
688	Myricetin	2raj	Q9Y5X1	SNX9
689	Myricetin	2cpt	O75351	VPS4B
690	Myricetin	3g65	O60671	RAD1
691	Myricetin	2avd	Q86VU5	COMTD1
692	Myricetin	7ics	P06746	POLB
693	Myricetin	2j7q	P0CG48	UBC
694	Myricetin	2f8n	O75367	MACROH2A1
695	Myricetin	1pkx	P31939	ATIC
696	Myricetin	3enm	P52564	MAP2K6
697	Myricetin	2ys5	O43559	FRS3
698	Myricetin	2bxg	P02768	ALB
699	Myricetin	2z7x	Q15399	TLR1
700	Myricetin	2h8r	P35680	HNF1B

701	Myricetin	1d5b	P01834	IGKC
702	Myricetin	2ysd	Q96QZ7	MAGI1
703	Myricetin	1x7z	P12694	BCKDHA
704	Myricetin	1wry	O75368	SH3BGRL
705	Myricetin	2f3i	P52434	POLR2H
706	Myricetin	2aff	P46013	MKI67
707	Myricetin	2ejr	O60341	KDM1A
708	Myricetin	3eh2	P53992	SEC24C
709	Myricetin	3bpt	Q6NVY1	HIBCH
710	Myricetin	3ifb	P12104	FABP2
711	Myricetin	2aa7	P08235	NR3C2
712	Myricetin	2vwe	P49765	VEGFB
713	Myricetin	2d86	Q9UKW4	VAV3
714	Myricetin	2da7	O60315	ZEB2
715	Myricetin	1s31	P50607	TUB
716	Myricetin	2csh	O43298	ZBTB43
717	Myricetin	3fay	P46940	IQGAP1
718	Myricetin	1fsu	P15848	ARSB
719	Myricetin	2hak	Q9P0L2	MARK1
720	Myricetin	2zot	Q9HCB6	SPON1
721	Myricetin	1igr	P08069	IGF1R
722	Hesperidin	2p0r	O14815	CAPN9
723	Hesperidin	2wa0	P43358	MAGEA4
724	Hesperidin	1xjv	Q9NUX5	POT1
725	Hesperidin	1em2	Q14849	STARD3
726	Hesperidin	1x65	O75534	CSDE1
727	Hesperidin	2e1o	Q03014	HHEX
728	Hesperidin	2cmw	Q9HCP0	CSNK1G1
729	Hesperidin	1x7z	P12694	BCKDHA
730	Hesperidin	2nn6	Q06265	EXOSC9
731	Hesperidin	1qib	P08253	MMP2
732	Hesperidin	1xap	P10826	RARB
733	Hesperidin	3bkb	P07332	FES
734	Hesperidin	2jun	O15344	MID1
735	Hesperidin	2zme	Q86VN1	VPS36
736	Hesperidin	2csw	O76064	RNF8
737	Hesperidin	3bky	P11836	MS4A1
738	Hesperidin	2pla	Q8N335	GPD1L
739	Hesperidin	2d9b	P78347	GTF2I
740	Hesperidin	2d9w	O60496	DOK2
741	Hesperidin	2da7	O60315	ZEB2
742	Hesperidin	2ep8	O00541	PES1
743	Hesperidin	2nzt	P52789	HK2
744	Hesperidin	3epy	Q8N6N7	ACBD7
745	Hesperidin	1prx	P30041	PRDX6
746	Hesperidin	2okk	Q05329	GAD2
747	Hesperidin	2csh	O43298	ZBTB43
748	Hesperidin	2ed7	P43146	DCC
749	Hesperidin	1mj4	P51687	SUOX
750	Hesperidin	2eec	O75369	FLNB
751	Hesperidin	1s9c	P51659	HSD17B4
752	Hesperidin	1bj1	P15692	VEGFA
753	Hesperidin	1wfo	Q58EX2	SDK2
754	Hesperidin	3dax	P22680	CYP7A1

755	Hesperidin	1h2t	P52298	NCBP2
756	Hesperidin	3czu	P20827	EFNA1
757	Hesperidin	2v9k	Q3MIT2	PUS10
758	Hesperidin	2c62	P53999	SUB1
759	Hesperidin	2iwg	P19474	TRIM21
760	Hesperidin	2cls	Q92730	RND1
761	Hesperidin	3e3r	Q13938	CAPS
762	Hesperidin	1inz	Q9Y6I3	EPN1
763	Hesperidin	2qq5	Q96LJ7	DHRS1
764	Hesperidin	1x5p	P18615	NELFE
765	Hesperidin	1wuu	P51570	GALK1
766	Hesperidin	2h8r	P35680	HNF1B
767	Hesperidin	1cqt	P14859	POU2F1
768	Hesperidin	2ji4	O60256	PRPSAP2
769	Hesperidin	2pp4	Q06455	RUNX1T1
770	Hesperidin	2a98	Q96DU7	ITPKC
771	Hesperidin	3f0w	Q9Y6R0	NUMBL
772	Hesperidin	2bzl	Q15678	PTPN14
773	Hesperidin	1y4j	Q8NBJ7	SUMF2
774	Hesperidin	1pve	P54727	RAD23B
775	Hesperidin	3ezt	Q9BY41	HDAC8
776	Hesperidin	2bxg	P02768	ALB
777	Hesperidin	1sy6	P07766	CD3E
778	Hesperidin	2cs8	O60284	ST18
779	Hesperidin	1xfb	P09972	ALDOC
780	Hesperidin	3brw	P47736	RAP1GAP
781	Hesperidin	1e9a	P23919	DTYMK
782	Hesperidin	1spj	P06870	KLK1
783	Hesperidin	2cu7	Q5VVJ2	MYSM1
784	Hesperidin	1jey	P12956	XRCC6
785	Hesperidin	2yum	Q8IYH5	ZZZ3
786	Hesperidin	1r4m	Q8TBC4	UBA3
787	Hesperidin	1fcg	P12318	FCGR2A
788	Hesperidin	2ee4	Q13017	ARHGAP5
789	Hesperidin	1z2c	P08134	RHOC
790	Hesperidin	2csq	O15034	RIMBP2
791	Hesperidin	1fyv	Q15399	TLR1
792	Hesperidin	2vz6	Q9UQM7	CAMK2A
793	Daidzein	2qfd	O95786	DDX58
794	Daidzein	2i7k	Q9NPI1	BRD7
795	Daidzein	2dba	Q9H3U1	UNC45A
796	Daidzein	1em2	Q14849	STARD3
797	Daidzein	1so0	Q96C23	GALM
798	Daidzein	2f3i	P52434	POLR2H
799	Daidzein	1wh0	O94966	USP19
800	Daidzein	1x65	O75534	CSDE1
801	Daidzein	2cue	P26367	PAX6
802	Daidzein	2d2z	Q9Y696	CLIC4
803	Daidzein	1zag	P25311	AZGP1
804	Daidzein	2ozb	P55769	SNU13
805	Daidzein	2raj	Q9Y5X1	SNX9
806	Daidzein	1s9i	P36507	MAP2K2
807	Daidzein	1x86	Q9NZN5	ARHGEF12
808	Daidzein	2ad1	O75897	SULT1C4

809	Daidzein	3gtu	P28161	GSTM2
810	Daidzein	2ija	P18440	NAT1
811	Daidzein	3b68	P10275	AR
812	Daidzein	3bpt	Q6NVY1	HIBCH
813	Daidzein	2arp	P08476	INHBA
814	Daidzein	1wi3	Q9UPW6	SATB2
815	Daidzein	2nn6	Q06265	EXOSC9
816	Daidzein	2nmp	P32929	CTH
817	Daidzein	1rkh	Q15648	MED1
818	Daidzein	1ya0	Q92540	SMG7
819	Daidzein	2ql6	Q9NWW6	NMRK1
820	Daidzein	3bum	O43597	SPRY2
821	Daidzein	2r55	Q9NSY2	STARD5
822	Daidzein	3eay	Q9BQF6	SENP7
823	Daidzein	2dmq	Q9NQ69	LHX9
824	lycoperodine-1	1z9e	O75475	PSIP1
825	lycoperodine-1	2nn6	Q06265	EXOSC9
826	lycoperodine-1	2a2d	Q01415	GALK2
827	lycoperodine-1	1s31	P50607	TUB
828	lycoperodine-1	3cun	P09012	SNRPA
829	lycoperodine-1	2eon	Q9Y2L8	ZKSCAN5
830	lycoperodine-1	1s9i	P36507	MAP2K2
831	lycoperodine-1	3dlj	Q96KN2	CNDP1
832	lycoperodine-1	1aoa	P13797	PLS3
833	lycoperodine-1	1pkg	P10721	KIT
834	lycoperodine-1	3dwb	P42892	ECE1
835	lycoperodine-1	1ow1	Q96T58	SPEN
836	lycoperodine-1	1x68	Q5TD97	FHL5
837	lycoperodine-1	2qp4	Q06520	SULT2A1
838	lycoperodine-1	2pkt	O00151	PDLIM1
839	lycoperodine-1	2iwg	P19474	TRIM21
840	lycoperodine-1	1w98	P24864	CCNE1
841	lycoperodine-1	1wgy	Q92565	RAPGEF5
842	lycoperodine-1	2ejr	O60341	KDM1A
843	lycoperodine-1	2b3y	P21399	ACO1
844	lycoperodine-1	1c9b	Q00403	GTF2B
845	lycoperodine-1	2e29	Q9BQ39	DDX50
846	lycoperodine-1	1fe8	P04275	VWF
847	lycoperodine-1	3d3l	P18054	ALOX12
848	lycoperodine-1	2err	Q9NWB1	RBFOX1
849	lycoperodine-1	2he4	Q15599	SLC9A3R2
850	lycoperodine-1	2o3q	P28907	CD38
851	lycoperodine-1	2zw3	P29033	GJB2
852	lycoperodine-1	3bhy	O43293	DAPK3
853	lycoperodine-1	1inz	Q9Y6I3	EPN1
854	lycoperodine-1	1ujv	Q86UL8	MAGI2
855	lycoperodine-1	2cue	P26367	PAX6
856	lycoperodine-1	1ry7	P05230	FGF1
857	lycoperodine-1	1w45	P13928	ANXA8
858	lycoperodine-1	3eay	Q9BQF6	SENP7
859	lycoperodine-1	3bky	P11836	MS4A1
860	lycoperodine-1	2eje	P78347	GTF2I
861	lycoperodine-1	2h08	P60891	PRPS1
862	lycoperodine-1	1x1f	Q9ULZ2	STAP1

863	lycoperodine-1	3b68	P10275	AR
864	lycoperodine-1	2hzp	Q16719	KYNU
865	lycoperodine-1	1jmj	P05546	SERPIND1
866	lycoperodine-1	1us1	Q16853	AOC3
867	lycoperodine-1	3c5k	Q9UBN7	HDAC6
868	lycoperodine-1	2psn	P06733	ENO1
869	lycoperodine-1	2d9p	Q9H361	PABPC3
870	lycoperodine-1	2i7t	Q9UKF6	CPSF3
871	lycoperodine-1	3e7o	P45984	MAPK9
872	lycoperodine-1	2fxr	P20231	TPSB2
873	lycoperodine-1	1unh	Q00535	CDK5
874	lycoperodine-1	2vr2	Q14117	DPYS
875	lycoperodine-1	2e44	O00425	IGF2BP3
876	lycoperodine-1	2okk	Q05329	GAD2
877	lycoperodine-1	1rkh	Q15648	MED1
878	lycoperodine-1	2pp4	Q06455	RUNX1T1
879	lycoperodine-1	1gh7	P32927	CSF2RB
880	lycoperodine-1	2vkq	Q9H0P0	NT5C3A
881	lycoperodine-1	1l9x	Q92820	GGH
882	lycoperodine-1	2ayn	P54578	USP14
883	lycoperodine-1	3c10	Q8WUI4	HDAC7
884	lycoperodine-1	2cmw	Q9HCP0	CSNK1G1
885	lycoperodine-1	3dax	P22680	CYP7A1
886	lycoperodine-1	2bxr	P21397	MAOA
887	lycoperodine-1	2fg5	Q13636	RAB31
888	lycoperodine-1	2qqh	P07357	C8A
889	lycoperodine-1	3bsz	P02766	TTR
890	lycoperodine-1	2kbq	Q9Y6N9	USH1C
891	lycoperodine-1	3hd6	Q9UBD6	RHCG
892	catechin	1qz2	Q02790	FKBP4
893	catechin	2dba	Q9H3U1	UNC45A
894	catechin	2i7k	Q9NPI1	BRD7
895	catechin	1egw	Q02078	MEF2A
896	catechin	1x65	O75534	CSDE1
897	catechin	2acx	P43250	GRK6
898	catechin	3dxf	Q9HBL8	NMRAL1
899	catechin	2i7t	Q9UKF6	CPSF3
900	catechin	3d7d	Q04609	FOLH1
901	catechin	1sy6	P07766	CD3E
902	catechin	2cog	P54687	BCAT1
903	catechin	1xjv	Q9NUX5	POT1
904	catechin	1fgu	P27694	RPA1
905	catechin	3fmo	Q9UMR2	DDX19B
906	catechin	2b3y	P21399	ACO1
907	catechin	2p0r	O14815	CAPN9
908	catechin	1ggt	P00488	F13A1
909	catechin	1v9u	P98155	VLDLR
910	catechin	3c4m	P01270	PTH
911	catechin	1gzq	P29016	CD1B
912	catechin	1o9k	P06400	RB1
913	catechin	2vpk	Q9NPC7	MYNN
914	catechin	1buo	Q05516	ZBTB16
915	catechin	1em2	Q14849	STARD3
916	catechin	1fcx	P13631	RARG

917	catechin	2dk2	O43390	HNRNPR
918	catechin	1u1k	P09651	HNRNPA1
919	catechin	1m9i	P08133	ANXA6
920	catechin	2ozb	P55769	SNU13
921	catechin	3bg0	P55735	SEC13
922	catechin	1ow1	Q96T58	SPEN
923	catechin	2eb1	Q9UPY3	DICER1
924	catechin	3bkb	P07332	FES
925	catechin	1zag	P25311	AZGP1
926	catechin	2raj	Q9Y5X1	SNX9
927	catechin	2ppi	Q9H2C0	GAN
928	catechin	2aug	Q14449	GRB14
929	catechin	1z0j	Q9H1K0	RBSN
930	catechin	1d6v	P01834	IGKC
931	catechin	1l9x	Q92820	GGH
932	catechin	2ija	P18440	NAT1
933	catechin	2zme	Q86VN1	VPS36
934	catechin	3bfx	O00338	SULT1C2
935	catechin	3f5n	Q99574	SERPINI1
936	catechin	1wuu	P51570	GALK1
937	catechin	2pkt	O00151	PDLIM1
938	catechin	2gl8	P48443	RXRG
939	catechin	2f3i	P52434	POLR2H
940	catechin	1xap	P10826	RARB
941	catechin	3dpl	Q93034	CUL5
942	catechin	1igr	P08069	IGF1R
943	catechin	2j7q	P0CG48	UBC
944	catechin	1wry	O75368	SH3BGRL
945	catechin	1lpj	Q96R05	RBP7
946	catechin	3bpt	Q6NVY1	HIBCH
947	catechin	1wi3	Q9UPW6	SATB2
948	catechin	3cww	P14735	IDE
949	catechin	1inz	Q9Y6I3	EPN1
950	catechin	1mj4	P51687	SUOX
951	catechin	2iwg	P19474	TRIM21
952	catechin	1gqm	P80511	S100A12
953	catechin	3eay	Q9BQF6	SENP7
954	catechin	3bky	P11836	MS4A1
955	catechin	1w6j	P48449	LSS
956	catechin	1y8o	Q15120	PDK3
957	catechin	1v5w	Q14565	DMC1
958	catechin	2yum	Q8IYH5	ZZZ3
959	catechin	2c0o	P08631	HCK
960	catechin	2coa	Q9BZL6	PRKD2
961	catechin	2ewy	Q9Y5Z0	BACE2
962	Epigallocatechin gallate	2dgu	O60506	SYNCRIP
963	Epigallocatechin gallate	2vpk	Q9NPC7	MYNN
964	Epigallocatechin gallate	1w98	P24864	CCNE1
965	Epigallocatechin gallate	3bkb	P07332	FES
966	Epigallocatechin gallate	1x65	O75534	CSDE1
967	Epigallocatechin gallate	1zag	P25311	AZGP1
968	Epigallocatechin gallate	3dpl	Q93034	CUL5
969	Epigallocatechin gallate	3epz	P26358	DNMT1
970	Epigallocatechin gallate	2ppi	Q9H2C0	GAN

971	Epigallocatechin gallate	2grp	P46060	RANGAP1
972	Epigallocatechin gallate	2da7	O60315	ZEB2
973	Epigallocatechin gallate	1u6g	Q13616	CUL1
974	Epigallocatechin gallate	2nn6	Q06265	EXOSC9
975	Epigallocatechin gallate	3c5k	Q9UBN7	HDAC6
976	Epigallocatechin gallate	1d5b	P01834	IGKC
977	Epigallocatechin gallate	3eay	Q9BQF6	SENP7
978	Epigallocatechin gallate	3e4e	P05181	CYP2E1
979	Epigallocatechin gallate	2csh	O43298	ZBTB43
980	Epigallocatechin gallate	2iwg	P19474	TRIM21
981	Epigallocatechin gallate	1ow1	Q96T58	SPEN
982	Epigallocatechin gallate	2o06	P19623	SRM
983	Epigallocatechin gallate	3cok	O00444	PLK4
984	Epigallocatechin gallate	1mj4	P51687	SUOX
985	Epigallocatechin gallate	1bj1	P15692	VEGFA
986	Epigallocatechin gallate	2pph	Q99759	MAP3K3
987	Epigallocatechin gallate	1k5g	P43487	RANBP1
988	Epigallocatechin gallate	2hzp	Q16719	KYNU
989	Epigallocatechin gallate	1t9g	P11310	ACADM
990	Epigallocatechin gallate	3c10	Q8WUI4	HDAC7
991	Epigallocatechin gallate	2yrb	Q68CZ1	RPGRIP1L
992	Epigallocatechin gallate	1x51	Q9UIF7	MUTYH
993	Epigallocatechin gallate	1w6j	P48449	LSS
994	Epigallocatechin gallate	1qib	P08253	MMP2
995	Epigallocatechin gallate	1h2t	P52298	NCBP2
996	Epigallocatechin gallate	2gow	O95164	UBL3
997	Epigallocatechin gallate	1inz	Q9Y6I3	EPN1
998	Epigallocatechin gallate	2eec	O75369	FLNB
999	Epigallocatechin gallate	2vwe	P49765	VEGFB
1000	Epigallocatechin gallate	2nzt	P52789	HK2
1001	Epigallocatechin gallate	2a98	Q96DU7	ITPKC
1002	Epigallocatechin gallate	1wi3	Q9UPW6	SATB2
1003	Epigallocatechin gallate	2vz6	Q9UQM7	CAMK2A
1004	Epigallocatechin gallate	1x5p	P18615	NELFE
1005	Epigallocatechin gallate	1ya0	Q92540	SMG7
1006	Epigallocatechin gallate	2ee4	Q13017	ARHGAP5
1007	Epigallocatechin gallate	1pve	P54727	RAD23B
1008	Epigallocatechin gallate	2dlw	O60496	DOK2
1009	Epigallocatechin gallate	2do1	P82979	SARNP
1010	Epigallocatechin gallate	1sy6	P07766	CD3E
1011	Epigallocatechin gallate	2ql6	Q9NWW6	NMRK1
1012	Epigallocatechin gallate	1igr	P08069	IGF1R
1013	Epigallocatechin gallate	2emj	Q8NHY6	ZFP28
1014	Epigallocatechin gallate	1y4j	Q8NBJ7	SUMF2
1015	Epigallocatechin gallate	3c0r	P0CG48	UBC
1016	Epigallocatechin gallate	2h8r	P35680	HNF1B
1017	Epigallocatechin gallate	3bpt	Q6NVY1	HIBCH
1018	Epigallocatechin gallate	2bsk	P62072	TIMM10
1019	Epigallocatechin gallate	1wl4	Q9BWD1	ACAT2
1020	chlorogenic acid	1hxm	B7Z8K6	TRDC
1021	chlorogenic acid	1wym	P37802	TAGLN2
1022	chlorogenic acid	3dpl	Q93034	CUL5
1023	chlorogenic acid	2c9o	Q9Y265	RUVBL1
1024	chlorogenic acid	1fxy	P00742	F10

1025	chlorogenic acid	1w6j	P48449	LSS
1026	chlorogenic acid	1ymm	P01903	HLA-DRA
1027	chlorogenic acid	1wgy	Q92565	RAPGEF5
1028	chlorogenic acid	1wm5	P19878	NCF2
1029	chlorogenic acid	3fe1	P17066	HSPA6
1030	chlorogenic acid	2rdw	Q9UJM8	HAO1
1031	chlorogenic acid	2da7	O60315	ZEB2
1032	chlorogenic acid	2dlu	Q8NI35	PATJ
1033	chlorogenic acid	1ow1	Q96T58	SPEN
1034	chlorogenic acid	2c2h	P60763	RAC3
1035	chlorogenic acid	3bkb	P07332	FES
1036	chlorogenic acid	2fg5	Q13636	RAB31
1037	chlorogenic acid	1q8k	P05198	EIF2S1
1038	chlorogenic acid	2wgh	P23921	RRM1
1039	chlorogenic acid	2vrw	P63000	RAC1
1040	chlorogenic acid	1rt9	P00491	PNP
1041	chlorogenic acid	2kdf	P55036	PSMD4
1042	chlorogenic acid	1w60	P12004	PCNA
1043	chlorogenic acid	2ejr	O60341	KDM1A
1044	chlorogenic acid	2ad1	O75897	SULT1C4
1045	chlorogenic acid	3g1n	Q7Z6Z7	HUWE1
1046	chlorogenic acid	2okk	Q05329	GAD2
1047	chlorogenic acid	2ysd	Q96QZ7	MAGI1
1048	chlorogenic acid	1szb	O00187	MASP2
1049	chlorogenic acid	2j7q	P0CG48	UBC
1050	chlorogenic acid	2p39	Q9GZV9	FGF23
1051	chlorogenic acid	2pla	Q8N335	GPD1L
1052	chlorogenic acid	2raj	Q9Y5X1	SNX9
1053	chlorogenic acid	2jun	O15344	MID1
1054	chlorogenic acid	3enm	P52564	MAP2K6
1055	chlorogenic acid	3bg1	P55735	SEC13
1056	chlorogenic acid	2kbq	Q9Y6N9	USH1C
1057	chlorogenic acid	1nrg	Q9NVS9	PNPO
1058	chlorogenic acid	2eje	P78347	GTF2I
1059	chlorogenic acid	1k9i	Q9NNX6	CD209
1060	chlorogenic acid	1lo1	O95718	ESRRB
1061	chlorogenic acid	2csw	O76064	RNF8
1062	chlorogenic acid	1w9c	O14980	XPO1
1063	chlorogenic acid	2fh1	P06396	GSN
1064	chlorogenic acid	2i6a	P55263	ADK
1065	chlorogenic acid	3gcx	P01130	LDLR
1066	chlorogenic acid	1pj4	P23368	ME2
1067	chlorogenic acid	1x6f	Q96JM2	ZNF462
1068	chlorogenic acid	1cqt	P14859	POU2F1
1069	chlorogenic acid	2bsk	P62072	TIMM10
1070	chlorogenic acid	1exv	P06737	PYGL
1071	chlorogenic acid	3eg9	Q15436	SEC23A
1072	chlorogenic acid	2c0o	P08631	HCK
1073	chlorogenic acid	1ugk	Q9H2B2	SYT4
1074	chlorogenic acid	2dos	P54252	ATXN3
1075	chlorogenic acid	2nn6	Q06265	EXOSC9
1076	chlorogenic acid	2gow	O95164	UBL3
1077	chlorogenic acid	2dsq	P08833	IGFBP1
1078	chlorogenic acid	2cql	P32969	RPL9

1079	chlorogenic acid	2i75	P29074	PTPN4
1080	chlorogenic acid	3d8e	O00213	APBB1
1081	chlorogenic acid	1puf	P40424	PBX1
1082	chlorogenic acid	2dmf	Q96BH1	RNF25
1083	chlorogenic acid	2uzg	Q8TEY7	USP33
1084	chlorogenic acid	2acx	P43250	GRK6
1085	chlorogenic acid	2ast	P46527	CDKN1B
1086	chlorogenic acid	3dax	P22680	CYP7A1
1087	epicatechin	2dba	Q9H3U1	UNC45A
1088	epicatechin	1qz2	Q02790	FKBP4
1089	epicatechin	1i1r	P40189	IL6ST
1090	epicatechin	2acx	P43250	GRK6
1091	epicatechin	3dxf	Q9HBL8	NMRAL1
1092	epicatechin	1x65	O75534	CSDE1
1093	epicatechin	2i7t	Q9UKF6	CPSF3
1094	epicatechin	2nn6	Q06265	EXOSC9
1095	epicatechin	1egw	Q02078	MEF2A
1096	epicatechin	3fmo	Q9UMR2	DDX19B
1097	epicatechin	3d7d	Q04609	FOLH1
1098	epicatechin	2cog	P54687	BCAT1
1099	epicatechin	3d3l	P18054	ALOX12
1100	epicatechin	3ch6	P28845	HSD11B1
1101	epicatechin	1d6v	P01834	IGKC
1102	epicatechin	1uw4	Q9BZI7	UPF3B
1103	epicatechin	1ggt	P00488	F13A1
1104	epicatechin	1gzq	P29016	CD1B
1105	epicatechin	1buo	Q05516	ZBTB16
1106	epicatechin	1o9k	P06400	RB1
1107	epicatechin	2vpk	Q9NPC7	MYNN
1108	epicatechin	2dk2	O43390	HNRNPR
1109	epicatechin	1em2	Q14849	STARD3
1110	epicatechin	1u1k	P09651	HNRNPA1
1111	epicatechin	1m9i	P08133	ANXA6
1112	epicatechin	1fcx	P13631	RARG
1113	epicatechin	1ivh	P26440	IVD
1114	epicatechin	2p0r	O14815	CAPN9
1115	epicatechin	1cok	O15350	TP73
1116	epicatechin	2ozb	P55769	SNU13
1117	epicatechin	1zag	P25311	AZGP1
1118	epicatechin	3dpl	Q93034	CUL5
1119	epicatechin	1x7z	P12694	BCKDHA
1120	epicatechin	1fe8	P04275	VWF
1121	epicatechin	2eb1	Q9UPY3	DICER1
1122	epicatechin	1z0j	Q9H1K0	RBSN
1123	epicatechin	2aug	Q14449	GRB14
1124	epicatechin	2raj	Q9Y5X1	SNX9
1125	epicatechin	2vze	Q08AH3	ACSM2A
1126	epicatechin	2ija	P18440	NAT1
1127	epicatechin	2gl8	P48443	RXRG
1128	epicatechin	2f3i	P52434	POLR2H
1129	epicatechin	1ow1	Q96T58	SPEN
1130	epicatechin	2ppi	Q9H2C0	GAN
1131	epicatechin	2zme	Q86VN1	VPS36
1132	epicatechin	2cpt	O75351	VPS4B

1133	epicatechin	2coa	Q9BZL6	PRKD2
1134	epicatechin	3bfx	O00338	SULT1C2
1135	epicatechin	2j7q	P0CG48	UBC
1136	epicatechin	1h2t	P52298	NCBP2
1137	epicatechin	1xap	P10826	RARB
1138	epicatechin	1b4f	P29323	EPHB2
1139	epicatechin	2csw	O76064	RNF8
1140	epicatechin	3bpt	Q6NVY1	HIBCH
1141	epicatechin	3eay	Q9BQF6	SENP7
1142	epicatechin	1w98	P24864	CCNE1
1143	epicatechin	1wi3	Q9UPW6	SATB2
1144	epicatechin	2vwe	P49765	VEGFB
1145	epicatechin	2r55	Q9NSY2	STARD5
1146	epicatechin	2coo	P11182	DBT
1147	epicatechin	1tzs	P14091	CTSE
1148	epicatechin	1x4v	Q8WV99	ZFAND2B
1149	epicatechin	1inz	Q9Y6I3	EPN1
1150	epicatechin	1lar	P10586	PTPRF
1151	epicatechin	2bug	P07900	HSP90AA1
1152	epicatechin	1mj4	P51687	SUOX
1153	epicatechin	2iwg	P19474	TRIM21
1154	epicatechin	1w45	P13928	ANXA8
1155	epicatechin	3cww	P14735	IDE
1156	epicatechin	2fju	P63000	RAC1
1157	epicatechin	1w6j	P48449	LSS
1158	epicatechin	3bg0	P55735	SEC13
1159	ellagic acid	1d6v	P01834	IGKC
1160	ellagic acid	3d3l	P18054	ALOX12
1161	ellagic acid	2d9h	Q9BU19	ZNF692
1162	ellagic acid	1igr	P08069	IGF1R
1163	ellagic acid	1z68	Q12884	FAP
1164	ellagic acid	2pkt	O00151	PDLIM1
1165	ellagic acid	2h63	P53004	BLVRA
1166	ellagic acid	2cop	Q9BR61	ACBD6
1167	ellagic acid	1h2t	P52298	NCBP2
1168	ellagic acid	1egw	Q02078	MEF2A
1169	ellagic acid	3fmo	Q9UMR2	DDX19B
1170	ellagic acid	1ow1	Q96T58	SPEN
1171	ellagic acid	2e29	Q9BQ39	DDX50
1172	ellagic acid	2aff	P46013	MKI67
1173	ellagic acid	1imh	O94916	NFAT5
1174	ellagic acid	2f3i	P52434	POLR2H
1175	ellagic acid	3eay	Q9BQF6	SENP7
1176	ellagic acid	1wi3	Q9UPW6	SATB2
1177	ellagic acid	1cf4	Q07912	TNK2
1178	ellagic acid	1x66	Q01543	FLI1
1179	ellagic acid	2ayn	P54578	USP14
1180	ellagic acid	2jrj	Q96PM5	RCHY1
1181	ellagic acid	3c5k	Q9UBN7	HDAC6
1182	ellagic acid	3dxf	Q9HBL8	NMRAL1
1183	ellagic acid	1rkh	Q15648	MED1
1184	ellagic acid	1rx0	Q9UKU7	ACAD8
1185	ellagic acid	3enm	P52564	MAP2K6
1186	ellagic acid	3e7o	P45984	MAPK9

1187	ellagic acid	3eg9	Q15436	SEC23A
1188	ellagic acid	1whl	Q9NQC7	CYLD
1189	ellagic acid	3c10	Q8WUI4	HDAC7
1190	ellagic acid	2coa	Q9BZL6	PRKD2
1191	ellagic acid	3cun	P09012	SNRPA
1192	ellagic acid	2enp	Q9BSW7	SYT17
1193	ellagic acid	2ql6	Q9NWW6	NMRK1
1194	ellagic acid	1ya0	Q92540	SMG7
1195	ellagic acid	2qqh	P07357	C8A
1196	ellagic acid	1x7z	P12694	BCKDHA
1197	ellagic acid	1wku	Q08043	ACTN3
1198	ellagic acid	3bg0	P55735	SEC13
1199	ellagic acid	1inz	Q9Y6I3	EPN1
1200	ellagic acid	1ln3	Q9UKL6	PCTP
1201	ellagic acid	1fg9	P15260	IFNGR1
1202	ellagic acid	2pp4	Q06455	RUNX1T1
1203	ellagic acid	1x6a	P53671	LIMK2
1204	ellagic acid	2ysd	Q96QZ7	MAGI1
1205	ellagic acid	2vr2	Q14117	DPYS
1206	ellagic acid	1x65	O75534	CSDE1
1207	ellagic acid	1cok	O15350	TP73
1208	ellagic acid	3bky	P11836	MS4A1
1209	ellagic acid	2h08	P60891	PRPS1
1210	ellagic acid	1n8z	P04626	ERBB2
1211	ellagic acid	2cue	P26367	PAX6
1212	ellagic acid	2enj	Q04759	PRKCQ
1213	ellagic acid	3d9s	P55064	AQP5
1214	ellagic acid	3bsz	P02766	TTR
1215	ellagic acid	1fyv	Q15399	TLR1
1216	ellagic acid	1ljr	P0CG30	GSTT2B
1217	ellagic acid	2r2p	P54756	EPHA5
1218	ellagic acid	1wl4	Q9BWD1	ACAT2
1219	ellagic acid	2cpt	O75351	VPS4B
1220	ellagic acid	2r55	Q9NSY2	STARD5
1221	ellagic acid	2yuc	Q9BUZ4	TRAF4
1222	ellagic acid	1a6z	P61769	B2M
1223	ellagic acid	2dfd	P40926	MDH2
1224	ellagic acid	2d0j	Q9NPZ5	B3GAT2
1225	ellagic acid	2bug	P07900	HSP90AA1
1226	ellagic acid	1ivh	P26440	IVD
1227	ellagic acid	2c9o	Q9Y265	RUVBL1
1228	ellagic acid	1jv1	Q16222	UAP1
1229	ellagic acid	1p22	P35222	CTNNB1
1230	ellagic acid	3fy2	P29320	EPHA3
1231	ellagic acid	1bj1	P15692	VEGFA
1232	ellagic acid	2dmy	Q96SI9	STRBP
1233	ellagic acid	3bfx	O00338	SULT1C2
1234	ellagic acid	1nty	O75962	TRIO
1235	ellagic acid	1wf8	Q9ULJ8	PPP1R9A
1236	ellagic acid	2a8m	Q9H6P5	TASP1
1237	wogonin	2dba	Q9H3U1	UNC45A
1238	wogonin	2nz2	P00966	ASS1
1239	wogonin	2vre	Q13011	ECH1
1240	wogonin	2pkt	O00151	PDLIM1

1241	wogonin	2fy1	P0DJD3	RBMY1A1
1242	wogonin	1uw4	Q9BZI7	UPF3B
1243	wogonin	2vig	P16219	ACADS
1244	wogonin	1snl	Q02818	NUCB1
1245	wogonin	2cra	Q92826	HOXB13
1246	wogonin	1d6v	P01834	IGKC
1247	wogonin	2aug	Q14449	GRB14
1248	wogonin	1so0	Q96C23	GALM
1249	wogonin	2bjn	Q86SZ2	TRAPPC6B
1250	wogonin	2p0r	O14815	CAPN9
1251	wogonin	1wh0	O94966	USP19
1252	wogonin	1jfi	Q01658	DR1
1253	wogonin	1gsn	P00390	GSR
1254	wogonin	2dnk	Q9BZC1	CELF4
1255	wogonin	2cue	P26367	PAX6
1256	wogonin	3dpl	Q93034	CUL5
1257	wogonin	1b4f	P29323	EPHB2
1258	wogonin	2ija	P18440	NAT1
1259	wogonin	2enj	Q04759	PRKCQ
1260	wogonin	2eb1	Q9UPY3	DICER1
1261	wogonin	2fg5	Q13636	RAB31
1262	wogonin	2ejr	O60341	KDM1A
1263	wogonin	1x86	Q9NZN5	ARHGEF12
1264	wogonin	2kbq	Q9Y6N9	USH1C
1265	wogonin	2z7x	Q15399	TLR1
1266	wogonin	2coa	Q9BZL6	PRKD2
1267	wogonin	1lo1	O95718	ESRRB
1268	wogonin	2ppi	Q9H2C0	GAN
1269	wogonin	2raj	Q9Y5X1	SNX9
1270	wogonin	3ch6	P28845	HSD11B1
1271	wogonin	2fy4	P28329	CHAT
1272	wogonin	3b68	P10275	AR
1273	wogonin	3ezt	Q9BY41	HDAC8
1274	wogonin	2do1	P82979	SARNP
1275	wogonin	2vkq	Q9H0P0	NT5C3A
1276	wogonin	1jey	P12956	XRCC6
1277	wogonin	2ql6	Q9NWW6	NMRK1
1278	wogonin	1d5b	P01834	IGKC
1279	wogonin	3epy	Q8N6N7	ACBD7
1280	wogonin	3gtu	P28161	GSTM2
1281	wogonin	2da0	Q9ULH1	ASAP1
1282	wogonin	2nmp	P32929	CTH
1283	wogonin	1fcx	P13631	RARG
1284	wogonin	1h2t	P52298	NCBP2
1285	wogonin	2dmq	Q9NQ69	LHX9
1286	hemerocallisamines IV	1n8z	P04626	ERBB2
1287	hemerocallisamines IV	2cop	Q9BR61	ACBD6
1288	hemerocallisamines IV	2eon	Q9Y2L8	ZKSCAN5
1289	hemerocallisamines IV	1mje	P60896	SEM1
1290	hemerocallisamines IV	3eg9	Q15436	SEC23A
1291	hemerocallisamines IV	1z68	Q12884	FAP
1292	hemerocallisamines IV	2raj	Q9Y5X1	SNX9
1293	hemerocallisamines IV	1um7	Q92796	DLG3
1294	hemerocallisamines IV	1ry7	P05230	FGF1

1295	hemerocallisamines IV	2d9h	Q9BU19	ZNF692
1296	hemerocallisamines IV	3bsz	P02766	TTR
1297	hemerocallisamines IV	2ejr	O60341	KDM1A
1298	hemerocallisamines IV	1x5m	Q9HB71	CACYBP
1299	hemerocallisamines IV	3e0l	Q9Y2T3	GDA
1300	hemerocallisamines IV	1fg9	P15260	IFNGR1
1301	hemerocallisamines IV	2aff	P46013	MKI67
1302	hemerocallisamines IV	2f3i	P52434	POLR2H
1303	hemerocallisamines IV	3bg0	P55735	SEC13
1304	hemerocallisamines IV	1jv1	Q16222	UAP1
1305	hemerocallisamines IV	1ow1	Q96T58	SPEN
1306	hemerocallisamines IV	3e7o	P45984	MAPK9
1307	hemerocallisamines IV	1w6j	P48449	LSS
1308	hemerocallisamines IV	2jrj	Q96PM5	RCHY1
1309	hemerocallisamines IV	2ysd	Q96QZ7	MAGI1
1310	hemerocallisamines IV	3eay	Q9BQF6	SENP7
1311	hemerocallisamines IV	2cpt	O75351	VPS4B
1312	hemerocallisamines IV	2ig7	Q9Y259	CHKB
1313	hemerocallisamines IV	1lar	P10586	PTPRF
1314	hemerocallisamines IV	3ifb	P12104	FABP2
1315	hemerocallisamines IV	2enp	Q9BSW7	SYT17
1316	hemerocallisamines IV	2e29	Q9BQ39	DDX50
1317	hemerocallisamines IV	1wry	O75368	SH3BGRL
1318	hemerocallisamines V	1n8z	P04626	ERBB2
1319	hemerocallisamines V	1mje	P60896	SEM1
1320	hemerocallisamines V	1igr	P08069	IGF1R
1321	hemerocallisamines V	2eon	Q9Y2L8	ZKSCAN5
1322	hemerocallisamines V	3eg9	Q15436	SEC23A
1323	hemerocallisamines V	2d9h	Q9BU19	ZNF692
1324	hemerocallisamines V	2f3i	P52434	POLR2H
1325	hemerocallisamines V	1xdt	Q99075	HBEGF
1326	hemerocallisamines V	2c47	P78368	CSNK1G2
1327	hemerocallisamines V	1ow1	Q96T58	SPEN
1328	hemerocallisamines V	2vr2	Q14117	DPYS
1329	hemerocallisamines V	2jrj	Q96PM5	RCHY1
1330	hemerocallisamines V	2aff	P46013	MKI67
1331	hemerocallisamines V	1x5m	Q9HB71	CACYBP
1332	hemerocallisamines V	2v62	Q86Y07	VRK2
1333	hemerocallisamines V	2h08	P60891	PRPS1
1334	hemerocallisamines V	3e7o	P45984	MAPK9
1335	hemerocallisamines V	2ysd	Q96QZ7	MAGI1
1336	hemerocallisamines V	3eay	Q9BQF6	SENP7
1337	hemerocallisamines V	3ifb	P12104	FABP2
1338	hemerocallisamines V	1ntg	P54577	YARS1
1339	hemerocallisamines V	2vpi	P49915	GMPS
1340	hemerocallisamines V	2e29	Q9BQ39	DDX50
1341	hemerocallisamines V	2enp	Q9BSW7	SYT17
1342	hemerocallisamines VI	3fmo	Q9UMR2	DDX19B
1343	hemerocallisamines VI	2enj	Q04759	PRKCQ
1344	hemerocallisamines VI	2cop	Q9BR61	ACBD6
1345	hemerocallisamines VI	2r4v	O15247	CLIC2
1346	hemerocallisamines VI	2f3i	P52434	POLR2H
1347	hemerocallisamines VI	1d6v	P01834	IGKC
1348	hemerocallisamines VI	1bj1	P15692	VEGFA

1349	hemerocallisamines VI	2h32	P12018	VPREB1
1350	hemerocallisamines VI	1rx0	Q9UKU7	ACAD8
1351	hemerocallisamines VI	2pxx	P0DPD7	EEF1AKMT4
1352	hemerocallisamines VI	1ivh	P26440	IVD
1353	hemerocallisamines VI	3ifb	P12104	FABP2
1354	hemerocallisamines VI	2r55	Q9NSY2	STARD5
1355	hemerocallisamines VI	1uit	Q8TDM6	DLG5
1356	hemerocallisamines VI	2ann	P51513	NOVA1
1357	hemerocallisamines VI	2ig7	Q9Y259	CHKB
1358	hemerocallisamines VI	2bug	P07900	HSP90AA1
1359	hemerocallisamines VI	1k5g	P43487	RANBP1
1360	hemerocallisamines VI	3bkb	P07332	FES
1361	hemerocallisamines VII	3fmo	Q9UMR2	DDX19B
1362	hemerocallisamines VII	1sy6	P07766	CD3E
1363	hemerocallisamines VII	2dkx	O95238	SPDEF
1364	hemerocallisamines VII	1z68	Q12884	FAP
1365	hemerocallisamines VII	2eon	Q9Y2L8	ZKSCAN5
1366	hemerocallisamines VII	1d6v	P01834	IGKC
1367	hemerocallisamines VII	2f57	Q9P286	PAK5
1368	hemerocallisamines VII	1m9i	P08133	ANXA6
1369	hemerocallisamines VII	1z0j	Q9H1K0	RBSN
1370	hemerocallisamines VII	2in6	P30291	WEE1
1371	hemerocallisamines VII	1j88	P12319	FCER1A
1372	hemerocallisamines VII	1p22	P35222	CTNNB1
1373	hemerocallisamines VII	2cob	Q96JN0	LCOR
1374	hemerocallisamines VII	2csw	O76064	RNF8
1375	hemerocallisamines VII	1b4f	P29323	EPHB2
1376	hemerocallisamines VII	2fh1	P06396	GSN
1377	hemerocallisamines VII	1w6j	P48449	LSS
1378	hemerocallisamines VII	3bsz	P02766	TTR
1379	hemerocallisamines VII	1wry	O75368	SH3BGRL
1380	hemerocallisamines VII	2eje	P78347	GTF2I
1381	hemerocallisamines VII	2ig7	Q9Y259	CHKB
1382	hemerocallisamines VII	2atx	P17081	RHOQ
1383	hemerocallisamines VII	1lo1	O95718	ESRRB
1384	hemerocallisamines VII	3e0l	Q9Y2T3	GDA
1385	hemerocallisamines VII	2e1o	Q03014	HHEX
1386	hemerocallisamines VII	2c0o	P08631	HCK
1387	hemerocallisamines VII	1ya0	Q92540	SMG7
1388	hemerocallisamines VII	2dmy	Q96SI9	STRBP
1389	hemerocallisamines VII	2bu7	Q15119	PDK2
1390	hemerocallisamines VII	1bj1	P15692	VEGFA
1391	hemerocallisamines VII	2enk	Q6PML9	SLC30A9
1392	hemerocallisamines VII	2hak	Q9P0L2	MARK1
1393	fulvanine D	2dkx	O95238	SPDEF
1394	fulvanine D	3fmo	Q9UMR2	DDX19B
1395	fulvanine D	1mje	P60896	SEM1
1396	fulvanine D	2eon	Q9Y2L8	ZKSCAN5
1397	fulvanine D	1d6v	P01834	IGKC
1398	fulvanine D	3g3n	Q13946	PDE7A
1399	fulvanine D	1wgm	Q14139	UBE4A
1400	fulvanine D	2raj	Q9Y5X1	SNX9
1401	fulvanine D	1z68	Q12884	FAP
1402	fulvanine D	2in6	P30291	WEE1

1403	fulvanine D	1aoa	P13797	PLS3
1404	fulvanine D	1k0n	O00299	CLIC1
1405	fulvanine D	2z7x	Q15399	TLR1
1406	fulvanine D	1x5m	Q9HB71	CACYBP
1407	fulvanine D	3bsz	P02766	TTR
1408	fulvanine D	3bg0	P55735	SEC13
1409	fulvanine D	2cob	Q96JN0	LCOR
1410	fulvanine D	3e0l	Q9Y2T3	GDA
1411	fulvanine D	2r2p	P54756	EPHA5
1412	fulvanine D	2ig7	Q9Y259	CHKB
1413	fulvanine D	1wuu	P51570	GALK1
1414	fulvanine D	1ivh	P26440	IVD
1415	fulvanine D	1w6j	P48449	LSS
1416	fulvanine D	2v62	Q86Y07	VRK2
1417	fulvanine D	2ysd	Q96QZ7	MAGI1
1418	fulvanine D	1igr	P08069	IGF1R
1419	fulvanine D	1vfc	Q15554	TERF2
1420	fulvanine D	2nzt	P52789	HK2
1421	2'-deoxyadenosine	2rdw	Q9UJM8	HAO1
1422	2'-deoxyadenosine	3bo5	Q53H47	SETMAR
1423	2'-deoxyadenosine	1t0j	Q13936	CACNA1C
1424	2'-deoxyadenosine	1kv3	P21980	TGM2
1425	2'-deoxyadenosine	1ry7	P05230	FGF1
1426	2'-deoxyadenosine	3e0g	P42702	LIFR
1427	2'-deoxyadenosine	2j7q	P0CG48	UBC
1428	2'-deoxyadenosine	2cmw	Q9HCP0	CSNK1G1
1429	2'-deoxyadenosine	2i75	P29074	PTPN4
1430	hemerocallisamine I	2b3y	P21399	ACO1
1431	hemerocallisamine I	1soj	Q13370	PDE3B
1432	hemerocallisamine I	2cop	Q9BR61	ACBD6
1433	hemerocallisamine I	1ds6	P15153	RAC2
1434	hemerocallisamine I	2raj	Q9Y5X1	SNX9
1435	hemerocallisamine I	1d6v	P01834	IGKC
1436	hemerocallisamine I	1exv	P06737	PYGL
1437	hemerocallisamine I	1n2l	P15289	ARSA
1438	hemerocallisamine I	3exg	P08559	PDHA1
1439	hemerocallisamine I	2h60	P51532	SMARCA4
1440	hemerocallisamine I	2hzp	Q16719	KYNU
1441	hemerocallisamine I	1z68	Q12884	FAP
1442	hemerocallisamine I	3bo5	Q53H47	SETMAR
1443	hemerocallisamine I	2ecv	Q9C035	TRIM5
1444	hemerocallisamine I	3bsq	P49862	KLK7
1445	hemerocallisamine I	1z0j	Q9H1K0	RBSN
1446	hemerocallisamine I	2f57	Q9P286	PAK5
1447	hemerocallisamine I	1k78	Q02548	PAX5
1448	hemerocallisamine I	2h32	P12018	VPREB1
1449	hemerocallisamine I	2c4j	P28161	GSTM2
1450	hemerocallisamine I	3eh2	P53992	SEC24C
1451	hemerocallisamine I	1ivh	P26440	IVD
1452	hemerocallisamine I	2v62	Q86Y07	VRK2
1453	hemerocallisamine I	3e0l	Q9Y2T3	GDA
1454	hemerocallisamine I	1xu8	P36952	SERPINB5
1455	hemerocallisamine I	2jx3	P35659	DEK
1456	hemerocallisamine I	1olz	Q92854	SEMA4D

1457	hemerocallisamine I	2ak4	P01889	HLA-B
1458	hemerocallisamine I	1w98	P24864	CCNE1
1459	hemerocallisamine I	1fg9	P15260	IFNGR1
1460	hemerocallisamine I	3g1n	Q7Z6Z7	HUWE1
1461	hemerocallisamine I	1x5m	Q9HB71	CACYBP
1462	hemerocallisamine I	2ysd	Q96QZ7	MAGI1
1463	hemerocallisamine I	2p39	Q9GZV9	FGF23
1464	hemerocallisamine I	2fg5	Q13636	RAB31
1465	hemerocallisamine I	2dmz	Q8NI35	PATJ
1466	hemerocallisamine I	2cpt	O75351	VPS4B
1467	hemerocallisamine I	1wuu	P51570	GALK1
1468	hemerocallisamine I	3bg0	P55735	SEC13
1469	hemerocallisamine I	1w6j	P48449	LSS
1470	hemerocallisamine I	2z7x	Q15399	TLR1
1471	hemerocallisamine I	3bsz	P02766	TTR
1472	hemerocallisamine I	1xa6	P52757	CHN2
1473	hemerocallisamine I	1w60	P12004	PCNA
1474	hemerocallisamine I	2j7q	P0CG48	UBC
1475	hemerocallisamine I	1l9x	Q92820	GGH
1476	hemerocallisamine I	1b4f	P29323	EPHB2
1477	hemerocallisamine I	1xap	P10826	RARB
1478	hemerocallisamine I	1fe8	P04275	VWF
1479	hemerocallisamine I	1p22	P35222	CTNNB1
1480	hemerocallisamine I	3dl2	Q8IX04	UEVLD
1481	hemerocallisamine I	2csw	O76064	RNF8
1482	hemerocallisamine I	2vr2	Q14117	DPYS
1483	hemerocallisamine I	1aii	P12429	ANXA3
1484	hemerocallisamine I	3bkb	P07332	FES
1485	hemerocallisamine I	1x47	Q8WYQ5	DGCR8
1486	hemerocallisamine I	2vkx	P13591	NCAM1
1487	hemerocallisamine I	2e29	Q9BQ39	DDX50
1488	hemerocallisamine I	2dkm	Q9P218	COL20A1
1489	hemerocallisamine I	3epz	P26358	DNMT1
1490	hemerocallisamine I	1pkx	P31939	ATIC
1491	hemerocallisamine I	1fsu	P15848	ARSB
1492	hemerocallisamine I	1vyh	P68402	PAFAH1B2
1493	hemerocallisamine I	1lo1	O95718	ESRRB
1494	hemerocallisamine I	1uf0	O15075	DCLK1
1495	hemerocallisamine I	2avd	Q86VU5	COMTD1
1496	hemerocallisamine I	1szb	O00187	MASP2
1497	hemerocallisamine I	2fh1	P06396	GSN
1498	hemerocallisamine I	2e1o	Q03014	HHEX
1499	hemerocallisamine I	1x7z	P12694	BCKDHA
1500	hemerocallisamine I	2h8r	P35680	HNF1B
1501	hemerocallisamine I	2dnk	Q9BZC1	CELF4
1502	hemerocallisamine I	2emj	Q8NHY6	ZFP28
1503	hemerocallisamine I	2ad1	O75897	SULT1C4
1504	hemerocallisamine I	2f9d	O75533	SF3B1
1505	hemerocallisamine I	2dl8	O75044	SRGAP2
1506	hemerocallisamine I	1bj1	P15692	VEGFA
1507	hemerocallisamine I	2gow	O95164	UBL3
1508	hemerocallisamine I	2do1	P82979	SARNP
1509	hemerocallisamine II	2qfd	O95786	DDX58
1510	hemerocallisamine II	1qz2	Q02790	FKBP4

1511	hemerocallisamine II	2qq5	Q96LJ7	DHRS1
1512	hemerocallisamine II	1ujv	Q86UL8	MAGI2
1513	hemerocallisamine II	2ig7	Q9Y259	CHKB
1514	hemerocallisamine II	2vre	Q13011	ECH1
1515	hemerocallisamine II	1snl	Q02818	NUCB1
1516	hemerocallisamine II	2acx	P43250	GRK6
1517	hemerocallisamine II	3cww	P14735	IDE
1518	hemerocallisamine II	2ihc	O14867	BACH1
1519	hemerocallisamine II	2cog	P54687	BCAT1
1520	hemerocallisamine II	3fmo	Q9UMR2	DDX19B
1521	hemerocallisamine II	2rmg	Q96RP3	UCN2
1522	hemerocallisamine II	1b72	P40424	PBX1
1523	hemerocallisamine II	2ecd	P42684	ABL2
1524	hemerocallisamine II	1t84	P42768	WAS
1525	hemerocallisamine II	1ry7	P05230	FGF1
1526	hemerocallisamine II	2i7k	Q9NPI1	BRD7
1527	hemerocallisamine II	1a6z	P61769	B2M
1528	hemerocallisamine II	1xjv	Q9NUX5	POT1
1529	hemerocallisamine II	3d3l	P18054	ALOX12
1530	hemerocallisamine II	1nu9	P00734	F2
1531	hemerocallisamine II	1khu	Q15797	SMAD1
1532	hemerocallisamine II	1p9o	Q9HAB8	PPCS
1533	hemerocallisamine II	1qib	P08253	MMP2
1534	hemerocallisamine II	1so0	Q96C23	GALM
1535	hemerocallisamine II	1ggt	P00488	F13A1
1536	hemerocallisamine II	1o9k	P06400	RB1
1537	hemerocallisamine II	2cra	Q92826	HOXB13
1538	hemerocallisamine II	1d6v	P01834	IGKC
1539	hemerocallisamine II	1e51	P13716	ALAD
1540	hemerocallisamine II	2p0r	O14815	CAPN9
1541	hemerocallisamine II	3dlj	Q96KN2	CNDP1
1542	hemerocallisamine II	2eje	P78347	GTF2I
1543	hemerocallisamine II	1u1k	P09651	HNRNPA1
1544	hemerocallisamine II	1zag	P25311	AZGP1
1545	hemerocallisamine II	2vpk	Q9NPC7	MYNN
1546	hemerocallisamine II	3eg9	Q15436	SEC23A
1547	hemerocallisamine II	2zw3	P29033	GJB2
1548	hemerocallisamine II	3g2f	Q13873	BMPR2
1549	hemerocallisamine II	7ics	P06746	POLB
1550	hemerocallisamine II	2raj	Q9Y5X1	SNX9
1551	hemerocallisamine II	1wh0	O94966	USP19
1552	hemerocallisamine II	2bc9	P32455	GBP1
1553	hemerocallisamine II	2f3i	P52434	POLR2H
1554	hemerocallisamine II	2fy4	P28329	CHAT
1555	hemerocallisamine II	2w96	P11802	CDK4
1556	hemerocallisamine II	2ee4	Q13017	ARHGAP5
1557	hemerocallisamine II	2pom	Q15750	TAB1
1558	hemerocallisamine II	1jv1	Q16222	UAP1
1559	hemerocallisamine II	2enj	Q04759	PRKCQ
1560	hemerocallisamine II	1egw	Q02078	MEF2A
1561	hemerocallisamine II	1vyh	P68402	PAFAH1B2
1562	hemerocallisamine II	2qq8	Q9P2M4	TBC1D14
1563	hemerocallisamine II	2bxg	P02768	ALB
1564	hemerocallisamine II	1jrh	P15260	IFNGR1

1565	hemerocallisamine II	3ifb	P12104	FABP2
1566	hemerocallisamine II	2zme	Q86VN1	VPS36
1567	hemerocallisamine II	2aff	P46013	MKI67
1568	hemerocallisamine II	2nn6	Q06265	EXOSC9
1569	hemerocallisamine II	1d5b	P01834	IGKC
1570	hemerocallisamine II	1rgo	P47974	ZFP36L2
1571	hemerocallisamine II	2kbq	Q9Y6N9	USH1C
1572	hemerocallisamine II	2z5f	O43704	SULT1B1
1573	hemerocallisamine II	2c2h	P60763	RAC3
1574	hemerocallisamine II	2ija	P18440	NAT1
1575	hemerocallisamine II	1w98	P24864	CCNE1
1576	hemerocallisamine II	1ow1	Q96T58	SPEN
1577	hemerocallisamine II	2ys5	O43559	FRS3
1578	hemerocallisamine II	2coa	Q9BZL6	PRKD2
1579	hemerocallisamine II	2ozb	P55769	SNU13
1580	hemerocallisamine II	2arp	P08476	INHBA
1581	hemerocallisamine II	2f9d	O75533	SF3B1
1582	hemerocallisamine II	1jey	P12956	XRCC6
1583	hemerocallisamine II	3bky	P11836	MS4A1
1584	hemerocallisamine II	2d2z	Q9Y696	CLIC4
1585	hemerocallisamine II	2hye	Q16531	DDB1
1586	hemerocallisamine II	2nmp	P32929	CTH
1587	hemerocallisamine II	2e1o	Q03014	HHEX
1588	hemerocallisamine II	2cpt	O75351	VPS4B
1589	hemerocallisamine II	2e29	Q9BQ39	DDX50
1590	hemerocallisamine II	2ppi	Q9H2C0	GAN
1591	hemerocallisamine III	3fmo	Q9UMR2	DDX19B
1592	hemerocallisamine III	2cop	Q9BR61	ACBD6
1593	hemerocallisamine III	3bhd	Q9BU02	THTPA
1594	hemerocallisamine III	1igr	P08069	IGF1R
1595	hemerocallisamine III	2raj	Q9Y5X1	SNX9
1596	hemerocallisamine III	1d6v	P01834	IGKC
1597	hemerocallisamine III	2d9h	Q9BU19	ZNF692
1598	hemerocallisamine III	2vr2	Q14117	DPYS
1599	hemerocallisamine III	3e7o	P45984	MAPK9
1600	hemerocallisamine III	1jv1	Q16222	UAP1
1601	hemerocallisamine III	2ig7	Q9Y259	CHKB
1602	hemerocallisamine III	2f3i	P52434	POLR2H
1603	hemerocallisamine III	2r2p	P54756	EPHA5
1604	hemerocallisamine III	1nty	O75962	TRIO
1605	hemerocallisamine III	1fe8	P04275	VWF
1606	hemerocallisamine III	2aff	P46013	MKI67
1607	hemerocallisamine III	2a98	Q96DU7	ITPKC
1608	hemerocallisamine III	1x7z	P12694	BCKDHA
1609	hemerocallisamine III	2jrj	Q96PM5	RCHY1
1610	hemerocallisamine III	2ysd	Q96QZ7	MAGI1
1611	hemerocallisamine III	2i7t	Q9UKF6	CPSF3
1612	hemerocallisamine III	2nzt	P52789	HK2
1613	hemerocallisamine III	1ya0	Q92540	SMG7
1614	hemerocallisamine III	1ow1	Q96T58	SPEN
1615	hemerocallisamine III	1s9c	P51659	HSD17B4
1616	hemerocallisamine III	2enp	Q9BSW7	SYT17
1617	quercetin-3-O-rutinoside	4wlj	Q16773	KYAT1
1618	quercetin-3-O-rutinoside	2vig	P16219	ACADS

1619	quercetin-3-O-rutinoside	2e5o	Q15650	TRIP4
1620	quercetin-3-O-rutinoside	2a2d	Q01415	GALK2
1621	quercetin-3-O-rutinoside	2rli	O43819	SCO2
1622	quercetin-3-O-rutinoside	2nn6	Q06265	EXOSC9
1623	quercetin-3-O-rutinoside	2vr2	Q14117	DPYS
1624	quercetin-3-O-rutinoside	2jun	O15344	MID1
1625	quercetin-3-O-rutinoside	2atx	P17081	RHOQ
1626	quercetin-3-O-rutinoside	2csw	O76064	RNF8
1627	quercetin-3-O-rutinoside	1cf4	Q07912	TNK2
1628	quercetin-3-O-rutinoside	2coa	Q9BZL6	PRKD2
1629	quercetin-3-O-rutinoside	1wuu	P51570	GALK1
1630	quercetin-3-O-rutinoside	2dmz	Q8NI35	PATJ
1631	quercetin-3-O-rutinoside	1jv1	Q16222	UAP1
1632	quercetin-3-O-rutinoside	2pla	Q8N335	GPD1L
1633	quercetin-3-O-rutinoside	1lm7	P15924	DSP
1634	quercetin-3-O-rutinoside	1h4r	P35240	NF2
1635	quercetin-3-O-rutinoside	1w60	P12004	PCNA
1636	quercetin-3-O-rutinoside	1imh	O94916	NFAT5
1637	quercetin-3-O-rutinoside	2grp	P46060	RANGAP1
1638	quercetin-3-O-rutinoside	2z5f	O43704	SULT1B1
1639	quercetin-3-O-rutinoside	1rt9	P00491	PNP
1640	quercetin-3-O-rutinoside	1qib	P08253	MMP2
1641	quercetin-3-O-rutinoside	1nst	P52848	NDST1
1642	quercetin-3-O-rutinoside	2csh	O43298	ZBTB43
1643	quercetin-3-O-rutinoside	1so0	Q96C23	GALM
1644	quercetin-3-O-rutinoside	2qq5	Q96LJ7	DHRS1
1645	quercetin-3-O-rutinoside	2pph	Q99759	MAP3K3
1646	quercetin-3-O-rutinoside	3gls	Q9NTG7	SIRT3
1647	quercetin-3-O-rutinoside	2dlw	O60496	DOK2
1648	quercetin-3-O-rutinoside	2odq	P06681	C2
1649	quercetin-3-O-rutinoside	2nzt	P52789	HK2
1650	quercetin-3-O-rutinoside	2o06	P19623	SRM
1651	quercetin-3-O-rutinoside	2da7	O60315	ZEB2
1652	quercetin-3-O-rutinoside	2r37	P22352	GPX3
1653	quercetin-3-O-rutinoside	1wch	Q12923	PTPN13
1654	quercetin-3-O-rutinoside	1v16	P12694	BCKDHA
1655	quercetin-3-O-rutinoside	2vpi	P49915	GMPS
1656	quercetin-3-O-rutinoside	2i75	P29074	PTPN4
1657	quercetin-3-O-rutinoside	2r55	Q9NSY2	STARD5
1658	quercetin-3-O-rutinoside	1iau	P10144	GZMB
1659	quercetin-3-O-rutinoside	2aa7	P08235	NR3C2
1660	quercetin-3-O-rutinoside	2d86	Q9UKW4	VAV3
1661	quercetin-3-O-rutinoside	1lar	P10586	PTPRF
1662	quercetin-3-O-rutinoside	2ee4	Q13017	ARHGAP5
1663	quercetin-3-O-rutinoside	2iwg	P19474	TRIM21
1664	quercetin-3-O-rutinoside	1h2t	P52298	NCBP2
1665	quercetin-3-O-rutinoside	2cls	Q92730	RND1
1666	quercetin-3-O-rutinoside	1x6f	Q96JM2	ZNF462
1667	quercetin-3-O-rutinoside	2eec	O75369	FLNB
1668	quercetin-3-O-rutinoside	1xfd	P42658	DPP6
1669	quercetin-3-O-rutinoside	3cjo	P52732	KIF11
1670	quercetin-3-O-rutinoside	1y4j	Q8NBJ7	SUMF2
1671	quercetin-3-O-rutinoside	2bu7	Q15119	PDK2
1672	quercetin-3-O-rutinoside	2c0o	P08631	HCK

1673	quercetin-3-O-rutinoside	2r2j	Q9BS26	ERP44
1674	quercetin-3-O-rutinoside	1ya0	Q92540	SMG7
1675	quercetin-3-O-rutinoside	2jbp	P49137	MAPKAPK2
1676	quercetin-3-O-rutinoside	2a98	Q96DU7	ITPKC
1677	quercetin-3-O-rutinoside	3gdq	P34931	HSPA1L
1678	quercetin-3-O-rutinoside	2dfd	P40926	MDH2
1679	quercetin-3-O-rutinoside	2ovr	P63208	SKP1
1680	quercetin-3-O-rutinoside	2h8r	P35680	HNF1B
1681	quercetin-3-O-rutinoside	2bug	P07900	HSP90AA1
1682	quercetin-3-O-rutinoside	1cqt	P14859	POU2F1
1683	quercetin-3-O-rutinoside	1k4w	Q15788	NCOA1
1684	quercetin-3-O-rutinoside	2ji4	O60256	PRPSAP2
1685	quercetin-3-O-rutinoside	2cu7	Q5VVJ2	MYSM1
1686	quercetin-3-O-rutinoside	1pve	P54727	RAD23B
1687	quercetin-3-O-rutinoside	2emj	Q8NHY6	ZFP28
1688	quercetin-3-O-rutinoside	2fju	P63000	RAC1
1689	quercetin-3-O-rutinoside	1x6g	P42679	MATK
1690	quercetin-3-O-rutinoside	3ezt	Q9BY41	HDAC8
1691	quercetin-3-O-rutinoside	1y8o	Q15120	PDK3
1692	quercetin-3-O-rutinoside	1uit	Q8TDM6	DLG5
1693	quercetin-3-O-β-D-galactopyranoside	2ayn	P54578	USP14
1694	quercetin-3-O-β-D-galactopyranoside	1w6j	P48449	LSS
1695	quercetin-3-O-β-D-galactopyranoside	2coa	Q9BZL6	PRKD2
1696	quercetin-3-O-β-D-galactopyranoside	2csw	O76064	RNF8
1697	quercetin-3-O-β-D-galactopyranoside	2jun	O15344	MID1
1698	quercetin-3-O-β-D-galactopyranoside	2ak4	P01889	HLA-B
1699	quercetin-3-O-β-D-galactopyranoside	1jv1	Q16222	UAP1
1700	quercetin-3-O-β-D-galactopyranoside	1xa6	P52757	CHN2
1701	quercetin-3-O-β-D-galactopyranoside	1bj1	P15692	VEGFA
1702	quercetin-3-O-β-D-galactopyranoside	2dmz	Q8NI35	PATJ
1703	quercetin-3-O-β-D-galactopyranoside	3epz	P26358	DNMT1
1704	quercetin-3-O-β-D-galactopyranoside	1lm7	P15924	DSP
1705	quercetin-3-O-β-D-galactopyranoside	1ow1	Q96T58	SPEN
1706	quercetin-3-O-β-D-galactopyranoside	2ysd	Q96QZ7	MAGI1
1707	quercetin-3-O-β-D-galactopyranoside	1fe8	P04275	VWF
1708	quercetin-3-O-β-D-galactopyranoside	2w96	P11802	CDK4
1709	quercetin-3-O-β-D-galactopyranoside	1w60	P12004	PCNA
1710	quercetin-3-O-β-D-galactopyranoside	2vr2	Q14117	DPYS
1711	quercetin-3-O-β-D-galactopyranoside	2csh	O43298	ZBTB43
1712	quercetin-3-O-β-D-galactopyranoside	2e5o	Q15650	TRIP4
1713	quercetin-3-O-β-D-galactopyranoside	1x6a	P53671	LIMK2
1714	quercetin-3-O-β-D-galactopyranoside	3bg0	P55735	SEC13
1715	quercetin-3-O-β-D-galactopyranoside	2ee4	Q13017	ARHGAP5
1716	quercetin-3-O-β-D-galactopyranoside	2dlw	O60496	DOK2
1717	quercetin-3-O-β-D-galactopyranoside	1l9x	Q92820	GGH
1718	quercetin-3-O-β-D-galactopyranoside	2bc9	P32455	GBP1
1719	quercetin-3-O-β-D-galactopyranoside	2r55	Q9NSY2	STARD5
1720	quercetin-3-O-β-D-galactopyranoside	3bkb	P07332	FES
1721	quercetin-3-O-β-D-galactopyranoside	1p22	P35222	CTNNB1
1722	quercetin-3-O-β-D-galactopyranoside	3bg9	P11498	PC
1723	quercetin-3-O-β-D-galactopyranoside	1mzd	P49863	GZMK
1724	quercetin-3-O-β-D-galactopyranoside	2vkx	P13591	NCAM1
1725	quercetin-3-O-β-D-galactopyranoside	2o06	P19623	SRM
1726	quercetin-3-O-β-D-galactopyranoside	1w9c	O14980	XPO1

1727	quercetin-3-O-β-D-galactopyranoside	2iwg	P19474	TRIM21
1728	quercetin-3-O-β-D-galactopyranoside	2e61	Q9H0M4	ZCWPW1
1729	quercetin-3-O-β-D-galactopyranoside	1exv	P06737	PYGL
1730	quercetin-3-O-β-D-galactopyranoside	3fr5	P15090	FABP4
1731	quercetin-3-O-β-D-galactopyranoside	2yty	O75534	CSDE1
1732	quercetin-3-O-β-D-galactopyranoside	1pve	P54727	RAD23B
1733	quercetin-3-O-β-D-galactopyranoside	2c0o	P08631	HCK
1734	quercetin-3-O-β-D-galactopyranoside	1e9a	P23919	DTYMK
1735	quercetin-3-O-β-D-galactopyranoside	2emj	Q8NHY6	ZFP28
1736	quercetin-3-O-β-D-galactopyranoside	1mje	P60896	SEM1
1737	quercetin-3-O-β-D-galactopyranoside	2a98	Q96DU7	ITPKC
1738	quercetin-3-O-β-D-galactopyranoside	2yum	Q8IYH5	ZZZ3
1739	quercetin-3-O-β-D-galactopyranoside	2vz6	Q9UQM7	CAMK2A
1740	quercetin-3-O-β-D-galactopyranoside	2eec	O75369	FLNB
1741	quercetin-3-O-β-D-galactopyranoside	3dkm	Q9ULT8	HECTD1
1742	quercetin-3-O-β-D-galactopyranoside	2odq	P06681	C2
1743	quercetin-3-O-β-D-galactopyranoside	2vkq	Q9H0P0	NT5C3A
1744	quercetin-3-O-β-D-galactopyranoside	1ux6	P07996	THBS1
1745	quercetin-3-O-β-D-galactopyranoside	2vpi	P49915	GMPS
1746	quercetin-3-O-β-D-galactopyranoside	1fcg	P12318	FCGR2A
1747	quercetin-3-O-β-D-galactopyranoside	2bu7	Q15119	PDK2
1748	quercetin-3-O-β-D-galactopyranoside	2ahx	Q15303	ERBB4
1749	quercetin-3-O-β-D-galactopyranoside	2bsk	P62072	TIMM10
1750	quercetin-3-O-β-D-galactopyranoside	2i7n	Q8TE04	PANK1
1751	quercetin-3-O-β-D-galactopyranoside	1cqt	P14859	POU2F1
1752	quercetin-3-O-β-D-galactopyranoside	2ji4	O60256	PRPSAP2
1753	quercetin-3-O-β-D-galactopyranoside	3eay	Q9BQF6	SENP7
1754	quercetin-3-O-β-D-galactopyranoside	2pph	Q99759	MAP3K3
1755	quercetin-3-O-β-D-galactopyranoside	3d68	Q96IY4	CPB2
1756	quercetin-3-O-β-D-galactopyranoside	1x6f	Q96JM2	ZNF462
1757	quercetin-3-O-β-D-glucopyranoside	2grp	P46060	RANGAP1
1758	quercetin-3-O-β-D-glucopyranoside	2c9o	Q9Y265	RUVBL1
1759	quercetin-3-O-β-D-glucopyranoside	3epz	P26358	DNMT1
1760	quercetin-3-O-β-D-glucopyranoside	2coa	Q9BZL6	PRKD2
1761	quercetin-3-O-β-D-glucopyranoside	2ysd	Q96QZ7	MAGI1
1762	quercetin-3-O-β-D-glucopyranoside	2csw	O76064	RNF8
1763	quercetin-3-O-β-D-glucopyranoside	1jv1	Q16222	UAP1
1764	quercetin-3-O-β-D-glucopyranoside	1wry	O75368	SH3BGRL
1765	quercetin-3-O-β-D-glucopyranoside	2e5o	Q15650	TRIP4
1766	quercetin-3-O-β-D-glucopyranoside	1w6j	P48449	LSS
1767	quercetin-3-O-β-D-glucopyranoside	1l9x	Q92820	GGH
1768	quercetin-3-O-β-D-glucopyranoside	2fg5	Q13636	RAB31
1769	quercetin-3-O-β-D-glucopyranoside	1bj1	P15692	VEGFA
1770	quercetin-3-O-β-D-glucopyranoside	1ow1	Q96T58	SPEN
1771	quercetin-3-O-β-D-glucopyranoside	2h7v	P63000	RAC1
1772	quercetin-3-O-β-D-glucopyranoside	1xa6	P52757	CHN2
1773	quercetin-3-O-β-D-glucopyranoside	2kbq	Q9Y6N9	USH1C
1774	quercetin-3-O-β-D-glucopyranoside	2csh	O43298	ZBTB43
1775	quercetin-3-O-β-D-glucopyranoside	1w45	P13928	ANXA8
1776	quercetin-3-O-β-D-glucopyranoside	1p22	P35222	CTNNB1
1777	quercetin-3-O-β-D-glucopyranoside	3bg9	P11498	PC
1778	quercetin-3-O-β-D-glucopyranoside	2dlu	Q8NI35	PATJ
1779	quercetin-3-O-β-D-glucopyranoside	3bkb	P07332	FES
1780	quercetin-3-O-β-D-glucopyranoside	1w60	P12004	PCNA

1781	quercetin-3-O-β-D-glucopyranoside	2jun	O15344	MID1
1782	quercetin-3-O-β-D-glucopyranoside	1b4f	P29323	EPHB2
1783	quercetin-3-O-β-D-glucopyranoside	3bg0	P55735	SEC13
1784	quercetin-3-O-β-D-glucopyranoside	2acx	P43250	GRK6
1785	quercetin-3-O-β-D-glucopyranoside	2vkx	P13591	NCAM1
1786	quercetin-3-O-β-D-glucopyranoside	1x7z	P12694	BCKDHA
1787	quercetin-3-O-β-D-glucopyranoside	2ep8	O00541	PES1
1788	quercetin-3-O-β-D-glucopyranoside	2dlw	O60496	DOK2
1789	quercetin-3-O-β-D-glucopyranoside	2vpi	P49915	GMPS
1790	quercetin-3-O-β-D-glucopyranoside	2r55	Q9NSY2	STARD5
1791	quercetin-3-O-β-D-glucopyranoside	2nn6	Q06265	EXOSC9
1792	quercetin-3-O-β-D-glucopyranoside	2o06	P19623	SRM
1793	quercetin-3-O-β-D-glucopyranoside	1mje	P60896	SEM1
1794	quercetin-3-O-β-D-glucopyranoside	2da7	O60315	ZEB2
1795	quercetin-3-O-β-D-glucopyranoside	3d8e	O00213	APBB1
1796	quercetin-3-O-β-D-glucopyranoside	2iwg	P19474	TRIM21
1797	quercetin-3-O-β-D-glucopyranoside	2aa7	P08235	NR3C2
1798	quercetin-3-O-β-D-glucopyranoside	2ch9	O76096	CST7
1799	quercetin-3-O-β-D-glucopyranoside	1mzd	P49863	GZMK
1800	quercetin-3-O-β-D-glucopyranoside	2ee4	Q13017	ARHGAP5
1801	quercetin-3-O-β-D-glucopyranoside	1e9a	P23919	DTYMK
1802	quercetin-3-O-β-D-glucopyranoside	1pve	P54727	RAD23B
1803	quercetin-3-O-β-D-glucopyranoside	2eec	O75369	FLNB
1804	quercetin-3-O-β-D-glucopyranoside	2c0o	P08631	HCK
1805	quercetin-3-O-β-D-glucopyranoside	1s79	P05455	SSB
1806	quercetin-3-O-β-D-glucopyranoside	3eay	Q9BQF6	SENP7
1807	quercetin-3-O-β-D-glucopyranoside	2emj	Q8NHY6	ZFP28
1808	quercetin-3-O-β-D-glucopyranoside	2bzl	Q15678	PTPN14
1809	quercetin-3-O-β-D-glucopyranoside	2bxg	P02768	ALB
1810	quercetin-3-O-β-D-glucopyranoside	1fch	P50542	PEX5
1811	quercetin-3-O-β-D-glucopyranoside	2ve7	O14777	NDC80
1812	quercetin-3-O-β-D-glucopyranoside	1rpm	P28827	PTPRM
1813	quercetin-3-O-β-D-glucopyranoside	1x6f	Q96JM2	ZNF462
1814	quercetin-3-O-β-D-glucopyranoside	2yum	Q8IYH5	ZZZ3
1815	quercetin-3-O-β-D-glucopyranoside	2pph	Q99759	MAP3K3
1816	quercetin-3-O-β-D-glucopyranoside	1us1	Q16853	AOC3
1817	quercetin-3-O-β-D-glucopyranoside	3eg9	Q15436	SEC23A
1818	quercetin-3-O-β-D-glucopyranoside	2zot	Q9HCB6	SPON1
1819	quercetin-3-O-β-D-glucopyranoside	2odq	P06681	C2
1820	quercetin-3-O-β-D-glucopyranoside	3d68	Q96IY4	CPB2
1821	quercetin-3-O-β-D-glucopyranoside	3dax	P22680	CYP7A1
1822	quercetin-3-O-β-D-glucopyranoside	1jdn	P17342	NPR3
1823	quercetin-3-O-β-D-glucopyranoside	3cjo	P52732	KIF11
1824	quercetin-3-O-β-D-glucopyranoside	2ahx	Q15303	ERBB4
1825	quercetin-3-O-β-D-glucopyranoside	1exv	P06737	PYGL
1826	quercetin-3-O-β-D-glucopyranoside	2da0	Q9ULH1	ASAP1
1827	quercetin-3-O-β-D-glucopyranoside	2dbm	Q99962	SH3GL2
1828	quercetin-3-O-β-D-glucopyranoside	2bu7	Q15119	PDK2
1829	loliolide	2cfv	Q12913	PTPRJ
1830	loliolide	2i7k	Q9NPI1	BRD7
1831	loliolide	2cue	P26367	PAX6
1832	loliolide	1so0	Q96C23	GALM
1833	loliolide	2jwe	Q07157	TJP1
1834	loliolide	2dgu	O60506	SYNCRIP

1835	loliolide	1jv1	Q16222	UAP1
1836	loliolide	1zag	P25311	AZGP1
1837	loliolide	1wh0	O94966	USP19
1838	loliolide	1d6v	P01834	IGKC
1839	loliolide	3bky	P11836	MS4A1
1840	loliolide	1buv	P50281	MMP14
1841	loliolide	1n8z	P04626	ERBB2
1842	loliolide	2i7t	Q9UKF6	CPSF3
1843	loliolide	2ozb	P55769	SNU13
1844	loliolide	2h08	P60891	PRPS1
1845	loliolide	3dpl	Q93034	CUL5
1846	loliolide	2ejr	O60341	KDM1A
1847	loliolide	2raw	O15392	BIRC5
1848	loliolide	2cpt	O75351	VPS4B
1849	loliolide	2ysd	Q96QZ7	MAGI1
1850	loliolide	2f3i	P52434	POLR2H
1851	loliolide	2c0o	P08631	HCK
1852	loliolide	1wi5	Q14690	PDCD11
1853	loliolide	2hzp	Q16719	KYNU
1854	loliolide	2vwe	P49765	VEGFB
1855	loliolide	1wi3	Q9UPW6	SATB2
1856	loliolide	2eov	Q5JVG2	ZNF484
1857	loliolide	1ntg	P54577	YARS1
1858	loliolide	3c5k	Q9UBN7	HDAC6
1859	loliolide	2bsk	P62072	TIMM10
1860	loliolide	2ee4	Q13017	ARHGAP5
1861	isololiolide	2cfv	Q12913	PTPRJ
1862	isololiolide	3bhd	Q9BU02	THTPA
1863	isololiolide	2cue	P26367	PAX6
1864	isololiolide	3e0g	P42702	LIFR
1865	isololiolide	1buv	P50281	MMP14
1866	isololiolide	2qfd	O95786	DDX58
1867	isololiolide	2jwe	Q07157	TJP1
1868	isololiolide	1d6v	P01834	IGKC
1869	isololiolide	1k0n	O00299	CLIC1
1870	isololiolide	2dgu	O60506	SYNCRIP
1871	isololiolide	1wh0	O94966	USP19
1872	isololiolide	1aoa	P13797	PLS3
1873	isololiolide	1ln3	Q9UKL6	PCTP
1874	isololiolide	2in6	P30291	WEE1
1875	isololiolide	1zag	P25311	AZGP1
1876	isololiolide	3bky	P11836	MS4A1
1877	isololiolide	1n8z	P04626	ERBB2
1878	isololiolide	1x7z	P12694	BCKDHA
1879	isololiolide	3d3l	P18054	ALOX12
1880	isololiolide	2h08	P60891	PRPS1
1881	isololiolide	2vr2	Q14117	DPYS
1882	isololiolide	2a98	Q96DU7	ITPKC
1883	isololiolide	2ozb	P55769	SNU13
1884	isololiolide	2ejr	O60341	KDM1A
1885	isololiolide	1jv1	Q16222	UAP1
1886	isololiolide	2coa	Q9BZL6	PRKD2
1887	isololiolide	2i7t	Q9UKF6	CPSF3
1888	isololiolide	3dpl	Q93034	CUL5

1889	isololiolide	1nty	O75962	TRIO
1890	isololiolide	2f3i	P52434	POLR2H
1891	isololiolide	1ntg	P54577	YARS1
1892	isololiolide	2yty	O75534	CSDE1
1893	isololiolide	1rxt	P30419	NMT1
1894	isololiolide	1wi3	Q9UPW6	SATB2
1895	isololiolide	2fju	P63000	RAC1
1896	isololiolide	3fy2	P29320	EPHA3
1897	isololiolide	2bsk	P62072	TIMM10
1898	dehydrololiolide	2cfv	Q12913	PTPRJ
1899	dehydrololiolide	2i7k	Q9NPI1	BRD7
1900	dehydrololiolide	2jwe	Q07157	TJP1
1901	dehydrololiolide	1egw	Q02078	MEF2A
1902	dehydrololiolide	2dgu	O60506	SYNCRIP
1903	dehydrololiolide	1d6v	P01834	IGKC
1904	dehydrololiolide	1jv1	Q16222	UAP1
1905	dehydrololiolide	1zag	P25311	AZGP1
1906	dehydrololiolide	1wh0	O94966	USP19
1907	dehydrololiolide	1fg9	P15260	IFNGR1
1908	dehydrololiolide	2ozb	P55769	SNU13
1909	dehydrololiolide	3bky	P11836	MS4A1
1910	dehydrololiolide	2f3i	P52434	POLR2H
1911	dehydrololiolide	2ysd	Q96QZ7	MAGI1
1912	dehydrololiolide	1mj4	P51687	SUOX
1913	dehydrololiolide	1wi3	Q9UPW6	SATB2
1914	dehydrololiolide	2eov	Q5JVG2	ZNF484
1915	dehydrololiolide	2d86	Q9UKW4	VAV3
1916	dehydrololiolide	3c5k	Q9UBN7	HDAC6
1917	(+)-dehydrovomifoliol	2cfv	Q12913	PTPRJ
1918	(+)-dehydrovomifoliol	2i7k	Q9NPI1	BRD7
1919	(+)-dehydrovomifoliol	1buv	P50281	MMP14
1920	(+)-dehydrovomifoliol	2qfd	O95786	DDX58
1921	(+)-dehydrovomifoliol	2cue	P26367	PAX6
1922	(+)-dehydrovomifoliol	2jwe	Q07157	TJP1
1923	(+)-dehydrovomifoliol	2dgu	O60506	SYNCRIP
1924	(+)-dehydrovomifoliol	2in6	P30291	WEE1
1925	(+)-dehydrovomifoliol	1aoa	P13797	PLS3
1926	(+)-dehydrovomifoliol	2v62	Q86Y07	VRK2
1927	(+)-dehydrovomifoliol	1zag	P25311	AZGP1
1928	(+)-dehydrovomifoliol	3bm4	Q9UKK9	NUDT5
1929	(+)-dehydrovomifoliol	2h08	P60891	PRPS1
1930	(+)-dehydrovomifoliol	2ozb	P55769	SNU13
1931	(+)-dehydrovomifoliol	1rxt	P30419	NMT1
1932	(+)-dehydrovomifoliol	2f3i	P52434	POLR2H
1933	(+)-dehydrovomifoliol	2i7t	Q9UKF6	CPSF3
1934	(+)-dehydrovomifoliol	1x4v	Q8WV99	ZFAND2B
1935	(+)-dehydrovomifoliol	2ejr	O60341	KDM1A
1936	(+)-dehydrovomifoliol	2vr2	Q14117	DPYS
1937	(+)-dehydrovomifoliol	3enm	P52564	MAP2K6
1938	(+)-dehydrovomifoliol	3bsz	P02766	TTR
1939	(+)-dehydrovomifoliol	2h63	P53004	BLVRA
1940	(+)-dehydrovomifoliol	2cob	Q96JN0	LCOR
1941	(+)-dehydrovomifoliol	1tg6	Q16740	CLPP
1942	(+)-dehydrovomifoliol	1u6g	Q13616	CUL1

1943	(+)-dehydrovomifoliol	2ysd	Q96QZ7	MAGI1
1944	(+)-dehydrovomifoliol	1mj4	P51687	SUOX
1945	(+)-dehydrovomifoliol	2ble	P36959	GMPR
1946	(+)-dehydrovomifoliol	2cpt	O75351	VPS4B
1947	(+)-dehydrovomifoliol	3dpl	Q93034	CUL5
1948	(+)-dehydrovomifoliol	3cwz	P20340	RAB6A
1949	(+)-dehydrovomifoliol	3e7g	P35228	NOS2
1950	(+)-dehydrovomifoliol	1wi3	Q9UPW6	SATB2
1951	(+)-dehydrovomifoliol	2vpi	P49915	GMPS
1952	(+)-dehydrovomifoliol	2fvl	P17516	AKR1C4
1953	(+)-dehydrovomifoliol	2hak	Q9P0L2	MARK1
1954	(+)-dehydrovomifoliol	1wuu	P51570	GALK1
1955	(+)-dehydrovomifoliol	1bh9	Q15543	TAF13
1956	(S)-abscisic acid	3c6g	Q6VVX0	CYP2R1
1957	(S)-abscisic acid	2rhk	O95639	CPSF4
1958	(S)-abscisic acid	2obh	P41208	CETN2
1959	(S)-abscisic acid	2uxw	P49748	ACADVL
1960	(S)-abscisic acid	2cpd	Q9NQ94	A1CF
1961	(S)-abscisic acid	2ziw	Q96AY2	EME1
1962	(S)-abscisic acid	3d7d	Q04609	FOLH1
1963	(S)-abscisic acid	2wa0	P43358	MAGEA4
1964	(S)-abscisic acid	2ecd	P42684	ABL2
1965	(S)-abscisic acid	2de0	Q9BYC5	FUT8
1966	(S)-abscisic acid	2i7k	Q9NPI1	BRD7
1967	(S)-abscisic acid	3ecs	Q14232	EIF2B1
1968	(S)-abscisic acid	3hki	P25116	F2R
1969	(S)-abscisic acid	2pp4	Q06455	RUNX1T1
1970	(S)-abscisic acid	2qtz	Q9UBK8	MTRR
1971	(S)-abscisic acid	2ytr	Q96SE7	ZNF347
1972	(S)-abscisic acid	2rli	O43819	SCO2
1973	(S)-abscisic acid	1snl	Q02818	NUCB1
1974	(S)-abscisic acid	2qq5	Q96LJ7	DHRS1
1975	(S)-abscisic acid	1x65	O75534	CSDE1
1976	(S)-abscisic acid	2cmw	Q9HCP0	CSNK1G1
1977	(S)-abscisic acid	1wh0	O94966	USP19
1978	(S)-abscisic acid	2dgu	O60506	SYNCRIP
1979	(S)-abscisic acid	2cpt	O75351	VPS4B
1980	(S)-abscisic acid	2p54	Q07869	PPARA
1981	(S)-abscisic acid	2h08	P60891	PRPS1
1982	(S)-abscisic acid	2aug	Q14449	GRB14
1983	(S)-abscisic acid	3eap	Q6P4F7	ARHGAP11A
1984	(S)-abscisic acid	1j55	P25815	S100P
1985	(S)-abscisic acid	2f8n	O75367	MACROH2A1
1986	(S)-abscisic acid	2os6	Q9NZN5	ARHGEF12
1987	(S)-abscisic acid	1r8u	Q99967	CITED2
1988	(S)-abscisic acid	1ggt	P00488	F13A1
1989	(S)-abscisic acid	1iq3	Q8NFH8	REPS2
1990	(S)-abscisic acid	1uw4	Q9BZI7	UPF3B
1991	(S)-abscisic acid	2h63	P53004	BLVRA
1992	(S)-abscisic acid	1so0	Q96C23	GALM
1993	(S)-abscisic acid	1cok	O15350	TP73
1994	(S)-abscisic acid	2uxn	O60341	KDM1A
1995	(S)-abscisic acid	2jx3	P35659	DEK
1996	(S)-abscisic acid	1ow1	Q96T58	SPEN

1997	(S)-abscisic acid	3dpl	Q93034	CUL5
1998	(S)-abscisic acid	1lar	P10586	PTPRF
1999	(S)-abscisic acid	2ee4	Q13017	ARHGAP5
2000	(S)-abscisic acid	1zag	P25311	AZGP1
2001	(S)-abscisic acid	2vwe	P49765	VEGFB
2002	(S)-abscisic acid	3bky	P11836	MS4A1
2003	(S)-abscisic acid	1woj	P09543	CNP
2004	(S)-abscisic acid	2j91	P30566	ADSL
2005	(S)-abscisic acid	2nn6	Q06265	EXOSC9
2006	(S)-abscisic acid	2i7t	Q9UKF6	CPSF3
2007	(S)-abscisic acid	2w4o	Q16566	CAMK4
2008	(S)-abscisic acid	2zj4	Q06210	GFPT1
2009	(S)-abscisic acid	1p32	Q07021	C1QBP
2010	(S)-abscisic acid	3epy	Q8N6N7	ACBD7
2011	(S)-abscisic acid	1bh9	Q15543	TAF13
2012	(S)-abscisic acid	2a98	Q96DU7	ITPKC
2013	(S)-abscisic acid	2czy	Q13127	REST
2014	(S)-abscisic acid	2zme	Q86VN1	VPS36
2015	5, 6-epoxy-3-hydroxy-7-megastigmen-9-ene	2r4v	O15247	CLIC2
2016	5, 6-epoxy-3-hydroxy-7-megastigmen-9-ene	2qp4	Q06520	SULT2A1
2017	5, 6-epoxy-3-hydroxy-7-megastigmen-9-ene	3bhd	Q9BU02	THTPA
2018	5, 6-epoxy-3-hydroxy-7-megastigmen-9-ene	1wh0	O94966	USP19
2019	5, 6-epoxy-3-hydroxy-7-megastigmen-9-ene	3fmo	Q9UMR2	DDX19B
2020	5, 6-epoxy-3-hydroxy-7-megastigmen-9-ene	1jv1	Q16222	UAP1
2021	5, 6-epoxy-3-hydroxy-7-megastigmen-9-ene	1egw	Q02078	MEF2A
2022	5, 6-epoxy-3-hydroxy-7-megastigmen-9-ene	2ee4	Q13017	ARHGAP5
2023	5, 6-epoxy-3-hydroxy-7-megastigmen-9-ene	1wuu	P51570	GALK1
2024	5, 6-epoxy-3-hydroxy-7-megastigmen-9-ene	2ig7	Q9Y259	CHKB
2025	5, 6-epoxy-3-hydroxy-7-megastigmen-9-ene	3bkb	P07332	FES
2026	5, 6-epoxy-3-hydroxy-7-megastigmen-9-ene	1xjv	Q9NUX5	POT1
2027	5, 6-epoxy-3-hydroxy-7-megastigmen-9-ene	3d3l	P18054	ALOX12
2028	5, 6-epoxy-3-hydroxy-7-megastigmen-9-ene	1w98	P24864	CCNE1
2029	5, 6-epoxy-3-hydroxy-7-megastigmen-9-ene	1zag	P25311	AZGP1
2030	5, 6-epoxy-3-hydroxy-7-megastigmen-9-ene	2nn6	Q06265	EXOSC9
2031	5, 6-epoxy-3-hydroxy-7-megastigmen-9-ene	2nut	Q15436	SEC23A
2032	5, 6-epoxy-3-hydroxy-7-megastigmen-9-ene	2ayn	P54578	USP14
2033	5, 6-epoxy-3-hydroxy-7-megastigmen-9-ene	2v62	Q86Y07	VRK2
2034	5, 6-epoxy-3-hydroxy-7-megastigmen-9-ene	2ke4	Q15642	TRIP10
2035	5, 6-epoxy-3-hydroxy-7-megastigmen-9-ene	1z68	Q12884	FAP
2036	5, 6-epoxy-3-hydroxy-7-megastigmen-9-ene	2raj	Q9Y5X1	SNX9
2037	5, 6-epoxy-3-hydroxy-7-megastigmen-9-ene	1ya0	Q92540	SMG7
2038	5, 6-epoxy-3-hydroxy-7-megastigmen-9-ene	2a98	Q96DU7	ITPKC
2039	5, 6-epoxy-3-hydroxy-7-megastigmen-9-ene	1soj	Q13370	PDE3B
2040	5, 6-epoxy-3-hydroxy-7-megastigmen-9-ene	2ql6	Q9NWW6	NMRK1
2041	5, 6-epoxy-3-hydroxy-7-megastigmen-9-ene	1d6v	P01834	IGKC
2042	5, 6-epoxy-3-hydroxy-7-megastigmen-9-ene	2h08	P60891	PRPS1
2043	5, 6-epoxy-3-hydroxy-7-megastigmen-9-ene	2bsk	P62072	TIMM10
2044	5, 6-epoxy-3-hydroxy-7-megastigmen-9-ene	3fy2	P29320	EPHA3
2045	5, 6-epoxy-3-hydroxy-7-megastigmen-9-ene	1uym	P08238	HSP90AB1
2046	5, 6-epoxy-3-hydroxy-7-megastigmen-9-ene	1wi3	Q9UPW6	SATB2
2047	5, 6-epoxy-3-hydroxy-7-megastigmen-9-ene	2a8m	Q9H6P5	TASP1
2048	5, 6-epoxy-3-hydroxy-7-megastigmen-9-ene	2h00	Q86W50	METTL16
2049	5, 6-epoxy-3-hydroxy-7-megastigmen-9-ene	3dax	P22680	CYP7A1
2050	5, 6-epoxy-3-hydroxy-7-megastigmen-9-ene	3c5k	Q9UBN7	HDAC6

2051	5, 6-epoxy-3-hydroxy-7-megastigmen-9-ene	3bky	P11836	MS4A1
2052	5, 6-epoxy-3-hydroxy-7-megastigmen-9-ene	1ujv	Q86UL8	MAGI2
2053	5, 6-epoxy-3-hydroxy-7-megastigmen-9-ene	2cue	P26367	PAX6
2054	5, 6-epoxy-3-hydroxy-7-megastigmen-9-ene	1n8z	P04626	ERBB2
2055	5, 6-epoxy-3-hydroxy-7-megastigmen-9-ene	2o3q	P28907	CD38
2056	5, 6-epoxy-3-hydroxy-7-megastigmen-9-ene	1ef1	P26038	MSN
2057	5, 6-epoxy-3-hydroxy-7-megastigmen-9-ene	2dlu	Q8NI35	PATJ
2058	5, 6-epoxy-3-hydroxy-7-megastigmen-9-ene	2ozb	P55769	SNU13
2059	5, 6-epoxy-3-hydroxy-7-megastigmen-9-ene	2vr2	Q14117	DPYS
2060	5, 6-epoxy-3-hydroxy-7-megastigmen-9-ene	2i7t	Q9UKF6	CPSF3
2061	5, 6-epoxy-3-hydroxy-7-megastigmen-9-ene	2d9n	O95639	CPSF4
2062	5, 6-epoxy-3-hydroxy-7-megastigmen-9-ene	2bzl	Q15678	PTPN14
2063	5, 6-epoxy-3-hydroxy-7-megastigmen-9-ene	2ad1	O75897	SULT1C4
2064	5, 6-epoxy-3-hydroxy-7-megastigmen-9-ene	2i6l	Q16659	MAPK6
2065	5, 6-epoxy-3-hydroxy-7-megastigmen-9-ene	1n83	P35398	RORA
2066	5, 6-epoxy-3-hydroxy-7-megastigmen-9-ene	2f3i	P52434	POLR2H
2067	5, 6-epoxy-3-hydroxy-7-megastigmen-9-ene	2dlz	P52735	VAV2
2068	5, 6-epoxy-3-hydroxy-7-megastigmen-9-ene	2d0j	Q9NPZ5	B3GAT2
2069	5, 6-epoxy-3-hydroxy-7-megastigmen-9-ene	3d9s	P55064	AQP5
2070	5, 6-epoxy-3-hydroxy-7-megastigmen-9-ene	2ysd	Q96QZ7	MAGI1
2071	5, 6-epoxy-3-hydroxy-7-megastigmen-9-ene	7ics	P06746	POLB
2072	5, 6-epoxy-3-hydroxy-7-megastigmen-9-ene	1ntg	P54577	YARS1
2073	5, 6-epoxy-3-hydroxy-7-megastigmen-9-ene	1xap	P10826	RARB
2074	5, 6-epoxy-3-hydroxy-7-megastigmen-9-ene	1pkx	P31939	ATIC
2075	5, 6-epoxy-3-hydroxy-7-megastigmen-9-ene	1tg6	Q16740	CLPP
2076	5, 6-epoxy-3-hydroxy-7-megastigmen-9-ene	2khn	Q15811	ITSN1
2077	5, 6-epoxy-3-hydroxy-7-megastigmen-9-ene	1ch4	P68871	HBB
2078	5, 6-epoxy-3-hydroxy-7-megastigmen-9-ene	1fo3	Q9UKM7	MAN1B1
2079	5, 6-epoxy-3-hydroxy-7-megastigmen-9-ene	2yuc	Q9BUZ4	TRAF4
2080	5, 6-epoxy-3-hydroxy-7-megastigmen-9-ene	2jbp	P49137	MAPKAPK2
2081	5, 6-epoxy-3-hydroxy-7-megastigmen-9-ene	1m9i	P08133	ANXA6
2082	5, 6-epoxy-3-hydroxy-7-megastigmen-9-ene	2fju	P63000	RAC1
2083	5, 6-epoxy-3-hydroxy-7-megastigmen-9-ene	2e8o	Q9Y3Z3	SAMHD1
2084	(3S,5R)-butyl-3-hydroxy-2-oxopyrrolidine-5-carboxylate	4i2x	Q9P1W8	SIRPG
2085	(3S,5R)-butyl-3-hydroxy-2-oxopyrrolidine-5-carboxylate	1ujv	Q86UL8	MAGI2
2086	(3S,5R)-butyl-3-hydroxy-2-oxopyrrolidine-5-carboxylate	1n8z	P04626	ERBB2
2087	(3S,5R)-butyl-3-hydroxy-2-oxopyrrolidine-5-carboxylate	3fmo	Q9UMR2	DDX19B
2088	(3S,5R)-butyl-3-hydroxy-2-oxopyrrolidine-5-carboxylate	2nn6	Q06265	EXOSC9
2089	(3S,5R)-butyl-3-hydroxy-2-oxopyrrolidine-5-carboxylate	1ds6	P15153	RAC2
2090	(3S,5R)-butyl-3-hydroxy-2-oxopyrrolidine-5-carboxylate	1ry7	P05230	FGF1
2091	(3S,5R)-butyl-3-hydroxy-2-oxopyrrolidine-5-carboxylate	2da7	O60315	ZEB2
2092	(3S,5R)-butyl-3-hydroxy-2-oxopyrrolidine-5-carboxylate	1rx0	Q9UKU7	ACAD8
2093	(3S,5R)-butyl-3-hydroxy-2-oxopyrrolidine-5-carboxylate	1khu	Q15797	SMAD1
2094	(3S,5R)-butyl-3-hydroxy-2-oxopyrrolidine-5-carboxylate	3eg9	Q15436	SEC23A
2095	(3S,5R)-butyl-3-hydroxy-2-oxopyrrolidine-5-carboxylate	2h32	P12018	VPREB1
2096	(3S,5R)-butyl-3-hydroxy-2-oxopyrrolidine-5-carboxylate	1egw	Q02078	MEF2A
2097	(3S,5R)-butyl-3-hydroxy-2-oxopyrrolidine-5-carboxylate	1x66	Q01543	FLI1
2098	(3S,5R)-butyl-3-hydroxy-2-oxopyrrolidine-5-carboxylate	2ann	P51513	NOVA1
2099	(3S,5R)-butyl-3-hydroxy-2-oxopyrrolidine-5-carboxylate	1fqv	Q13309	SKP2
2100	(3S,5R)-butyl-3-hydroxy-2-oxopyrrolidine-5-carboxylate	1d5m	P01903	HLA-DRA
2101	(3S,5R)-butyl-3-hydroxy-2-oxopyrrolidine-5-carboxylate	3e7o	P45984	MAPK9
2102	(3S,5R)-butyl-3-hydroxy-2-oxopyrrolidine-5-carboxylate	2ayn	P54578	USP14
2103	(3S,5R)-butyl-3-hydroxy-2-oxopyrrolidine-5-carboxylate	1xjv	Q9NUX5	POT1
2104	(3S,5R)-butyl-3-hydroxy-2-oxopyrrolidine-5-carboxylate	1igr	P08069	IGF1R

2105	(3S,5R)-butyl-3-hydroxy-2-oxopyrrolidine-5-carboxylate	3g2f	Q13873	BMPR2
2106	(3S,5R)-butyl-3-hydroxy-2-oxopyrrolidine-5-carboxylate	2iwq	O75970	MPDZ
2107	(3S,5R)-butyl-3-hydroxy-2-oxopyrrolidine-5-carboxylate	2dip	Q8NEG5	ZSWIM2
2108	(3S,5R)-butyl-3-hydroxy-2-oxopyrrolidine-5-carboxylate	2p82	Q8WYN0	ATG4A
2109	(3S,5R)-butyl-3-hydroxy-2-oxopyrrolidine-5-carboxylate	1jpk	P06132	UROD
2110	(3S,5R)-butyl-3-hydroxy-2-oxopyrrolidine-5-carboxylate	2csh	O43298	ZBTB43
2111	(3S,5R)-butyl-3-hydroxy-2-oxopyrrolidine-5-carboxylate	1ow1	Q96T58	SPEN
2112	(3S,5R)-butyl-3-hydroxy-2-oxopyrrolidine-5-carboxylate	2bc9	P32455	GBP1
2113	(3S,5R)-butyl-3-hydroxy-2-oxopyrrolidine-5-carboxylate	1p22	P35222	CTNNB1
2114	(3S,5R)-butyl-3-hydroxy-2-oxopyrrolidine-5-carboxylate	2dmz	Q8NI35	PATJ
2115	(3S,5R)-butyl-3-hydroxy-2-oxopyrrolidine-5-carboxylate	3bky	P11836	MS4A1
2116	(3S,5R)-butyl-3-hydroxy-2-oxopyrrolidine-5-carboxylate	1x5m	Q9HB71	CACYBP
2117	(3S,5R)-butyl-3-hydroxy-2-oxopyrrolidine-5-carboxylate	2d9p	Q9H361	PABPC3
2118	(3S,5R)-butyl-3-hydroxy-2-oxopyrrolidine-5-carboxylate	2raj	Q9Y5X1	SNX9
2119	(3S,5R)-butyl-3-hydroxy-2-oxopyrrolidine-5-carboxylate	3bhy	O43293	DAPK3
2120	(3S,5R)-butyl-3-hydroxy-2-oxopyrrolidine-5-carboxylate	2f3i	P52434	POLR2H
2121	(3S,5R)-butyl-3-hydroxy-2-oxopyrrolidine-5-carboxylate	1lw3	Q13614	MTMR2
2122	(3S,5R)-butyl-3-hydroxy-2-oxopyrrolidine-5-carboxylate	1wry	O75368	SH3BGRL
2123	(3S,5R)-butyl-3-hydroxy-2-oxopyrrolidine-5-carboxylate	3bg1	P55735	SEC13
2124	(3S,5R)-butyl-3-hydroxy-2-oxopyrrolidine-5-carboxylate	2enj	Q04759	PRKCQ
2125	(3S,5R)-butyl-3-hydroxy-2-oxopyrrolidine-5-carboxylate	2aff	P46013	MKI67
2126	(3S,5R)-butyl-3-hydroxy-2-oxopyrrolidine-5-carboxylate	2enk	Q6PML9	SLC30A9
2127	(3S,5R)-butyl-3-hydroxy-2-oxopyrrolidine-5-carboxylate	1lo1	O95718	ESRRB
2128	(3S,5R)-butyl-3-hydroxy-2-oxopyrrolidine-5-carboxylate	2fg5	Q13636	RAB31
2129	(3S,5R)-butyl-3-hydroxy-2-oxopyrrolidine-5-carboxylate	2ig7	Q9Y259	CHKB
2130	(3S,5R)-butyl-3-hydroxy-2-oxopyrrolidine-5-carboxylate	1jv1	Q16222	UAP1
2131	(3S,5R)-butyl-3-hydroxy-2-oxopyrrolidine-5-carboxylate	2v62	Q86Y07	VRK2
2132	(3S,5R)-butyl-3-hydroxy-2-oxopyrrolidine-5-carboxylate	2jrj	Q96PM5	RCHY1
2133	(3S,5R)-butyl-3-hydroxy-2-oxopyrrolidine-5-carboxylate	2e1o	Q03014	HHEX
2134	(3S,5R)-butyl-3-hydroxy-2-oxopyrrolidine-5-carboxylate	2ysd	Q96QZ7	MAGI1
2135	(3S,5R)-butyl-3-hydroxy-2-oxopyrrolidine-5-carboxylate	2i6a	P55263	ADK
2136	(3S,5R)-butyl-3-hydroxy-2-oxopyrrolidine-5-carboxylate	1w6j	P48449	LSS
2137	(3S,5R)-butyl-3-hydroxy-2-oxopyrrolidine-5-carboxylate	1x7z	P12694	BCKDHA
2138	(3S,5R)-butyl-3-hydroxy-2-oxopyrrolidine-5-carboxylate	3e0l	Q9Y2T3	GDA
2139	(3S,5R)-butyl-3-hydroxy-2-oxopyrrolidine-5-carboxylate	2e29	Q9BQ39	DDX50
2140	(3S,5R)-butyl-3-hydroxy-2-oxopyrrolidine-5-carboxylate	2eje	P78347	GTF2I
2141	(3S,5R)-butyl-3-hydroxy-2-oxopyrrolidine-5-carboxylate	2iwg	P19474	TRIM21
2142	(3S,5R)-butyl-3-hydroxy-2-oxopyrrolidine-5-carboxylate	1fsu	P15848	ARSB
2143	(3S,5R)-butyl-3-hydroxy-2-oxopyrrolidine-5-carboxylate	2csw	O76064	RNF8
2144	(3S,5R)-butyl-3-hydroxy-2-oxopyrrolidine-5-carboxylate	2fh1	P06396	GSN
2145	(3S,5R)-butyl-3-hydroxy-2-oxopyrrolidine-5-carboxylate	1vyh	P68402	PAFAH1B2
2146	(3S,5R)-butyl-3-hydroxy-2-oxopyrrolidine-5-carboxylate	1fe8	P04275	VWF
2147	(3S,5R)-butyl-3-hydroxy-2-oxopyrrolidine-5-carboxylate	2vr2	Q14117	DPYS
2148	(3S,5R)-butyl-3-hydroxy-2-oxopyrrolidine-5-carboxylate	2coa	Q9BZL6	PRKD2
2149	(3S,5R)-butyl-3-hydroxy-2-oxopyrrolidine-5-carboxylate	2pla	Q8N335	GPD1L
2150	(3S,5R)-butyl-3-hydroxy-2-oxopyrrolidine-5-carboxylate	1aii	P12429	ANXA3
2151	(3S,5R)-butyl-3-hydroxy-2-oxopyrrolidine-5-carboxylate	3enm	P52564	MAP2K6
2152	(3S,5R)-butyl-3-hydroxy-2-oxopyrrolidine-5-carboxylate	2j7q	P0CG48	UBC
2153	(3S,5R)-butyl-3-hydroxy-2-oxopyrrolidine-5-carboxylate	3bum	O43597	SPRY2
2154	(-)-(1S,3S)-1-methyl-1,2,3,4-tetrahydro-β-carboline-3-carboxylic acid	1z9e	O75475	PSIP1
2155	(-)-(1S,3S)-1-methyl-1,2,3,4-tetrahydro-β-carboline-3-carboxylic acid	1ujv	Q86UL8	MAGI2
2156	(-)-(1S,3S)-1-methyl-1,2,3,4-tetrahydro-β-carboline-3-carboxylic acid	2ayn	P54578	USP14
2157	(-)-(1S,3S)-1-methyl-1,2,3,4-tetrahydro-β-carboline-3-carboxylic acid	1n8z	P04626	ERBB2
2158	(-)-(1S,3S)-1-methyl-1,2,3,4-tetrahydro-β-carboline-3-carboxylic acid	2eon	Q9Y2L8	ZKSCAN5

2159	(-)-(1S,3S)-1-methyl-1,2,3,4-tetrahydro-β-carboline-3-carboxylic acid	2okk	Q05329	GAD2
2160	(-)-(1S,3S)-1-methyl-1,2,3,4-tetrahydro-β-carboline-3-carboxylic acid	1s9i	P36507	MAP2K2
2161	(-)-(1S,3S)-1-methyl-1,2,3,4-tetrahydro-β-carboline-3-carboxylic acid	2pkt	O00151	PDLIM1
2162	(-)-(1S,3S)-1-methyl-1,2,3,4-tetrahydro-β-carboline-3-carboxylic acid	3dlj	Q96KN2	CNDP1
2163	(-)-(1S,3S)-1-methyl-1,2,3,4-tetrahydro-β-carboline-3-carboxylic acid	1ow1	Q96T58	SPEN
2164	(-)-(1S,3S)-1-methyl-1,2,3,4-tetrahydro-β-carboline-3-carboxylic acid	1c9b	Q00403	GTF2B
2165	(-)-(1S,3S)-1-methyl-1,2,3,4-tetrahydro-β-carboline-3-carboxylic acid	2acx	P43250	GRK6
2166	(-)-(1S,3S)-1-methyl-1,2,3,4-tetrahydro-β-carboline-3-carboxylic acid	1whl	Q9NQC7	CYLD
2167	(-)-(1S,3S)-1-methyl-1,2,3,4-tetrahydro-β-carboline-3-carboxylic acid	1gh7	P32927	CSF2RB
2168	(-)-(1S,3S)-1-methyl-1,2,3,4-tetrahydro-β-carboline-3-carboxylic acid	2qp4	Q06520	SULT2A1
2169	(-)-(1S,3S)-1-methyl-1,2,3,4-tetrahydro-β-carboline-3-carboxylic acid	2j91	P30566	ADSL
2170	(-)-(1S,3S)-1-methyl-1,2,3,4-tetrahydro-β-carboline-3-carboxylic acid	2nut	Q15436	SEC23A
2171	(-)-(1S,3S)-1-methyl-1,2,3,4-tetrahydro-β-carboline-3-carboxylic acid	2nnu	O60885	BRD4
2172	(-)-(1S,3S)-1-methyl-1,2,3,4-tetrahydro-β-carboline-3-carboxylic acid	1wgy	Q92565	RAPGEF5
2173	(-)-(1S,3S)-1-methyl-1,2,3,4-tetrahydro-β-carboline-3-carboxylic acid	1w98	P24864	CCNE1
2174	(-)-(1S,3S)-1-methyl-1,2,3,4-tetrahydro-β-carboline-3-carboxylic acid	2v0v	Q14995	NR1D2
2175	(-)-(1S,3S)-1-methyl-1,2,3,4-tetrahydro-β-carboline-3-carboxylic acid	1x68	Q5TD97	FHL5
2176	(-)-(1S,3S)-1-methyl-1,2,3,4-tetrahydro-β-carboline-3-carboxylic acid	2vpk	Q9NPC7	MYNN
2177	(-)-(1S,3S)-1-methyl-1,2,3,4-tetrahydro-β-carboline-3-carboxylic acid	2ejr	O60341	KDM1A
2178	(-)-(1S,3S)-1-methyl-1,2,3,4-tetrahydro-β-carboline-3-carboxylic acid	2d9p	Q9H361	PABPC3
2179	(-)-(1S,3S)-1-methyl-1,2,3,4-tetrahydro-β-carboline-3-carboxylic acid	1xjv	Q9NUX5	POT1
2180	(-)-(1S,3S)-1-methyl-1,2,3,4-tetrahydro-β-carboline-3-carboxylic acid	3d3l	P18054	ALOX12
2181	(-)-(1S,3S)-1-methyl-1,2,3,4-tetrahydro-β-carboline-3-carboxylic acid	2hzp	Q16719	KYNU
2182	(-)-(1S,3S)-1-methyl-1,2,3,4-tetrahydro-β-carboline-3-carboxylic acid	3bhy	O43293	DAPK3
2183	(-)-(1S,3S)-1-methyl-1,2,3,4-tetrahydro-β-carboline-3-carboxylic acid	1us1	Q16853	AOC3
2184	(-)-(1S,3S)-1-methyl-1,2,3,4-tetrahydro-β-carboline-3-carboxylic acid	1s31	P50607	TUB
2185	(-)-(1S,3S)-1-methyl-1,2,3,4-tetrahydro-β-carboline-3-carboxylic acid	2zw3	P29033	GJB2
2186	(-)-(1S,3S)-1-methyl-1,2,3,4-tetrahydro-β-carboline-3-carboxylic acid	1qzy	P50579	METAP2
2187	(-)-(1S,3S)-1-methyl-1,2,3,4-tetrahydro-β-carboline-3-carboxylic acid	1ug3	Q04637	EIF4G1
2188	(-)-(1S,3S)-1-methyl-1,2,3,4-tetrahydro-β-carboline-3-carboxylic acid	2pp4	Q06455	RUNX1T1
2189	(-)-(1S,3S)-1-methyl-1,2,3,4-tetrahydro-β-carboline-3-carboxylic acid	2c4j	P28161	GSTM2
2190	(-)-(1S,3S)-1-methyl-1,2,3,4-tetrahydro-β-carboline-3-carboxylic acid	1aoa	P13797	PLS3
2191	(-)-(1S,3S)-1-methyl-1,2,3,4-tetrahydro-β-carboline-3-carboxylic acid	3eay	Q9BQF6	SENP7
2192	(-)-(1S,3S)-1-methyl-1,2,3,4-tetrahydro-β-carboline-3-carboxylic acid	1ry7	P05230	FGF1
2193	(-)-(1S,3S)-1-methyl-1,2,3,4-tetrahydro-β-carboline-3-carboxylic acid	2cue	P26367	PAX6
2194	(-)-(1S,3S)-1-methyl-1,2,3,4-tetrahydro-β-carboline-3-carboxylic acid	2iwg	P19474	TRIM21
2195	(-)-(1S,3S)-1-methyl-1,2,3,4-tetrahydro-β-carboline-3-carboxylic acid	2eqp	Q9NP84	TNFRSF12A
2196	(-)-(1S,3S)-1-methyl-1,2,3,4-tetrahydro-β-carboline-3-carboxylic acid	1jey	P12956	XRCC6
2197	(-)-(1S,3S)-1-methyl-1,2,3,4-tetrahydro-β-carboline-3-carboxylic acid	1nfb	P12268	IMPDH2
2198	(-)-(1S,3S)-1-methyl-1,2,3,4-tetrahydro-β-carboline-3-carboxylic acid	2vwe	P49765	VEGFB
2199	(-)-(1S,3S)-1-methyl-1,2,3,4-tetrahydro-β-carboline-3-carboxylic acid	1xcr	Q9H0W9	C11orf54
2200	(-)-(1S,3S)-1-methyl-1,2,3,4-tetrahydro-β-carboline-3-carboxylic acid	1q33	Q9BW91	NUDT9
2201	(-)-(1S,3S)-1-methyl-1,2,3,4-tetrahydro-β-carboline-3-carboxylic acid	1cf4	Q07912	TNK2
2202	(-)-(1S,3S)-1-methyl-1,2,3,4-tetrahydro-β-carboline-3-carboxylic acid	1jmj	P05546	SERPIND1
2203	(-)-(1S,3S)-1-methyl-1,2,3,4-tetrahydro-β-carboline-3-carboxylic acid	2e29	Q9BQ39	DDX50
2204	(-)-(1S,3S)-1-methyl-1,2,3,4-tetrahydro-β-carboline-3-carboxylic acid	1fe8	P04275	VWF
2205	(-)-(1S,3S)-1-methyl-1,2,3,4-tetrahydro-β-carboline-3-carboxylic acid	2h08	P60891	PRPS1
2206	(-)-(1S,3S)-1-methyl-1,2,3,4-tetrahydro-β-carboline-3-carboxylic acid	1u1k	P09651	HNRNPA1
2207	(-)-(1S,3S)-1-methyl-1,2,3,4-tetrahydro-β-carboline-3-carboxylic acid	2bxr	P21397	MAOA
2208	(-)-(1S,3S)-1-methyl-1,2,3,4-tetrahydro-β-carboline-3-carboxylic acid	1a6z	P61769	B2M
2209	(-)-(1S,3S)-1-methyl-1,2,3,4-tetrahydro-β-carboline-3-carboxylic acid	2eje	P78347	GTF2I
2210	(-)-(1S,3S)-1-methyl-1,2,3,4-tetrahydro-β-carboline-3-carboxylic acid	3c5k	Q9UBN7	HDAC6
2211	(-)-(1S,3S)-1-methyl-1,2,3,4-tetrahydro-β-carboline-3-carboxylic acid	1l9x	Q92820	GGH
2212	(-)-(1S,3S)-1-methyl-1,2,3,4-tetrahydro-β-carboline-3-carboxylic acid	1rxt	P30419	NMT1

2213	(-)-(1S,3S)-1-methyl-1,2,3,4-tetrahydro-β-carboline-3-carboxylic acid	1si2	Q9UL18	AGO1
2214	(-)-(1S,3S)-1-methyl-1,2,3,4-tetrahydro-β-carboline-3-carboxylic acid	2kbq	Q9Y6N9	USH1C
2215	(-)-(1S,3S)-1-methyl-1,2,3,4-tetrahydro-β-carboline-3-carboxylic acid	3e7o	P45984	MAPK9
2216	(-)-(1S,3S)-1-methyl-1,2,3,4-tetrahydro-β-carboline-3-carboxylic acid	3bky	P11836	MS4A1
2217	(-)-(1S,3S)-1-methyl-1,2,3,4-tetrahydro-β-carboline-3-carboxylic acid	2vkq	Q9H0P0	NT5C3A
2218	(-)-(1S,3S)-1-methyl-1,2,3,4-tetrahydro-β-carboline-3-carboxylic acid	1wch	Q12923	PTPN13
2219	(-)-(1S,3S)-1-methyl-1,2,3,4-tetrahydro-β-carboline-3-carboxylic acid	2pph	Q99759	MAP3K3
2220	(-)-(1S,3S)-1-methyl-1,2,3,4-tetrahydro-β-carboline-3-carboxylic acid	2crc	Q9BYM8	RBCK1
2221	(-)-(1S,3S)-1-methyl-1,2,3,4-tetrahydro-β-carboline-3-carboxylic acid	7fab	P01825	IGHV4-59
2222	(-)-(1S,3S)-1-methyl-1,2,3,4-tetrahydro-β-carboline-3-carboxylic acid	2r2p	P54756	EPHA5
2223	(-)-(1S,3S)-1-methyl-1,2,3,4-tetrahydro-β-carboline-3-carboxylic acid	2jbp	P49137	MAPKAPK2
2224	(-)-(1S,3S)-1-methyl-1,2,3,4-tetrahydro-β-carboline-3-carboxylic acid	3dwb	P42892	ECE1
2225	(-)-(1S,3S)-1-methyl-1,2,3,4-tetrahydro-β-carboline-3-carboxylic acid	1wji	Q9H7E2	TDRD3
2226	(-)-(1S,3S)-1-methyl-1,2,3,4-tetrahydro-β-carboline-3-carboxylic acid	2vr2	Q14117	DPYS
2227	prunasin	2j2s	Q03164	KMT2A
2228	prunasin	1wym	P37802	TAGLN2
2229	prunasin	2b3y	P21399	ACO1
2230	prunasin	1ivh	P26440	IVD
2231	prunasin	1exv	P06737	PYGL
2232	prunasin	2raj	Q9Y5X1	SNX9
2233	prunasin	2bka	Q9BUP3	HTATIP2
2234	prunasin	2iwq	O75970	MPDZ
2235	prunasin	1uew	Q86UL8	MAGI2
2236	prunasin	1wxb	Q9H6S3	EPS8L2
2237	prunasin	1fqv	Q13309	SKP2
2238	prunasin	1k78	Q02548	PAX5
2239	prunasin	3g2f	Q13873	BMPR2
2240	prunasin	1d5m	P01903	HLA-DRA
2241	prunasin	2aii	Q8NBK3	SUMF1
2242	prunasin	2q5y	P52948	NUP98
2243	prunasin	2ch9	O76096	CST7
2244	prunasin	2ocb	Q9NP90	RAB9B
2245	prunasin	1wm5	P19878	NCF2
2246	prunasin	1eg3	P11532	DMD
2247	prunasin	1igr	P08069	IGF1R
2248	prunasin	2dmz	Q8NI35	PATJ
2249	prunasin	2bc9	P32455	GBP1
2250	prunasin	2fg5	Q13636	RAB31
2251	prunasin	1l9x	Q92820	GGH
2252	prunasin	2coo	P11182	DBT
2253	prunasin	3dkm	Q9ULT8	HECTD1
2254	prunasin	3bg1	P55735	SEC13
2255	prunasin	2c9o	Q9Y265	RUVBL1
2256	prunasin	2c2h	P60763	RAC3
2257	prunasin	2okk	Q05329	GAD2
2258	prunasin	2z7x	Q15399	TLR1
2259	prunasin	2wgh	P23921	RRM1
2260	prunasin	2eje	P78347	GTF2I
2261	prunasin	1fe8	P04275	VWF
2262	prunasin	1w45	P13928	ANXA8
2263	prunasin	3bkb	P07332	FES
2264	prunasin	1w60	P12004	PCNA
2265	prunasin	1p22	P35222	CTNNB1
2266	prunasin	1szb	O00187	MASP2

2267	prunasin	2enk	Q6PML9	SLC30A9
2268	prunasin	2j7q	P0CG48	UBC
2269	prunasin	1w98	P24864	CCNE1
2270	prunasin	2pla	Q8N335	GPD1L
2271	prunasin	1jv1	Q16222	UAP1
2272	prunasin	2jun	O15344	MID1
2273	prunasin	3d8e	O00213	APBB1
2274	prunasin	3enm	P52564	MAP2K6
2275	prunasin	2csw	O76064	RNF8
2276	prunasin	2enj	Q04759	PRKCQ
2277	prunasin	1w6j	P48449	LSS
2278	prunasin	2ysd	Q96QZ7	MAGI1
2279	prunasin	2aff	P46013	MKI67
2280	prunasin	1bj1	P15692	VEGFA
2281	prunasin	1x7z	P12694	BCKDHA
2282	prunasin	1wfg	Q9UQ26	RIMS2
2283	prunasin	3bky	P11836	MS4A1
2284	prunasin	2f3i	P52434	POLR2H
2285	prunasin	2edu	Q14807	KIF22
2286	prunasin	3e0l	Q9Y2T3	GDA
2287	prunasin	2ad1	O75897	SULT1C4
2288	prunasin	2fuc	Q92871	PMM1
2289	prunasin	3g1n	Q7Z6Z7	HUWE1
2290	prunasin	1nrg	Q9NVS9	PNPO
2291	prunasin	2nn6	Q06265	EXOSC9
2292	prunasin	1wry	O75368	SH3BGRL
2293	prunasin	2coz	Q5VT06	CEP350
2294	prunasin	1rt9	P00491	PNP
2295	prunasin	2cpt	O75351	VPS4B
2296	prunasin	3eg9	Q15436	SEC23A
2297	prunasin	1pve	P54727	RAD23B
2298	prunasin	1x6f	Q96JM2	ZNF462
2299	5-O-p-coumaroyl-1,5-quinide lactone	3dpl	Q93034	CUL5
2300	5-O-p-coumaroyl-1,5-quinide lactone	2eon	Q9Y2L8	ZKSCAN5
2301	5-O-p-coumaroyl-1,5-quinide lactone	2nz2	P00966	ASS1
2302	5-O-p-coumaroyl-1,5-quinide lactone	2dfd	P40926	MDH2
2303	5-O-p-coumaroyl-1,5-quinide lactone	2j2s	Q03164	KMT2A
2304	5-O-p-coumaroyl-1,5-quinide lactone	1wym	P37802	TAGLN2
2305	5-O-p-coumaroyl-1,5-quinide lactone	2hye	Q16531	DDB1
2306	5-O-p-coumaroyl-1,5-quinide lactone	2pfi	P51800	CLCNKA
2307	5-O-p-coumaroyl-1,5-quinide lactone	1x66	Q01543	FLI1
2308	5-O-p-coumaroyl-1,5-quinide lactone	1fxy	P00742	F10
2309	5-O-p-coumaroyl-1,5-quinide lactone	2q5y	P52948	NUP98
2310	5-O-p-coumaroyl-1,5-quinide lactone	1wm5	P19878	NCF2
2311	5-O-p-coumaroyl-1,5-quinide lactone	2b3y	P21399	ACO1
2312	5-O-p-coumaroyl-1,5-quinide lactone	2dk1	O75554	WBP4
2313	5-O-p-coumaroyl-1,5-quinide lactone	3fe1	P17066	HSPA6
2314	5-O-p-coumaroyl-1,5-quinide lactone	2gf9	O95716	RAB3D
2315	5-O-p-coumaroyl-1,5-quinide lactone	2aii	Q8NBK3	SUMF1
2316	5-O-p-coumaroyl-1,5-quinide lactone	1wgm	Q14139	UBE4A
2317	5-O-p-coumaroyl-1,5-quinide lactone	3eh1	O95487	SEC24B
2318	5-O-p-coumaroyl-1,5-quinide lactone	3g2f	Q13873	BMPR2
2319	5-O-p-coumaroyl-1,5-quinide lactone	2c9o	Q9Y265	RUVBL1
2320	5-O-p-coumaroyl-1,5-quinide lactone	2c95	P00568	AK1

2321	5-O-p-coumaroyl-1,5-quinide lactone	2nnj	P10632	CYP2C8
2322	5-O-p-coumaroyl-1,5-quinide lactone	2pla	Q8N335	GPD1L
2323	5-O-p-coumaroyl-1,5-quinide lactone	2wgh	P23921	RRM1
2324	5-O-p-coumaroyl-1,5-quinide lactone	3bkb	P07332	FES
2325	5-O-p-coumaroyl-1,5-quinide lactone	1pj4	P23368	ME2
2326	5-O-p-coumaroyl-1,5-quinide lactone	2kdf	P55036	PSMD4
2327	5-O-p-coumaroyl-1,5-quinide lactone	2p39	Q9GZV9	FGF23
2328	5-O-p-coumaroyl-1,5-quinide lactone	2ad1	O75897	SULT1C4
2329	5-O-p-coumaroyl-1,5-quinide lactone	3bsz	P02766	TTR
2330	5-O-p-coumaroyl-1,5-quinide lactone	1szb	O00187	MASP2
2331	5-O-p-coumaroyl-1,5-quinide lactone	3bfx	O00338	SULT1C2
2332	5-O-p-coumaroyl-1,5-quinide lactone	1hwl	P04035	HMGCR
2333	5-O-p-coumaroyl-1,5-quinide lactone	1w9c	O14980	XPO1
2334	5-O-p-coumaroyl-1,5-quinide lactone	2eje	P78347	GTF2I
2335	5-O-p-coumaroyl-1,5-quinide lactone	2dkz	Q9H706	GAREM1
2336	5-O-p-coumaroyl-1,5-quinide lactone	2o06	P19623	SRM
2337	5-O-p-coumaroyl-1,5-quinide lactone	1rt9	P00491	PNP
2338	5-O-p-coumaroyl-1,5-quinide lactone	1fe8	P04275	VWF
2339	5-O-p-coumaroyl-1,5-quinide lactone	3enm	P52564	MAP2K6
2340	5-O-p-coumaroyl-1,5-quinide lactone	1lo1	O95718	ESRRB
2341	5-O-p-coumaroyl-1,5-quinide lactone	1lm7	P15924	DSP
2342	5-O-p-coumaroyl-1,5-quinide lactone	1vyh	P68402	PAFAH1B2
2343	5-O-p-coumaroyl-1,5-quinide lactone	2ysd	Q96QZ7	MAGI1
2344	5-O-p-coumaroyl-1,5-quinide lactone	2i6a	P55263	ADK
2345	5-O-p-coumaroyl-1,5-quinide lactone	1q8k	P05198	EIF2S1
2346	5-O-p-coumaroyl-1,5-quinide lactone	1w60	P12004	PCNA
2347	5-O-p-coumaroyl-1,5-quinide lactone	1wi1	Q9ULU8	CADPS
2348	5-O-p-coumaroyl-1,5-quinide lactone	2e5o	Q15650	TRIP4
2349	5-O-p-coumaroyl-1,5-quinide lactone	1w98	P24864	CCNE1
2350	5-O-p-coumaroyl-1,5-quinide lactone	3bky	P11836	MS4A1
2351	5-O-p-coumaroyl-1,5-quinide lactone	2ew1	Q15771	RAB30
2352	5-O-p-coumaroyl-1,5-quinide lactone	1w6j	P48449	LSS
2353	5-O-p-coumaroyl-1,5-quinide lactone	2dkm	Q9P218	COL20A1
2354	5-O-p-coumaroyl-1,5-quinide lactone	1jv1	Q16222	UAP1
2355	5-O-p-coumaroyl-1,5-quinide lactone	2coz	Q5VT06	CEP350
2356	5-O-p-coumaroyl-1,5-quinide lactone	3bg0	P55735	SEC13
2357	5-O-p-coumaroyl-1,5-quinide lactone	1wi5	Q14690	PDCD11
2358	5-O-p-coumaroyl-1,5-quinide lactone	1x86	Q9NZN5	ARHGEF12
2359	5-O-p-coumaroyl-1,5-quinide lactone	2jun	O15344	MID1
2360	5-O-p-coumaroyl-1,5-quinide lactone	1zxn	P11388	TOP2A
2361	5-O-p-coumaroyl-1,5-quinide lactone	2z7x	Q15399	TLR1
2362	5-O-p-coumaroyl-1,5-quinide lactone	1x6f	Q96JM2	ZNF462
2363	5-O-p-coumaroyl-1,5-quinide lactone	1lpj	Q96R05	RBP7
2364	5-O-p-coumaroyl-1,5-quinide lactone	2da7	O60315	ZEB2
2365	5-O-p-coumaroyl-1,5-quinide lactone	2c0o	P08631	HCK
2366	5-O-p-coumaroyl-1,5-quinide lactone	2cmw	Q9HCP0	CSNK1G1
2367	5-O-p-coumaroyl-1,5-quinide lactone	3d4j	P53602	MVD
2368	5-O-p-coumaroyl-1,5-quinide lactone	2uzg	Q8TEY7	USP33
2369	5-O-p-coumaroyl-1,5-quinide lactone	2e9x	Q14691	GINS1
2370	5-O-p-coumaroyl-1,5-quinide lactone	1v16	P12694	BCKDHA
2371	5-O-p-coumaroyl-1,5-quinide lactone	1puf	P40424	PBX1
2372	5-O-p-coumaroyl-1,5-quinide lactone	1igr	P08069	IGF1R
2373	5-O-p-coumaroyl-1,5-quinide lactone	2dlu	Q8NI35	PATJ
2374	5-O-p-coumaroyl-1,5-quinide lactone	2eov	Q5JVG2	ZNF484

2375	5-O-p-coumaroyl-1,5-quinide lactone	2i75	P29074	PTPN4
2376	5-O-p-coumaroyl-1,5-quinide lactone	2dmy	Q96SI9	STRBP
2377	5-O-p-coumaroyl-1,5-quinide lactone	1inz	Q9Y6I3	EPN1
2378	5-O-p-coumaroyl-1,5-quinide lactone	3d3k	Q96F86	EDC3
2379	5-O-p-coumaroyl-1,5-quinide lactone	1k78	Q02548	PAX5
2380	5-O-p-coumaroylquinic acid butyl ester	3d3l	P18054	ALOX12
2381	5-O-p-coumaroylquinic acid butyl ester	1o9k	P06400	RB1
2382	5-O-p-coumaroylquinic acid butyl ester	2bc9	P32455	GBP1
2383	5-O-p-coumaroylquinic acid butyl ester	2cra	Q92826	HOXB13
2384	5-O-p-coumaroylquinic acid butyl ester	2q5e	O14595	CTDSP2
2385	5-O-p-coumaroylquinic acid butyl ester	1rx0	Q9UKU7	ACAD8
2386	5-O-p-coumaroylquinic acid butyl ester	1x65	O75534	CSDE1
2387	5-O-p-coumaroylquinic acid butyl ester	1xjv	Q9NUX5	POT1
2388	5-O-p-coumaroylquinic acid butyl ester	3g73	Q08050	FOXM1
2389	5-O-p-coumaroylquinic acid butyl ester	2fg5	Q13636	RAB31
2390	5-O-p-coumaroylquinic acid butyl ester	1qgk	P52292	KPNA2
2391	5-O-p-coumaroylquinic acid butyl ester	2nn6	Q06265	EXOSC9
2392	5-O-p-coumaroylquinic acid butyl ester	1w60	P12004	PCNA
2393	5-O-p-coumaroylquinic acid butyl ester	2enj	Q04759	PRKCQ
2394	5-O-p-coumaroylquinic acid butyl ester	1d5b	P01834	IGKC
2395	5-O-p-coumaroylquinic acid butyl ester	2da7	O60315	ZEB2
2396	5-O-p-coumaroylquinic acid butyl ester	3bkb	P07332	FES
2397	5-O-p-coumaroylquinic acid butyl ester	2i7t	Q9UKF6	CPSF3
2398	5-O-p-coumaroylquinic acid butyl ester	3e7o	P45984	MAPK9
2399	5-O-p-coumaroylquinic acid butyl ester	1mj4	P51687	SUOX
2400	5-O-p-coumaroylquinic acid butyl ester	1imh	O94916	NFAT5
2401	5-O-p-coumaroylquinic acid butyl ester	2c9o	Q9Y265	RUVBL1
2402	5-O-p-coumaroylquinic acid butyl ester	2gl8	P48443	RXRG
2403	5-O-p-coumaroylquinic acid butyl ester	2ep8	O00541	PES1
2404	5-O-p-coumaroylquinic acid butyl ester	2cke	Q9UIK4	DAPK2
2405	5-O-p-coumaroylquinic acid butyl ester	2kdf	P55036	PSMD4
2406	5-O-p-coumaroylquinic acid butyl ester	2vwe	P49765	VEGFB
2407	5-O-p-coumaroylquinic acid butyl ester	1qib	P08253	MMP2
2408	5-O-p-coumaroylquinic acid butyl ester	7ics	P06746	POLB
2409	5-O-p-coumaroylquinic acid butyl ester	2c2h	P60763	RAC3
2410	5-O-p-coumaroylquinic acid butyl ester	2vag	P49759	CLK1
2411	5-O-p-coumaroylquinic acid butyl ester	1ow1	Q96T58	SPEN
2412	5-O-p-coumaroylquinic acid butyl ester	2ppi	Q9H2C0	GAN
2413	5-O-p-coumaroylquinic acid butyl ester	1jv1	Q16222	UAP1
2414	5-O-p-coumaroylquinic acid butyl ester	2a98	Q96DU7	ITPKC
2415	5-O-p-coumaroylquinic acid butyl ester	1wi3	Q9UPW6	SATB2
2416	5-O-p-coumaroylquinic acid butyl ester	3c5k	Q9UBN7	HDAC6
2417	5-O-p-coumaroylquinic acid butyl ester	2h2u	Q8WVQ1	CANT1
2418	5-O-p-coumaroylquinic acid butyl ester	3dl2	Q8IX04	UEVLD
2419	5-O-p-coumaroylquinic acid butyl ester	2iwg	P19474	TRIM21
2420	5-O-p-coumaroylquinic acid butyl ester	1lar	P10586	PTPRF
2421	5-O-p-coumaroylquinic acid butyl ester	1r6u	P23381	WARS1
2422	5-O-p-coumaroylquinic acid butyl ester	2h5g	P54886	ALDH18A1
2423	5-O-p-coumaroylquinic acid butyl ester	2ee4	Q13017	ARHGAP5
2424	5-O-p-coumaroylquinic acid butyl ester	3eg9	Q15436	SEC23A
2425	5-O-p-coumaroylquinic acid butyl ester	2okk	Q05329	GAD2
2426	5-O-p-coumaroylquinic acid butyl ester	3dkm	Q9ULT8	HECTD1
2427	5-O-p-coumaroylquinic acid butyl ester	2aa7	P08235	NR3C2
2428	5-O-p-coumaroylquinic acid butyl ester	2ji4	O60256	PRPSAP2

2429	5-O-p-coumaroylquinic acid butyl ester	1pve	P54727	RAD23B
2430	5-O-p-coumaroylquinic acid butyl ester	2o06	P19623	SRM
2431	5-O-p-coumaroylquinic acid butyl ester	3d68	Q96IY4	CPB2
2432	5-O-p-coumaroylquinic acid butyl ester	3b68	P10275	AR
2433	5-O-p-coumaroylquinic acid butyl ester	1uym	P08238	HSP90AB1
2434	5-O-p-coumaroylquinic acid butyl ester	2qq5	Q96LJ7	DHRS1
2435	5-O-p-coumaroylquinic acid butyl ester	3dax	P22680	CYP7A1
2436	5-O-p-coumaroylquinic acid butyl ester	2qp4	Q06520	SULT2A1
2437	5-O-p-coumaroylquinic acid butyl ester	3eay	Q9BQF6	SENP7
2438	5-O-p-coumaroylquinic acid butyl ester	2bxr	P21397	MAOA
2439	5-O-p-coumaroylquinic acid butyl ester	2wa0	P43358	MAGEA4
2440	5-O-p-coumaroylquinic acid butyl ester	1lfg	P02788	LTF
2441	5-O-p-coumaroylquinic acid butyl ester	1spj	P06870	KLK1
2442	5-O-p-coumaroylquinic acid butyl ester	3brw	P47736	RAP1GAP
2443	5-O-p-coumaroylquinic acid butyl ester	2bsk	P62072	TIMM10
2444	5-O-p-coumaroylquinic acid butyl ester	2g6b	Q9ULW5	RAB26
2445	5-O-p-coumaroylquinic acid butyl ester	2zj4	Q06210	GFPT1
2446	5-O-p-coumaroylquinic acid butyl ester	1xk0	P09601	HMOX1
2447	5-O-p-coumaroylquinic acid butyl ester	3bpt	Q6NVY1	HIBCH
2448	5-O-p-coumaroylquinic acid butyl ester	1iau	P10144	GZMB
2449	5-O-p-coumaroylquinic acid butyl ester	2c0o	P08631	HCK
2450	5-O-p-coumaroylquinic acid butyl ester	1y8o	Q15120	PDK3
2451	5-O-p-coumaroylquinic acid methyl ester	2fju	P63000	RAC1
2452	5-O-p-coumaroylquinic acid methyl ester	1eyb	Q93099	HGD
2453	5-O-p-coumaroylquinic acid methyl ester	2vx2	Q96DC8	ECHDC3
2454	5-O-p-coumaroylquinic acid methyl ester	2ivx	O60583	CCNT2
2455	5-O-p-coumaroylquinic acid methyl ester	3dpl	Q93034	CUL5
2456	5-O-p-coumaroylquinic acid methyl ester	1wym	P37802	TAGLN2
2457	5-O-p-coumaroylquinic acid methyl ester	3epz	P26358	DNMT1
2458	5-O-p-coumaroylquinic acid methyl ester	2eon	Q9Y2L8	ZKSCAN5
2459	5-O-p-coumaroylquinic acid methyl ester	2ziw	Q96AY2	EME1
2460	5-O-p-coumaroylquinic acid methyl ester	1x65	O75534	CSDE1
2461	5-O-p-coumaroylquinic acid methyl ester	1exv	P06737	PYGL
2462	5-O-p-coumaroylquinic acid methyl ester	1snl	Q02818	NUCB1
2463	5-O-p-coumaroylquinic acid methyl ester	2uyy	Q49A26	GLYR1
2464	5-O-p-coumaroylquinic acid methyl ester	2gf9	O95716	RAB3D
2465	5-O-p-coumaroylquinic acid methyl ester	2ayn	P54578	USP14
2466	5-O-p-coumaroylquinic acid methyl ester	2z14	Q5JST6	EFHC2
2467	5-O-p-coumaroylquinic acid methyl ester	1wm5	P19878	NCF2
2468	5-O-p-coumaroylquinic acid methyl ester	2q5e	O14595	CTDSP2
2469	5-O-p-coumaroylquinic acid methyl ester	1d6v	P01834	IGKC
2470	5-O-p-coumaroylquinic acid methyl ester	1o9k	P06400	RB1
2471	5-O-p-coumaroylquinic acid methyl ester	2b3y	P21399	ACO1
2472	5-O-p-coumaroylquinic acid methyl ester	1kw2	P02774	GC
2473	5-O-p-coumaroylquinic acid methyl ester	2q5y	P52948	NUP98
2474	5-O-p-coumaroylquinic acid methyl ester	1yb1	Q8NBQ5	HSD17B11
2475	5-O-p-coumaroylquinic acid methyl ester	2rdw	Q9UJM8	HAO1
2476	5-O-p-coumaroylquinic acid methyl ester	1gzq	P29016	CD1B
2477	5-O-p-coumaroylquinic acid methyl ester	2vpk	Q9NPC7	MYNN
2478	5-O-p-coumaroylquinic acid methyl ester	1qgk	P52292	KPNA2
2479	5-O-p-coumaroylquinic acid methyl ester	2da7	O60315	ZEB2
2480	5-O-p-coumaroylquinic acid methyl ester	2o06	P19623	SRM
2481	5-O-p-coumaroylquinic acid methyl ester	2kdf	P55036	PSMD4
2482	5-O-p-coumaroylquinic acid methyl ester	1imh	O94916	NFAT5

2483	5-O-p-coumaroylquinic acid methyl ester	2okk	Q05329	GAD2
2484	5-O-p-coumaroylquinic acid methyl ester	3eg9	Q15436	SEC23A
2485	5-O-p-coumaroylquinic acid methyl ester	2fxr	P20231	TPSB2
2486	5-O-p-coumaroylquinic acid methyl ester	1s9c	P51659	HSD17B4
2487	5-O-p-coumaroylquinic acid methyl ester	2gl8	P48443	RXRG
2488	5-O-p-coumaroylquinic acid methyl ester	1x7z	P12694	BCKDHA
2489	5-O-p-coumaroylquinic acid methyl ester	2aa7	P08235	NR3C2
2490	5-O-p-coumaroylquinic acid methyl ester	1zag	P25311	AZGP1
2491	5-O-p-coumaroylquinic acid methyl ester	2ija	P18440	NAT1
2492	5-O-p-coumaroylquinic acid methyl ester	1tz5	P01298	PPY
2493	5-O-p-coumaroylquinic acid methyl ester	2zu6	P60842	EIF4A1
2494	5-O-p-coumaroylquinic acid methyl ester	2pla	Q8N335	GPD1L
2495	5-O-p-coumaroylquinic acid methyl ester	2dkm	Q9P218	COL20A1
2496	5-O-p-coumaroylquinic acid methyl ester	1gqm	P80511	S100A12
2497	5-O-p-coumaroylquinic acid methyl ester	1iol	P14061	HSD17B1
2498	5-O-p-coumaroylquinic acid methyl ester	2ad1	O75897	SULT1C4
2499	5-O-p-coumaroylquinic acid methyl ester	2ejr	O60341	KDM1A
2500	5-O-p-coumaroylquinic acid methyl ester	3bg9	P11498	PC
2501	5-O-p-coumaroylquinic acid methyl ester	2iag	Q16647	PTGIS
2502	5-O-p-coumaroylquinic acid methyl ester	2c9o	Q9Y265	RUVBL1
2503	5-O-p-coumaroylquinic acid methyl ester	2kbq	Q9Y6N9	USH1C
2504	5-O-p-coumaroylquinic acid methyl ester	1jey	P12956	XRCC6
2505	5-O-p-coumaroylquinic acid methyl ester	1h9u	P28702	RXRB
2506	5-O-p-coumaroylquinic acid methyl ester	1fsu	P15848	ARSB
2507	5-O-p-coumaroylquinic acid methyl ester	1szb	O00187	MASP2
2508	5-O-p-coumaroylquinic acid methyl ester	3gcx	P01130	LDLR
2509	5-O-p-coumaroylquinic acid methyl ester	1lm7	P15924	DSP
2510	5-O-p-coumaroylquinic acid methyl ester	1jrh	P15260	IFNGR1
2511	5-O-p-coumaroylquinic acid methyl ester	1pkx	P31939	ATIC
2512	5-O-p-coumaroylquinic acid methyl ester	1x4v	Q8WV99	ZFAND2B
2513	5-O-p-coumaroylquinic acid methyl ester	1wf8	Q9ULJ8	PPP1R9A
2514	5-O-p-coumaroylquinic acid methyl ester	1wi1	Q9ULU8	CADPS
2515	5-O-p-coumaroylquinic acid methyl ester	1fe8	P04275	VWF
2516	5-O-p-coumaroylquinic acid methyl ester	2e5o	Q15650	TRIP4
2517	5-O-p-coumaroylquinic acid methyl ester	3bg1	P55735	SEC13
2518	5-O-p-coumaroylquinic acid methyl ester	2v62	Q86Y07	VRK2
2519	5-O-p-coumaroylquinic acid methyl ester	1qib	P08253	MMP2
2520	5-O-p-coumaroylquinic acid methyl ester	2a98	Q96DU7	ITPKC
2521	5-O-p-coumaroylquinic acid methyl ester	2ji4	O60256	PRPSAP2
2522	methyl 2,4-dihydroxy-6-(4-hydroxyphenethyl)-3-methylbenzoate	2qq5	Q96LJ7	DHRS1
2523	methyl 2,4-dihydroxy-6-(4-hydroxyphenethyl)-3-methylbenzoate	2dba	Q9H3U1	UNC45A
2524	methyl 2,4-dihydroxy-6-(4-hydroxyphenethyl)-3-methylbenzoate	1ggt	P00488	F13A1
2525	methyl 2,4-dihydroxy-6-(4-hydroxyphenethyl)-3-methylbenzoate	1eyb	Q93099	HGD
2526	methyl 2,4-dihydroxy-6-(4-hydroxyphenethyl)-3-methylbenzoate	2dhy	Q9NWM3	CUEDC1
2527	methyl 2,4-dihydroxy-6-(4-hydroxyphenethyl)-3-methylbenzoate	2vre	Q13011	ECH1
2528	methyl 2,4-dihydroxy-6-(4-hydroxyphenethyl)-3-methylbenzoate	1tfc	P62508	ESRRG
2529	methyl 2,4-dihydroxy-6-(4-hydroxyphenethyl)-3-methylbenzoate	3bhd	Q9BU02	THTPA
2530	methyl 2,4-dihydroxy-6-(4-hydroxyphenethyl)-3-methylbenzoate	2ayn	P54578	USP14
2531	methyl 2,4-dihydroxy-6-(4-hydroxyphenethyl)-3-methylbenzoate	1x65	O75534	CSDE1
2532	methyl 2,4-dihydroxy-6-(4-hydroxyphenethyl)-3-methylbenzoate	3d3l	P18054	ALOX12
2533	methyl 2,4-dihydroxy-6-(4-hydroxyphenethyl)-3-methylbenzoate	1dk8	O15169	AXIN1
2534	methyl 2,4-dihydroxy-6-(4-hydroxyphenethyl)-3-methylbenzoate	1snl	Q02818	NUCB1
2535	methyl 2,4-dihydroxy-6-(4-hydroxyphenethyl)-3-methylbenzoate	1gzq	P29016	CD1B
2536	methyl 2,4-dihydroxy-6-(4-hydroxyphenethyl)-3-methylbenzoate	1z0j	Q9H1K0	RBSN

2537	methyl 2,4-dihydroxy-6-(4-hydroxyphenethyl)-3-methylbenzoate	1so0	Q96C23	GALM
2538	methyl 2,4-dihydroxy-6-(4-hydroxyphenethyl)-3-methylbenzoate	1igr	P08069	IGF1R
2539	methyl 2,4-dihydroxy-6-(4-hydroxyphenethyl)-3-methylbenzoate	2vig	P16219	ACADS
2540	methyl 2,4-dihydroxy-6-(4-hydroxyphenethyl)-3-methylbenzoate	1uw4	Q9BZI7	UPF3B
2541	methyl 2,4-dihydroxy-6-(4-hydroxyphenethyl)-3-methylbenzoate	1cok	O15350	TP73
2542	methyl 2,4-dihydroxy-6-(4-hydroxyphenethyl)-3-methylbenzoate	2z6e	Q9Y4D1	DAAM1
2543	methyl 2,4-dihydroxy-6-(4-hydroxyphenethyl)-3-methylbenzoate	2h63	P53004	BLVRA
2544	methyl 2,4-dihydroxy-6-(4-hydroxyphenethyl)-3-methylbenzoate	1d5b	P01834	IGKC
2545	methyl 2,4-dihydroxy-6-(4-hydroxyphenethyl)-3-methylbenzoate	2coa	Q9BZL6	PRKD2
2546	methyl 2,4-dihydroxy-6-(4-hydroxyphenethyl)-3-methylbenzoate	2dmy	Q96SI9	STRBP
2547	methyl 2,4-dihydroxy-6-(4-hydroxyphenethyl)-3-methylbenzoate	1zag	P25311	AZGP1
2548	methyl 2,4-dihydroxy-6-(4-hydroxyphenethyl)-3-methylbenzoate	1inz	Q9Y6I3	EPN1
2549	methyl 2,4-dihydroxy-6-(4-hydroxyphenethyl)-3-methylbenzoate	2enj	Q04759	PRKCQ
2550	methyl 2,4-dihydroxy-6-(4-hydroxyphenethyl)-3-methylbenzoate	1ya0	Q92540	SMG7
2551	methyl 2,4-dihydroxy-6-(4-hydroxyphenethyl)-3-methylbenzoate	1r6u	P23381	WARS1
2552	methyl 2,4-dihydroxy-6-(4-hydroxyphenethyl)-3-methylbenzoate	2csw	O76064	RNF8
2553	methyl 2,4-dihydroxy-6-(4-hydroxyphenethyl)-3-methylbenzoate	3ifb	P12104	FABP2
2554	methyl 2,4-dihydroxy-6-(4-hydroxyphenethyl)-3-methylbenzoate	2fg5	Q13636	RAB31
2555	methyl 2,4-dihydroxy-6-(4-hydroxyphenethyl)-3-methylbenzoate	2bc9	P32455	GBP1
2556	methyl 2,4-dihydroxy-6-(4-hydroxyphenethyl)-3-methylbenzoate	2vr2	Q14117	DPYS
2557	methyl 2,4-dihydroxy-6-(4-hydroxyphenethyl)-3-methylbenzoate	2hzp	Q16719	KYNU
2558	methyl 2,4-dihydroxy-6-(4-hydroxyphenethyl)-3-methylbenzoate	1tg6	Q16740	CLPP
2559	methyl 2,4-dihydroxy-6-(4-hydroxyphenethyl)-3-methylbenzoate	3bg0	P55735	SEC13
2560	methyl 2,4-dihydroxy-6-(4-hydroxyphenethyl)-3-methylbenzoate	2raj	Q9Y5X1	SNX9
2561	methyl 2,4-dihydroxy-6-(4-hydroxyphenethyl)-3-methylbenzoate	3dl2	Q8IX04	UEVLD
2562	methyl 2,4-dihydroxy-6-(4-hydroxyphenethyl)-3-methylbenzoate	2z7x	Q15399	TLR1
2563	methyl 2,4-dihydroxy-6-(4-hydroxyphenethyl)-3-methylbenzoate	1p22	P35222	CTNNB1
2564	methyl 2,4-dihydroxy-6-(4-hydroxyphenethyl)-3-methylbenzoate	3e7o	P45984	MAPK9
2565	methyl 2,4-dihydroxy-6-(4-hydroxyphenethyl)-3-methylbenzoate	1w60	P12004	PCNA
2566	methyl 2,4-dihydroxy-6-(4-hydroxyphenethyl)-3-methylbenzoate	2w96	P11802	CDK4
2567	methyl 2,4-dihydroxy-6-(4-hydroxyphenethyl)-3-methylbenzoate	2ppi	Q9H2C0	GAN
2568	methyl 2,4-dihydroxy-6-(4-hydroxyphenethyl)-3-methylbenzoate	1x6a	P53671	LIMK2
2569	methyl 2,4-dihydroxy-6-(4-hydroxyphenethyl)-3-methylbenzoate	2h08	P60891	PRPS1
2570	methyl 2,4-dihydroxy-6-(4-hydroxyphenethyl)-3-methylbenzoate	1x86	Q9NZN5	ARHGEF12
2571	methyl 2,4-dihydroxy-6-(4-hydroxyphenethyl)-3-methylbenzoate	2ys5	O43559	FRS3
2572	methyl 2,4-dihydroxy-6-(4-hydroxyphenethyl)-3-methylbenzoate	2vtq	P24941	CDK2
2573	methyl 2,4-dihydroxy-6-(4-hydroxyphenethyl)-3-methylbenzoate	1w98	P24864	CCNE1
2574	methyl 2,4-dihydroxy-6-(4-hydroxyphenethyl)-3-methylbenzoate	1jv1	Q16222	UAP1
2575	methyl 2,4-dihydroxy-6-(4-hydroxyphenethyl)-3-methylbenzoate	2c9o	Q9Y265	RUVBL1
2576	methyl 2,4-dihydroxy-6-(4-hydroxyphenethyl)-3-methylbenzoate	1aii	P12429	ANXA3
2577	methyl 2,4-dihydroxy-6-(4-hydroxyphenethyl)-3-methylbenzoate	1nrg	Q9NVS9	PNPO
2578	methyl 2,4-dihydroxy-6-(4-hydroxyphenethyl)-3-methylbenzoate	1kgd	O14936	CASK
2579	methyl 2,4-dihydroxy-6-(4-hydroxyphenethyl)-3-methylbenzoate	2ig7	Q9Y259	CHKB
2580	methyl 2,4-dihydroxy-6-(4-hydroxyphenethyl)-3-methylbenzoate	2gl8	P48443	RXRG
2581	methyl 2,4-dihydroxy-6-(4-hydroxyphenethyl)-3-methylbenzoate	1jey	P12956	XRCC6
2582	methyl 2,4-dihydroxy-6-(4-hydroxyphenethyl)-3-methylbenzoate	2pom	Q15750	TAB1
2583	methyl 2,4-dihydroxy-6-(4-hydroxyphenethyl)-3-methylbenzoate	2pla	Q8N335	GPD1L
2584	methyl 2,4-dihydroxy-6-(4-hydroxyphenethyl)-3-methylbenzoate	1rxt	P30419	NMT1
2585	methyl 2,4-dihydroxy-6-(4-hydroxyphenethyl)-3-methylbenzoate	3bkb	P07332	FES
2586	methyl 2,4-dihydroxy-6-(4-hydroxyphenethyl)-3-methylbenzoate	1egw	Q02078	MEF2A
2587	methyl 2,4-dihydroxy-6-(4-hydroxyphenethyl)-3-methylbenzoate	2f3i	P52434	POLR2H
2588	methyl 2,4-dihydroxy-6-(4-hydroxyphenethyl)-3-methylbenzoate	3bg9	P11498	PC
2589	methyl 2,4-dihydroxy-6-(4-hydroxyphenethyl)-3-methylbenzoate	2e1o	Q03014	HHEX
2590	methyl 2,4-dihydroxy-6-(4-hydroxyphenethyl)-3-methylbenzoate	2iwg	P19474	TRIM21

2591	methyl 2,4-dihydroxy-6-(4-hydroxyphenethyl)-3-methylbenzoate	2nzt	P52789	HK2
2592	methyl 2,4-dihydroxy-6-(4-hydroxyphenethyl)-3-methylbenzoate	1w6j	P48449	LSS
2593	methyl 2,4-dihydroxy-6-(4-hydroxyphenethyl)-3-methylbenzoate	2a98	Q96DU7	ITPKC
2594	methyl 2,4-dihydroxy-6-(4-hydroxyphenethyl)-3-methylbenzoate	2i46	Q96AP0	ACD
2595	methyl 2,4-dihydroxy-6-(4-hydroxyphenethyl)-3-methylbenzoate	1wl4	Q9BWD1	ACAT2
2596	methyl 2,4-dihydroxy-6-(4-hydroxyphenethyl)-3-methylbenzoate	1pve	P54727	RAD23B
2597	salidroside	1wym	P37802	TAGLN2
2598	salidroside	1ry7	P05230	FGF1
2599	salidroside	2c9o	Q9Y265	RUVBL1
2600	salidroside	2ayn	P54578	USP14
2601	salidroside	2ch9	O76096	CST7
2602	salidroside	1rx0	Q9UKU7	ACAD8
2603	salidroside	3epz	P26358	DNMT1
2604	salidroside	2das	Q9UJ78	ZMYM5
2605	salidroside	1fqv	Q13309	SKP2
2606	salidroside	1zd1	Q9BR01	SULT4A1
2607	salidroside	1k78	Q02548	PAX5
2608	salidroside	1d5m	P01903	HLA-DRA
2609	salidroside	1uew	Q86UL8	MAGI2
2610	salidroside	2d9h	Q9BU19	ZNF692
2611	salidroside	2iwq	O75970	MPDZ
2612	salidroside	2edv	Q5SYB0	FRMPD1
2613	salidroside	1d6v	P01834	IGKC
2614	salidroside	1x66	Q01543	FLI1
2615	salidroside	1z0j	Q9H1K0	RBSN
2616	salidroside	1lar	P10586	PTPRF
2617	salidroside	2dmy	Q96SI9	STRBP
2618	salidroside	2fg5	Q13636	RAB31
2619	salidroside	2bc9	P32455	GBP1
2620	salidroside	3bkb	P07332	FES
2621	salidroside	2e1o	Q03014	HHEX
2622	salidroside	3d8e	O00213	APBB1
2623	salidroside	1jv1	Q16222	UAP1
2624	salidroside	1inz	Q9Y6I3	EPN1
2625	salidroside	2vkx	P13591	NCAM1
2626	salidroside	3bg0	P55735	SEC13
2627	salidroside	2jun	O15344	MID1
2628	salidroside	2wgh	P23921	RRM1
2629	salidroside	1w60	P12004	PCNA
2630	salidroside	2okk	Q05329	GAD2
2631	salidroside	2dlw	O60496	DOK2
2632	salidroside	2iwg	P19474	TRIM21
2633	salidroside	2o06	P19623	SRM
2634	salidroside	2dlu	Q8NI35	PATJ
2635	salidroside	2kdf	P55036	PSMD4
2636	salidroside	1s9c	P51659	HSD17B4
2637	salidroside	2csw	O76064	RNF8
2638	salidroside	3enm	P52564	MAP2K6
2639	salidroside	1q8k	P05198	EIF2S1
2640	salidroside	1aii	P12429	ANXA3
2641	salidroside	2ysd	Q96QZ7	MAGI1
2642	salidroside	1x6a	P53671	LIMK2
2643	salidroside	3dpl	Q93034	CUL5
2644	salidroside	2h08	P60891	PRPS1

2645	salidroside	3dkm	Q9ULT8	HECTD1
2646	salidroside	1w45	P13928	ANXA8
2647	salidroside	1w98	P24864	CCNE1
2648	salidroside	2ig7	Q9Y259	CHKB
2649	salidroside	1fsu	P15848	ARSB
2650	salidroside	3e7o	P45984	MAPK9
2651	salidroside	1wry	O75368	SH3BGRL
2652	salidroside	2crc	Q9BYM8	RBCK1
2653	salidroside	2e6o	O60381	HBP1
2654	salidroside	1fe8	P04275	VWF
2655	salidroside	2vr2	Q14117	DPYS
2656	salidroside	1exv	P06737	PYGL
2657	salidroside	2e8o	Q9Y3Z3	SAMHD1
2658	salidroside	2csh	O43298	ZBTB43
2659	salidroside	2gow	O95164	UBL3
2660	salidroside	2ji4	O60256	PRPSAP2
2661	salidroside	2a98	Q96DU7	ITPKC
2662	salidroside	3c0r	P0CG48	UBC
2663	salidroside	2pph	Q99759	MAP3K3
2664	salidroside	1unh	Q00535	CDK5
2665	salidroside	2cls	Q92730	RND1
2666	salidroside	2qqh	P07357	C8A
2667	salidroside	1pve	P54727	RAD23B
2668	salidroside	1so0	Q96C23	GALM
2669	salidroside	1nuf	Q08188	TGM3
2670	salidroside	2c0o	P08631	HCK
2671	salidroside	3c5k	Q9UBN7	HDAC6
2672	salidroside	2odq	P06681	C2
2673	salidroside	3eg9	Q15436	SEC23A
2674	salidroside	2ee4	Q13017	ARHGAP5
2675	pseudolaroside C	4i2x	Q9P1W8	SIRPG
2676	pseudolaroside C	3fmo	Q9UMR2	DDX19B
2677	pseudolaroside C	2ch9	O76096	CST7
2678	pseudolaroside C	2bka	Q9BUP3	HTATIP2
2679	pseudolaroside C	1uew	Q86UL8	MAGI2
2680	pseudolaroside C	1d5m	P01903	HLA-DRA
2681	pseudolaroside C	1wxb	Q9H6S3	EPS8L2
2682	pseudolaroside C	3g2f	Q13873	BMPR2
2683	pseudolaroside C	1ivh	P26440	IVD
2684	pseudolaroside C	2iwq	O75970	MPDZ
2685	pseudolaroside C	2ocb	Q9NP90	RAB9B
2686	pseudolaroside C	1z0j	Q9H1K0	RBSN
2687	pseudolaroside C	2w4r	Q96C10	DHX58
2688	pseudolaroside C	1igr	P08069	IGF1R
2689	pseudolaroside C	2ytc	Q9NW64	RBM22
2690	pseudolaroside C	2dgy	Q8N9N8	EIF1AD
2691	pseudolaroside C	2f57	Q9P286	PAK5
2692	pseudolaroside C	3f5n	Q99574	SERPINI1
2693	pseudolaroside C	3bsq	P49862	KLK7
2694	pseudolaroside C	1qo5	P05062	ALDOB
2695	pseudolaroside C	3bo5	Q53H47	SETMAR
2696	pseudolaroside C	2o06	P19623	SRM
2697	pseudolaroside C	3bg0	P55735	SEC13
2698	pseudolaroside C	2dmy	Q96SI9	STRBP

2699	pseudolaroside C	2qyf	Q13257	MAD2L1
2700	pseudolaroside C	2bc9	P32455	GBP1
2701	pseudolaroside C	1fg9	P15260	IFNGR1
2702	pseudolaroside C	3epz	P26358	DNMT1
2703	pseudolaroside C	2cpt	O75351	VPS4B
2704	pseudolaroside C	2ys5	O43559	FRS3
2705	pseudolaroside C	2vr2	Q14117	DPYS
2706	pseudolaroside C	1pkx	P31939	ATIC
2707	pseudolaroside C	1zxn	P11388	TOP2A
2708	pseudolaroside C	2pla	Q8N335	GPD1L
2709	pseudolaroside C	2d9w	O60496	DOK2
2710	pseudolaroside C	7ics	P06746	POLB
2711	pseudolaroside C	2cpy	Q9NTZ6	RBM12
2712	pseudolaroside C	2iwg	P19474	TRIM21
2713	pseudolaroside C	1xap	P10826	RARB
2714	pseudolaroside C	1wry	O75368	SH3BGRL
2715	pseudolaroside C	1uf0	O15075	DCLK1
2716	pseudolaroside C	2bxg	P02768	ALB
2717	pseudolaroside C	2ig7	Q9Y259	CHKB
2718	pseudolaroside C	1w60	P12004	PCNA
2719	pseudolaroside C	2kdf	P55036	PSMD4
2720	pseudolaroside C	2qq8	Q9P2M4	TBC1D14
2721	pseudolaroside C	2cmw	Q9HCP0	CSNK1G1
2722	pseudolaroside C	1jv1	Q16222	UAP1
2723	pseudolaroside C	3g1n	Q7Z6Z7	HUWE1
2724	pseudolaroside C	2enj	Q04759	PRKCQ
2725	pseudolaroside C	1fe8	P04275	VWF
2726	pseudolaroside C	2enk	Q6PML9	SLC30A9
2727	pseudolaroside C	3g65	O60671	RAD1
2728	pseudolaroside C	1n2l	P15289	ARSA
2729	pseudolaroside C	2j7q	P0CG48	UBC
2730	pseudolaroside C	1cf4	Q07912	TNK2
2731	pseudolaroside C	1lw3	Q13614	MTMR2
2732	pseudolaroside C	2ew1	Q15771	RAB30
2733	pseudolaroside C	1p22	P35222	CTNNB1
2734	pseudolaroside C	1w6j	P48449	LSS
2735	pseudolaroside C	3enm	P52564	MAP2K6
2736	pseudolaroside C	3bkb	P07332	FES
2737	pseudolaroside C	2v62	Q86Y07	VRK2
2738	pseudolaroside C	1x86	Q9NZN5	ARHGEF12
2739	pseudolaroside C	3d8e	O00213	APBB1
2740	pseudolaroside C	3eg9	Q15436	SEC23A
2741	pseudolaroside C	1pve	P54727	RAD23B
2742	pseudolaroside C	2cls	Q92730	RND1
2743	pseudolaroside C	2vcy	Q9BV79	MECR
2744	pseudolaroside C	2qqh	P07357	C8A
2745	pseudolaroside C	1onq	P06126	CD1A
2746	pseudolaroside C	2ebt	Q13887	KLF5
2747	pseudolaroside C	2ctq	Q9UKB3	DNAJC12
2748	pseudolaroside C	1nuf	Q08188	TGM3
2749	pseudolaroside C	1pj4	P23368	ME2
2750	pseudolaroside C	1exv	P06737	PYGL
2751	pseudolaroside C	2zot	Q9HCB6	SPON1
2752	pseudolaroside C	1lhl	P61626	LYZ

2753	pseudolaroside C	1unh	Q00535	CDK5
2754	rosin	2m0c	Q9H161	ALX4
2755	rosin	3fer	O75369	FLNB
2756	rosin	1x65	O75534	CSDE1
2757	rosin	1d5m	P01903	HLA-DRA
2758	rosin	1xjv	Q9NUX5	POT1
2759	rosin	1so0	Q96C23	GALM
2760	rosin	2jx3	P35659	DEK
2761	rosin	1mje	P60896	SEM1
2762	rosin	2iwq	O75970	MPDZ
2763	rosin	3d3l	P18054	ALOX12
2764	rosin	1khu	Q15797	SMAD1
2765	rosin	2ayn	P54578	USP14
2766	rosin	1w98	P24864	CCNE1
2767	rosin	3d8e	O00213	APBB1
2768	rosin	2iwg	P19474	TRIM21
2769	rosin	1tg6	Q16740	CLPP
2770	rosin	3bkb	P07332	FES
2771	rosin	2fg5	Q13636	RAB31
2772	rosin	1jv1	Q16222	UAP1
2773	rosin	1wry	O75368	SH3BGRL
2774	rosin	1x6a	P53671	LIMK2
2775	rosin	1w45	P13928	ANXA8
2776	rosin	3dpl	Q93034	CUL5
2777	rosin	2dlu	Q8NI35	PATJ
2778	rosin	1ow1	Q96T58	SPEN
2779	rosin	2da7	O60315	ZEB2
2780	rosin	3e7o	P45984	MAPK9
2781	rosin	2enk	Q6PML9	SLC30A9
2782	rosin	2bc9	P32455	GBP1
2783	rosin	3bum	O43597	SPRY2
2784	rosin	2j91	P30566	ADSL
2785	rosin	1w6j	P48449	LSS
2786	rosin	1fo3	Q9UKM7	MAN1B1
2787	rosin	1w60	P12004	PCNA
2788	rosin	1vyh	P68402	PAFAH1B2
2789	rosin	2raj	Q9Y5X1	SNX9
2790	rosin	1unh	Q00535	CDK5
2791	rosin	2c2h	P60763	RAC3
2792	rosin	1l9x	Q92820	GGH
2793	rosin	1fe8	P04275	VWF
2794	rosin	1wh0	O94966	USP19
2795	rosin	3bg0	P55735	SEC13
2796	rosin	2jun	O15344	MID1
2797	rosin	2nn6	Q06265	EXOSC9
2798	rosin	2vkx	P13591	NCAM1
2799	rosin	3g1n	Q7Z6Z7	HUWE1
2800	rosin	2ysd	Q96QZ7	MAGI1
2801	rosin	2coa	Q9BZL6	PRKD2
2802	rosin	2csw	O76064	RNF8
2803	rosin	2edu	Q14807	KIF22
2804	rosin	3bky	P11836	MS4A1
2805	rosin	2ep4	Q9Y225	RNF24
2806	rosin	2j7q	P0CG48	UBC

2807	rosin	2cmw	Q9HCP0	CSNK1G1
2808	rosin	2a98	Q96DU7	ITPKC
2809	rosin	1r6u	P23381	WARS1
2810	rosin	2ee4	Q13017	ARHGAP5
2811	rosin	2pph	Q99759	MAP3K3
2812	rosin	7fab	P01825	IGHV4-59
2813	rosin	2yuc	Q9BUZ4	TRAF4
2814	rosin	2pom	Q15750	TAB1
2815	rosin	3eay	Q9BQF6	SENP7
2816	rosin	3dkm	Q9ULT8	HECTD1
2817	rosin	1xk0	P09601	HMOX1
2818	rosin	1t9g	P11310	ACADM
2819	rosin	2ast	P46527	CDKN1B
2820	rosin	2vag	P49759	CLK1
2821	rosin	2c0o	P08631	HCK
2822	rosin	2r55	Q9NSY2	STARD5
2823	rosin	2qqh	P07357	C8A
2824	(S)-2,4-dibutoxy-3-(hydroxymethyl)cyclopent-2-en-1-one	2ebz	O14924	RGS12
2825	(S)-2,4-dibutoxy-3-(hydroxymethyl)cyclopent-2-en-1-one	1ujv	Q86UL8	MAGI2
2826	(S)-2,4-dibutoxy-3-(hydroxymethyl)cyclopent-2-en-1-one	1lb1	P61586	RHOA
2827	(S)-2,4-dibutoxy-3-(hydroxymethyl)cyclopent-2-en-1-one	2hye	Q16531	DDB1
2828	(S)-2,4-dibutoxy-3-(hydroxymethyl)cyclopent-2-en-1-one	2cue	P26367	PAX6
2829	(S)-2,4-dibutoxy-3-(hydroxymethyl)cyclopent-2-en-1-one	1n8z	P04626	ERBB2
2830	(S)-2,4-dibutoxy-3-(hydroxymethyl)cyclopent-2-en-1-one	2z5v	Q99836	MYD88
2831	(S)-2,4-dibutoxy-3-(hydroxymethyl)cyclopent-2-en-1-one	1xjv	Q9NUX5	POT1
2832	(S)-2,4-dibutoxy-3-(hydroxymethyl)cyclopent-2-en-1-one	2fg5	Q13636	RAB31
2833	(S)-2,4-dibutoxy-3-(hydroxymethyl)cyclopent-2-en-1-one	2z6e	Q9Y4D1	DAAM1
2834	(S)-2,4-dibutoxy-3-(hydroxymethyl)cyclopent-2-en-1-one	1cok	O15350	TP73
2835	(S)-2,4-dibutoxy-3-(hydroxymethyl)cyclopent-2-en-1-one	1igr	P08069	IGF1R
2836	(S)-2,4-dibutoxy-3-(hydroxymethyl)cyclopent-2-en-1-one	2ch9	O76096	CST7
2837	(S)-2,4-dibutoxy-3-(hydroxymethyl)cyclopent-2-en-1-one	1em2	Q14849	STARD3
2838	(S)-2,4-dibutoxy-3-(hydroxymethyl)cyclopent-2-en-1-one	2cra	Q92826	HOXB13
2839	(S)-2,4-dibutoxy-3-(hydroxymethyl)cyclopent-2-en-1-one	1ggt	P00488	F13A1
2840	(S)-2,4-dibutoxy-3-(hydroxymethyl)cyclopent-2-en-1-one	3g2f	Q13873	BMPR2
2841	(S)-2,4-dibutoxy-3-(hydroxymethyl)cyclopent-2-en-1-one	1jey	P12956	XRCC6
2842	(S)-2,4-dibutoxy-3-(hydroxymethyl)cyclopent-2-en-1-one	3bch	P08865	RPSA
2843	(S)-2,4-dibutoxy-3-(hydroxymethyl)cyclopent-2-en-1-one	1o9k	P06400	RB1
2844	(S)-2,4-dibutoxy-3-(hydroxymethyl)cyclopent-2-en-1-one	1khu	Q15797	SMAD1
2845	(S)-2,4-dibutoxy-3-(hydroxymethyl)cyclopent-2-en-1-one	1s9c	P51659	HSD17B4
2846	(S)-2,4-dibutoxy-3-(hydroxymethyl)cyclopent-2-en-1-one	1tg6	Q16740	CLPP
2847	(S)-2,4-dibutoxy-3-(hydroxymethyl)cyclopent-2-en-1-one	1ya0	Q92540	SMG7
2848	(S)-2,4-dibutoxy-3-(hydroxymethyl)cyclopent-2-en-1-one	7fab	P01825	IGHV4-59
2849	(S)-2,4-dibutoxy-3-(hydroxymethyl)cyclopent-2-en-1-one	3eay	Q9BQF6	SENP7
2850	(S)-2,4-dibutoxy-3-(hydroxymethyl)cyclopent-2-en-1-one	1zag	P25311	AZGP1
2851	(S)-2,4-dibutoxy-3-(hydroxymethyl)cyclopent-2-en-1-one	2uxw	P49748	ACADVL
2852	(S)-2,4-dibutoxy-3-(hydroxymethyl)cyclopent-2-en-1-one	2da7	O60315	ZEB2
2853	(S)-2,4-dibutoxy-3-(hydroxymethyl)cyclopent-2-en-1-one	2pp4	Q06455	RUNX1T1
2854	(S)-2,4-dibutoxy-3-(hydroxymethyl)cyclopent-2-en-1-one	1ow1	Q96T58	SPEN
2855	(S)-2,4-dibutoxy-3-(hydroxymethyl)cyclopent-2-en-1-one	3dkm	Q9ULT8	HECTD1
2856	(S)-2,4-dibutoxy-3-(hydroxymethyl)cyclopent-2-en-1-one	3e7o	P45984	MAPK9
2857	(S)-2,4-dibutoxy-3-(hydroxymethyl)cyclopent-2-en-1-one	2vz6	Q9UQM7	CAMK2A
2858	(S)-2,4-dibutoxy-3-(hydroxymethyl)cyclopent-2-en-1-one	1x65	O75534	CSDE1
2859	(S)-2,4-dibutoxy-3-(hydroxymethyl)cyclopent-2-en-1-one	3bkb	P07332	FES
2860	(S)-2,4-dibutoxy-3-(hydroxymethyl)cyclopent-2-en-1-one	1egw	Q02078	MEF2A

2861	(S)-2,4-dibutoxy-3-(hydroxymethyl)cyclopent-2-en-1-one	2yu4	Q96MF7	NSMCE2
2862	(S)-2,4-dibutoxy-3-(hydroxymethyl)cyclopent-2-en-1-one	2ysd	Q96QZ7	MAGI1
2863	(S)-2,4-dibutoxy-3-(hydroxymethyl)cyclopent-2-en-1-one	2ep8	O00541	PES1
2864	(S)-2,4-dibutoxy-3-(hydroxymethyl)cyclopent-2-en-1-one	2z7x	Q15399	TLR1
2865	(S)-2,4-dibutoxy-3-(hydroxymethyl)cyclopent-2-en-1-one	2csw	O76064	RNF8
2866	(S)-2,4-dibutoxy-3-(hydroxymethyl)cyclopent-2-en-1-one	3bum	O43597	SPRY2
2867	(S)-2,4-dibutoxy-3-(hydroxymethyl)cyclopent-2-en-1-one	3bg9	P11498	PC
2868	(S)-2,4-dibutoxy-3-(hydroxymethyl)cyclopent-2-en-1-one	3cwz	P20340	RAB6A
2869	(S)-2,4-dibutoxy-3-(hydroxymethyl)cyclopent-2-en-1-one	2nn6	Q06265	EXOSC9
2870	(S)-2,4-dibutoxy-3-(hydroxymethyl)cyclopent-2-en-1-one	1w6j	P48449	LSS
2871	(S)-2,4-dibutoxy-3-(hydroxymethyl)cyclopent-2-en-1-one	1n46	P10828	THRB
2872	(S)-2,4-dibutoxy-3-(hydroxymethyl)cyclopent-2-en-1-one	2nzt	P52789	HK2
2873	(S)-2,4-dibutoxy-3-(hydroxymethyl)cyclopent-2-en-1-one	2bc9	P32455	GBP1
2874	(S)-2,4-dibutoxy-3-(hydroxymethyl)cyclopent-2-en-1-one	1lw3	Q13614	MTMR2
2875	(S)-2,4-dibutoxy-3-(hydroxymethyl)cyclopent-2-en-1-one	2e6i	Q08881	ITK
2876	(S)-2,4-dibutoxy-3-(hydroxymethyl)cyclopent-2-en-1-one	1w45	P13928	ANXA8
2877	(S)-2,4-dibutoxy-3-(hydroxymethyl)cyclopent-2-en-1-one	2c2h	P60763	RAC3
2878	(S)-2,4-dibutoxy-3-(hydroxymethyl)cyclopent-2-en-1-one	3enm	P52564	MAP2K6
2879	(S)-2,4-dibutoxy-3-(hydroxymethyl)cyclopent-2-en-1-one	1d5b	P01834	IGKC
2880	(S)-2,4-dibutoxy-3-(hydroxymethyl)cyclopent-2-en-1-one	2aff	P46013	MKI67
2881	(S)-2,4-dibutoxy-3-(hydroxymethyl)cyclopent-2-en-1-one	2ig7	Q9Y259	CHKB
2882	(S)-2,4-dibutoxy-3-(hydroxymethyl)cyclopent-2-en-1-one	1wry	O75368	SH3BGRL
2883	(S)-2,4-dibutoxy-3-(hydroxymethyl)cyclopent-2-en-1-one	3bky	P11836	MS4A1
2884	(S)-2,4-dibutoxy-3-(hydroxymethyl)cyclopent-2-en-1-one	1fsu	P15848	ARSB
2885	(S)-2,4-dibutoxy-3-(hydroxymethyl)cyclopent-2-en-1-one	2dmz	Q8NI35	PATJ
2886	(S)-2,4-dibutoxy-3-(hydroxymethyl)cyclopent-2-en-1-one	2okk	Q05329	GAD2
2887	(S)-2,4-dibutoxy-3-(hydroxymethyl)cyclopent-2-en-1-one	1unh	Q00535	CDK5
2888	(S)-2,4-dibutoxy-3-(hydroxymethyl)cyclopent-2-en-1-one	2dlz	P52735	VAV2
2889	(S)-2,4-dibutoxy-3-(hydroxymethyl)cyclopent-2-en-1-one	2agj	P01871	IGHM
2890	(S)-2,4-dibutoxy-3-(hydroxymethyl)cyclopent-2-en-1-one	1wi3	Q9UPW6	SATB2
2891	(S)-2,4-dibutoxy-3-(hydroxymethyl)cyclopent-2-en-1-one	2hzp	Q16719	KYNU
2892	(S)-2,4-dibutoxy-3-(hydroxymethyl)cyclopent-2-en-1-one	1x4a	Q07955	SRSF1
2893	(S)-2,4-dibutoxy-3-(hydroxymethyl)cyclopent-2-en-1-one	3b68	P10275	AR
2894	(S)-2,4-dibutoxy-3-(hydroxymethyl)cyclopent-2-en-1-one	2om5	Q02246	CNTN2
2895	(S)-2,4-dibutoxy-3-(hydroxymethyl)cyclopent-2-en-1-one	3cb2	P23258	TUBG1
2896	(S)-2,4-dibutoxy-3-(hydroxymethyl)cyclopent-2-en-1-one	2vag	P49759	CLK1
2897	(S)-2,4-dibutoxy-3-(hydroxymethyl)cyclopent-2-en-1-one	2w7r	O15021	MAST4
2898	(S)-2,4-dibutoxy-3-(hydroxymethyl)cyclopent-2-en-1-one	2pph	Q99759	MAP3K3
2899	(S)-2,4-dibutoxy-3-(hydroxymethyl)cyclopent-2-en-1-one	1y8o	Q15120	PDK3
2900	(S)-2,4-dibutoxy-3-(hydroxymethyl)cyclopent-2-en-1-one	2h2u	Q8WVQ1	CANT1
2901	(S)-2,4-dibutoxy-3-(hydroxymethyl)cyclopent-2-en-1-one	2f8x	P46531	NOTCH1
2902	hydroxydihydrobovolide	1buv	P50281	MMP14
2903	hydroxydihydrobovolide	2vre	Q13011	ECH1
2904	hydroxydihydrobovolide	2qfd	O95786	DDX58
2905	hydroxydihydrobovolide	2eon	Q9Y2L8	ZKSCAN5
2906	hydroxydihydrobovolide	2qq5	Q96LJ7	DHRS1
2907	hydroxydihydrobovolide	1n8z	P04626	ERBB2
2908	hydroxydihydrobovolide	2i7k	Q9NPI1	BRD7
2909	hydroxydihydrobovolide	1i1r	P40189	IL6ST
2910	hydroxydihydrobovolide	3bhd	Q9BU02	THTPA
2911	hydroxydihydrobovolide	1a6z	P61769	B2M
2912	hydroxydihydrobovolide	2ecd	P42684	ABL2
2913	hydroxydihydrobovolide	3d3l	P18054	ALOX12
2914	hydroxydihydrobovolide	1snl	Q02818	NUCB1

2915	hydroxydihydrobovolide	1zbu	Q8IV48	ERI1
2916	hydroxydihydrobovolide	1so0	Q96C23	GALM
2917	hydroxydihydrobovolide	2bk3	P27338	MAOB
2918	hydroxydihydrobovolide	2ayn	P54578	USP14
2919	hydroxydihydrobovolide	2pkt	O00151	PDLIM1
2920	hydroxydihydrobovolide	2fy4	P28329	CHAT
2921	hydroxydihydrobovolide	2vig	P16219	ACADS
2922	hydroxydihydrobovolide	1uw4	Q9BZI7	UPF3B
2923	hydroxydihydrobovolide	1zvs	P61769	B2M
2924	hydroxydihydrobovolide	2dgu	O60506	SYNCRIP
2925	hydroxydihydrobovolide	2enj	Q04759	PRKCQ
2926	hydroxydihydrobovolide	1e51	P13716	ALAD
2927	hydroxydihydrobovolide	1cok	O15350	TP73
2928	hydroxydihydrobovolide	1jmj	P05546	SERPIND1
2929	hydroxydihydrobovolide	2vpk	Q9NPC7	MYNN
2930	hydroxydihydrobovolide	2h63	P53004	BLVRA
2931	hydroxydihydrobovolide	1tkn	P05067	APP
2932	hydroxydihydrobovolide	3c5k	Q9UBN7	HDAC6
2933	hydroxydihydrobovolide	1wh0	O94966	USP19
2934	hydroxydihydrobovolide	1ow1	Q96T58	SPEN
2935	hydroxydihydrobovolide	1zag	P25311	AZGP1
2936	hydroxydihydrobovolide	2w96	P11802	CDK4
2937	hydroxydihydrobovolide	2fg5	Q13636	RAB31
2938	hydroxydihydrobovolide	1w60	P12004	PCNA
2939	hydroxydihydrobovolide	3dl2	Q8IX04	UEVLD
2940	hydroxydihydrobovolide	1k1g	Q15637	SF1
2941	hydroxydihydrobovolide	1jv1	Q16222	UAP1
2942	hydroxydihydrobovolide	1w45	P13928	ANXA8
2943	hydroxydihydrobovolide	2vr2	Q14117	DPYS
2944	hydroxydihydrobovolide	2d9n	O95639	CPSF4
2945	hydroxydihydrobovolide	2ozb	P55769	SNU13
2946	hydroxydihydrobovolide	2hye	Q16531	DDB1
2947	hydroxydihydrobovolide	2pom	Q15750	TAB1
2948	hydroxydihydrobovolide	2csw	O76064	RNF8
2949	hydroxydihydrobovolide	3bkb	P07332	FES
2950	hydroxydihydrobovolide	1aii	P12429	ANXA3
2951	hydroxydihydrobovolide	1qgk	P52292	KPNA2
2952	hydroxydihydrobovolide	2ig7	Q9Y259	CHKB
2953	hydroxydihydrobovolide	2vwe	P49765	VEGFB
2954	hydroxydihydrobovolide	1w6j	P48449	LSS
2955	hydroxydihydrobovolide	1jey	P12956	XRCC6
2956	hydroxydihydrobovolide	2gl8	P48443	RXRG
2957	hydroxydihydrobovolide	2ejr	O60341	KDM1A
2958	hydroxydihydrobovolide	2coa	Q9BZL6	PRKD2
2959	hydroxydihydrobovolide	2f9d	O75533	SF3B1
2960	hydroxydihydrobovolide	1rxt	P30419	NMT1
2961	hydroxydihydrobovolide	2dmz	Q8NI35	PATJ
2962	hydroxydihydrobovolide	2f3i	P52434	POLR2H
2963	hydroxydihydrobovolide	2cpt	O75351	VPS4B
2964	hydroxydihydrobovolide	2ys5	O43559	FRS3
2965	hydroxydihydrobovolide	2a98	Q96DU7	ITPKC
2966	hydroxydihydrobovolide	1lqs	Q13651	IL10RA
2967	hydroxydihydrobovolide	2i7t	Q9UKF6	CPSF3
2968	hydroxydihydrobovolide	2c2h	P60763	RAC3

2969	hydroxydihydrobovolide	1fe8	P04275	VWF
2970	hydroxydihydrobovolide	2e29	Q9BQ39	DDX50
2971	α-linolenic acid	3q8p	Q9UNA4	POLI
2972	α-linolenic acid	3dz7	P17707	AMD1
2973	α-linolenic acid	1cmx	P0CG48	UBC
2974	α-linolenic acid	2v0v	Q14995	NR1D2
2975	α-linolenic acid	2c9o	Q9Y265	RUVBL1
2976	α-linolenic acid	1jfi	Q01658	DR1
2977	α-linolenic acid	2es0	P49758	RGS6
2978	α-linolenic acid	3cki	P78536	ADAM17
2979	α-linolenic acid	2pp4	Q06455	RUNX1T1
2980	α-linolenic acid	2cfy	Q16881	TXNRD1
2981	α-linolenic acid	2os6	Q9NZN5	ARHGEF12
2982	α-linolenic acid	1r8u	Q99967	CITED2
2983	α-linolenic acid	1zte	P04179	SOD2
2984	α-linolenic acid	2f8n	O75367	MACROH2A1
2985	α-linolenic acid	2h08	P60891	PRPS1
2986	α-linolenic acid	1x65	O75534	CSDE1
2987	α-linolenic acid	1s9c	P51659	HSD17B4
2988	α-linolenic acid	2ayn	P54578	USP14
2989	α-linolenic acid	1n46	P10828	THRB
2990	α-linolenic acid	1yhn	P51149	RAB7A
2991	α-linolenic acid	2gk9	Q8TBX8	PIP4K2C
2992	α-linolenic acid	1gzq	P29016	CD1B
2993	α-linolenic acid	1x5n	Q9Y6N9	USH1C
2994	α-linolenic acid	1iq3	Q8NFH8	REPS2
2995	α-linolenic acid	1so0	Q96C23	GALM
2996	α-linolenic acid	1lqs	Q13651	IL10RA
2997	α-linolenic acid	1x9n	P18858	LIG1
2998	α-linolenic acid	3epy	Q8N6N7	ACBD7
2999	α-linolenic acid	3bm4	Q9UKK9	NUDT5
3000	α-linolenic acid	1zag	P25311	AZGP1
3001	α-linolenic acid	1xap	P10826	RARB
3002	α-linolenic acid	1bh9	Q15543	TAF13
3003	α-linolenic acid	1tg6	Q16740	CLPP
3004	α-linolenic acid	1ivh	P26440	IVD
3005	α-linolenic acid	2rnx	Q92831	KAT2B
3006	α-linolenic acid	1yb1	Q8NBQ5	HSD17B11
3007	α-linolenic acid	1xjv	Q9NUX5	POT1
3008	α-linolenic acid	3d3l	P18054	ALOX12
3009	α-linolenic acid	1zvs	P61769	B2M
3010	α-linolenic acid	2vpk	Q9NPC7	MYNN
3011	α-linolenic acid	2p54	Q07869	PPARA
3012	α-linolenic acid	1x1f	Q9ULZ2	STAP1
3013	α-linolenic acid	3eap	Q6P4F7	ARHGAP11A
3014	α-linolenic acid	1pkx	P31939	ATIC
3015	α-linolenic acid	1nu9	P00734	F2
3016	α-linolenic acid	1jn5	Q9UBU9	NXF1
3017	α-linolenic acid	1ggz	P27482	CALML3
3018	α-linolenic acid	1tkn	P05067	APP
3019	α-linolenic acid	2dk2	O43390	HNRNPR
3020	α-linolenic acid	2p0r	O14815	CAPN9
3021	α-linolenic acid	2r0n	Q92947	GCDH
3022	α-linolenic acid	2oay	P05155	SERPING1

3023	α-linolenic acid	2daf	Q8NA54	IQUB
3024	α-linolenic acid	2gl8	P48443	RXRG
3025	α-linolenic acid	3b68	P10275	AR
3026	α-linolenic acid	3g73	Q08050	FOXM1
3027	α-linolenic acid	1xdt	Q99075	HBEGF
3028	α-linolenic acid	1k1g	Q15637	SF1
3029	α-linolenic acid	2w4o	Q16566	CAMK4
3030	α-linolenic acid	1w6j	P48449	LSS
3031	α-linolenic acid	1wh0	O94966	USP19
3032	α-linolenic acid	1d5b	P01834	IGKC
3033	α-linolenic acid	1us1	Q16853	AOC3
3034	α-linolenic acid	1w98	P24864	CCNE1
3035	α-linolenic acid	1qls	P04083	ANXA1
3036	α-linolenic acid	2bsk	P62072	TIMM10
3037	α-linolenic acid	2rfj	Q58F21	BRDT
3038	α-linolenic acid	2k8f	Q09472	EP300
3039	α-linolenic acid	1qib	P08253	MMP2
3040	α-linolenic acid	2acm	P15941	MUC1
3041	α-linolenic acid	1h9u	P28702	RXRB
3042	α-linolenic acid	1mj4	P51687	SUOX
3043	α-linolenic acid	1ow1	Q96T58	SPEN
3044	α-linolenic acid	1rkh	Q15648	MED1
3045	α-linolenic acid	2bc9	P32455	GBP1
3046	linoleic acid	2v0v	Q14995	NR1D2
3047	linoleic acid	1jfi	Q01658	DR1
3048	linoleic acid	2c9o	Q9Y265	RUVBL1
3049	linoleic acid	1xke	P49792	RANBP2
3050	linoleic acid	2es0	P49758	RGS6
3051	linoleic acid	1r8u	Q99967	CITED2
3052	linoleic acid	1nu9	P00734	F2
3053	linoleic acid	2h08	P60891	PRPS1
3054	linoleic acid	1fcx	P13631	RARG
3055	linoleic acid	1x65	O75534	CSDE1
3056	linoleic acid	2os6	Q9NZN5	ARHGEF12
3057	linoleic acid	1zte	P04179	SOD2
3058	linoleic acid	2f8n	O75367	MACROH2A1
3059	linoleic acid	2ayn	P54578	USP14
3060	linoleic acid	1xjv	Q9NUX5	POT1
3061	linoleic acid	1gzq	P29016	CD1B
3062	linoleic acid	1iq3	Q8NFH8	REPS2
3063	linoleic acid	1bh9	Q15543	TAF13
3064	linoleic acid	2w4o	Q16566	CAMK4
3065	linoleic acid	2gk9	Q8TBX8	PIP4K2C
3066	linoleic acid	2czy	Q13127	REST
3067	linoleic acid	3bm4	Q9UKK9	NUDT5
3068	linoleic acid	1s9c	P51659	HSD17B4
3069	linoleic acid	1lqs	Q13651	IL10RA
3070	linoleic acid	1yhn	P51149	RAB7A
3071	linoleic acid	1x9n	P18858	LIG1
3072	linoleic acid	3epy	Q8N6N7	ACBD7
3073	linoleic acid	2oay	P05155	SERPING1
3074	linoleic acid	1so0	Q96C23	GALM
3075	linoleic acid	3dof	P36404	ARL2
3076	linoleic acid	1n46	P10828	THRB

3077	linoleic acid	1myp	P05164	MPO
3078	linoleic acid	2gl8	P48443	RXRG
3079	linoleic acid	1tg6	Q16740	CLPP
3080	linoleic acid	1zag	P25311	AZGP1
3081	linoleic acid	3b68	P10275	AR
3082	linoleic acid	1us1	Q16853	AOC3
3083	linoleic acid	1d5b	P01834	IGKC
3084	linoleic acid	2do1	P82979	SARNP
3085	linoleic acid	1wh0	O94966	USP19
3086	linoleic acid	1k1g	Q15637	SF1
3087	linoleic acid	2da7	O60315	ZEB2
3088	linoleic acid	1inz	Q9Y6I3	EPN1
3089	linoleic acid	2zu6	P60842	EIF4A1
3090	linoleic acid	1u6g	Q13616	CUL1
3091	linoleic acid	2acm	P15941	MUC1
3092	linoleic acid	1gqm	P80511	S100A12
3093	linoleic acid	3g73	Q08050	FOXM1
3094	linoleic acid	2daf	Q8NA54	IQUB
3095	linoleic acid	1mj4	P51687	SUOX
3096	linoleic acid	2rfj	Q58F21	BRDT
3097	linoleic acid	2e29	Q9BQ39	DDX50
3098	linoleic acid	1qls	P04083	ANXA1
3099	linoleic acid	2yt7	O96018	APBA3
3100	linoleic acid	1g3m	P49888	SULT1E1
3101	linoleic acid	1w98	P24864	CCNE1
3102	linoleic acid	2vwe	P49765	VEGFB
3103	linoleic acid	2bsk	P62072	TIMM10
3104	linoleic acid	1xdt	Q99075	HBEGF
3105	linoleic acid	1prx	P30041	PRDX6
3106	linoleic acid	1wi3	Q9UPW6	SATB2
3107	linoleic acid	1qib	P08253	MMP2
3108	linoleic acid	1ow1	Q96T58	SPEN
3109	linoleic acid	1jfn	P08519	LPA
3110	linoleic acid	2dme	Q92576	PHF3
3111	linoleic acid	2ee4	Q13017	ARHGAP5
3112	linoleic acid	1x5n	Q9Y6N9	USH1C

Table S2 Targets of depressive disorder obtained from database

NO	Uniprot ID	Gene names	Protein Names	database
1	P01023	A2M	Alpha-2-Macroglobulin	disgenet
2	Q16613	AANAT	Aralkylamine N-Acetyltransferase	disgenet
3	P08183	ABCB1	ATP-Dependent Translocase ABCB1	disgenet
4	Q7Z7G0	ABI3BP	ABI Family Member 3 Binding Protein	disgenet
5	P12821	ACE	Angiotensin-Converting Enzyme	TTD, disgenet, pharmGBK
6	P24666	ACP1	Acid Phosphatase 1	disgenet
7	O60488	ACSL4	Acyl-Coa Synthetase Long Chain Family Member 4	disgenet
8	Q08AH1	ACSM1	Acyl-Coa Synthetase Medium Chain Family Member 1	disgenet
9	O95622	ADCY5	Adenylate Cyclase 5	disgenet
10	P51828	ADCY7	Adenylate Cyclase 7	disgenet
11	P40145	ADCY8	Adenylate Cyclase 8	disgenet
12	P18509	ADCYAP1	Adenylate Cyclase Activating Polypeptide 1	disgenet
13	P35318	ADM	ADM [Cleaved Into: Adrenomedullin	pharmGBK
14	P29274	ADORA2A	Adenosine A2a Receptor	disgenet
15	P35348	ADRA1A	Alpha-1A Adrenergic Receptor	TTD
16	P08913	ADRA2A	Alpha-2A Adrenergic Receptor	TTD, disgenet, pharmGBK
17	P08588	ADRB1	Adrenoceptor Beta 1	disgenet
18	P01019	AGT	Angiotensinogen	disgenet
19	O43823	AKAP8	A-Kinase Anchoring Protein 8	disgenet
20	P31749	AKT1	AKT Serine/Threonine Kinase 1	disgenet
21	P02768	ALB	Albumin	disgenet
22	Q9BVK2	ALG8	ALG8, Alpha-1,3-Glucosyltransferase	disgenet
23	Q9UM73	ALK	ALK Receptor Tyrosine Kinase	disgenet
24	Q8NFD2	ANKK1	Ankyrin Repeat And Kinase Domain Containing 1	disgenet
25	Q9NQ90	ANO2	Anoctamin-2	pharmGBK
26	P15144	ANPEP	Alanyl Aminopeptidase, Membrane	disgenet
27	O14727	APAF1	Apoptotic Protease-Activating Factor 1	OMIM
28	P02649	APOE	Apolipoprotein E	OMIM
29	P05067	APP	Amyloid Beta Precursor Protein	disgenet
30	P07741	APRT	Adenine Phosphoribosyltransferase	disgenet
31	P55087	AQP4	Aquaporin 4	disgenet
32	P10275	AR	Androgen Receptor	disgenet
33	O15013	ARHGEF10	Rho Guanine Nucleotide Exchange Factor 10	disgenet
34	O00327	ARNTL	Aryl Hydrocarbon Receptor Nuclear Translocator Like	disgenet
35	P49407	ARRB1	Arrestin Beta 1	disgenet
36	P32121	ARRB2	Arrestin Beta 2	disgenet
37	P15289	ARSA	Arylsulfatase A	disgenet
38	Q5T4W7	ARTN	Artemin	disgenet
39	P46597	ASMT	Acetylserotonin O-Methyltransferase	disgenet
40	P18847	ATF3	Activating Transcription Factor 3	disgenet
41	P18848	ATF4	Activating Transcription Factor 4	disgenet
42	O60312	ATP10A	Probable Phospholipid-Transporting Atpase VA	pharmGBK

43	P13637	ATP1A3	Sodium/Potassium-Transporting Atpase Subunit Alpha-3	disgenet
44	P16615	ATP2A2	Atpase Sarcoplasmic/Endoplasmic Reticulum Ca2+ Transporting 2	disgenet
45	P35670	ATP7B	Atpase Copper Transporting Beta	disgenet
46	P54252	ATXN3	Ataxin 3	disgenet
47	P01185	AVP	Arginine Vasopressin	disgenet
48	Q99933	BAG1	BCL2 Associated Athanogene 1	disgenet
49	P10415	BCL2	BCL2, Apoptosis Regulator	disgenet
50	O00512	BCL9	B Cell CLL/Lymphoma 9	disgenet
51	P11274	BCR	BCR, Rhogef And Gtpase Activating Protein	disgenet
52	P46663	BDKRB1	B1 Bradykinin Receptor	TTD
53	P30411	BDKRB2	Bradykinin Receptor B2	disgenet
54	P23560	BDNF	Brain-Derived Neurotrophic Factor	disgenet, OMIM, pharmGBK
55	Q9H694	BICC1	Bicc Family RNA Binding Protein 1	disgenet
56	P18075	BMP7	Bone Morphogenetic Protein 7	disgenet
57	P38398	BRCA1	BRCA1, DNA Repair Associated	disgenet
58	Q96G97	BSCL2	Seipin	OMIM
59	Q9UPA5	BSN	Protein Bassoon	OMIM
60	O00555	CACNA1A	Voltage-Dependent P/Q-Type Calcium Channel Subunit Alpha-1A	OMIM, pharmGBK
61	Q13936	CACNA1C	Voltage-Dependent L-Type Calcium Channel Subunit Alpha-1C	disgenet, pharmGBK
62	Q9BY67	CADM1	Cell Adhesion Molecule 1	disgenet
63	P06881	CALCA	Calcitonin Gene-Related Peptide 1	OMIM
64	P0DP24	CALM2	Calmodulin 2	disgenet
65	Q9UQM7	CAMK2A	Calcium/Calmodulin Dependent Protein Kinase II Alpha	disgenet
66	Q13554	CAMK2B	Calcium/Calmodulin-Dependent Protein Kinase Type II Subunit Beta	OMIM
67	Q16568	CARTPT	CART Prepropeptide	disgenet
68	P42574	CASP3	Caspase-3	OMIM
69	P04040	CAT	Catalase	disgenet
70	Q6P1N0	CC2D1A	Coiled-Coil And C2 Domain Containing 1A	disgenet
71	P32238	CCKAR	Cholecystokinin A Receptor	disgenet
72	P13500	CCL2	C-C Motif Chemokine Ligand 2	disgenet
73	O00175	CCL24	C-C Motif Chemokine Ligand 24	disgenet
74	P24385	CCND1	Cyclin D1	disgenet
75	P28906	CD34	CD34 Molecule	disgenet
76	P55290	CDH13	Cadherin 13	disgenet
77	Q9ULB5	CDH7	Cadherin 7	disgenet
78	P42771	CDKN2A	Cyclin Dependent Kinase Inhibitor 2A	disgenet
79	P28329	CHAT	Choline O-Acetyltransferase	disgenet
80	P08172	CHRM2	Muscarinic Acetylcholine Receptor M2	disgenet, OMIM
81	P08912	CHRM5	Muscarinic Acetylcholine Receptor M5	TTD
82	Q15822	CHRNA2	Neuronal Acetylcholine Receptor Subunit Alpha-2	TTD

83	P43681	CHRNA4	Cholinergic Receptor Nicotinic Alpha 4 Subunit	disgenet
84	Q15825	CHRNA6	Cholinergic Receptor Nicotinic Alpha 6 Subunit	disgenet
85	Q05901	CHRNB3	Cholinergic Receptor Nicotinic Beta 3 Subunit	disgenet
86	O15516	CLOCK	Circadian Locomoter Output Cycles Protein Kaput	disgenet
87	Q99788	CMKLR1	Chemerin Chemokine-Like Receptor 1	disgenet
88	Q8N3K9	CMYA5	Cardiomyopathy Associated 5	disgenet
89	P09543	CNP	2',3'-Cyclic Nucleotide 3' Phosphodiesterase	disgenet
90	P21554	CNR1	Cannabinoid Receptor 1	OMIM
91	P34972	CNR2	Cannabinoid Receptor 2	disgenet
92	P26441	CNTF	Ciliary Neurotrophic Factor	disgenet
93	Q9UHC6	CNTNAP2	Contactin Associated Protein Like 2	disgenet
94	Q96A83	COL26A1	Collagen Alpha-1	pharmGBK
95	P21964	COMT	Catechol O-Methyltransferase	TTD, disgenet, pharmGBK
96	Q7KZN9	COX15	Cytochrome C Oxidase Assembly Protein COX15 Homolog	OMIM
97	P00403	COX2	Cytochrome C Oxidase Subunit 2	disgenet
98	O14810	CPLX1	Complexin 1	disgenet
99	Q6PUV4	CPLX2	Complexin-2	OMIM
100	P16220	CREB1	Cyclic AMP-Responsive Element-Binding Protein 1	disgenet, pharmGBK
101	P06850	CRH	Corticoliberin	disgenet, pharmGBK
102	P24387	CRHBP	Corticotropin-Releasing Factor-Binding Protein	disgenet
103	P34998	CRHR1	Corticotropin-Releasing Factor Receptor 1	TTD, disgenet, pharmGBK
104	Q13324	CRHR2	Corticotropin-Releasing Factor Receptor 2	disgenet, pharmGBK
105	P02741	CRP	C-Reactive Protein	disgenet
106	Q16526	CRY1	Cryptochrome Circadian Regulator 1	disgenet
107	Q49AN0	CRY2	Cryptochrome Circadian Regulator 2	disgenet
108	P09919	CSF3	Colony Stimulating Factor 3	disgenet
109	Q7Z408	CSMD2	CUB And Sushi Multiple Domains 2	disgenet
110	P16410	CTLA4	Cytotoxic T-Lymphocyte Associated Protein 4	disgenet
111	Q9UI47	CTNNA3	Catenin Alpha-3	pharmGBK
112	Q9UQB3	CTNND2	Catenin Delta 2	disgenet
113	P39880	CUX1	Cut Like Homeobox 1	disgenet
114	P02778	CXCL10	C-X-C Motif Chemokine Ligand 10	disgenet
115	P10145	CXCL8	C-X-C Motif Chemokine Ligand 8	disgenet
116	P33261	CYP2C19	Cytochrome P450 Family 2 Subfamily C Member 19	disgenet
117	P11712	CYP2C9	Cytochrome P450 Family 2 Subfamily C Member 9	disgenet
118	P10635	CYP2D6	Cytochrome P450 2D6	disgenet
119	P59103	DAOA	D-Amino Acid Oxidase Activator	disgenet
120	P09172	DBH	Dopamine Beta-Hydroxylase	disgenet, pharmGBK
121	Q8TF63	DCANP1	Dendritic Cell Associated Nuclear Protein	disgenet
122	P20711	DDC	Dopa Decarboxylase	disgenet
123	Q9NX09	DDIT4	DNA Damage Inducible Transcript 4	disgenet
124	O75398	DEAF1	DEAF1, Transcription Factor	disgenet
125	Q9Y6T7	DGKB	Diacylglycerol Kinase Beta	disgenet

126	Q9NRI5	DISC1	Disrupted In Schizophrenia 1 Protein	disgenet, OMIM
127	Q155Q3	DIXDC1	DIX Domain Containing 1	disgenet
128	Q9UBT3	DKK4	Dickkopf WNT Signaling Pathway Inhibitor 4	disgenet
129	Q15700	DLG2	Discs Large MAGUK Scaffold Protein 2	disgenet
130	Q92796	DLG3	Discs Large MAGUK Scaffold Protein 3	disgenet
131	P78352	DLG4	Disks Large Homolog 4	OMIM
132	P27487	DPP4	Dipeptidyl Peptidase 4	disgenet
133	Q16555	DPYSL2	Dihydropyrimidinase Like 2	disgenet
134	P21728	DRD1	D(1A) Dopamine Receptor	disgenet
135	P14416	DRD2	D(2) Dopamine Receptor	disgenet, pharmGBK
136	P35462	DRD3	D(3) Dopamine Receptor	pharmGBK
137	P21917	DRD4	D(4) Dopamine Receptor	disgenet, OMIM
138	P21918	DRD5	Dopamine Receptor D5	disgenet
139	Q96EV8	DTNBP1	Dysbindin	pharmGBK
140	P28562	DUSP1	Dual Specificity Phosphatase 1	disgenet
141	Q13115	DUSP4	Dual Specificity Phosphatase 4	disgenet
142	Q16828	DUSP6	Dual Specificity Protein Phosphatase 6	disgenet
143	Q92611	EDEM1	ER Degradation Enhancing Alpha-Mannosidase Like Protein 1	disgenet
144	P01133	EGF	Epidermal Growth Factor	disgenet
145	P00533	EGFR	Epidermal Growth Factor Receptor	disgenet
146	Q06889	EGR3	Early Growth Response 3	disgenet
147	Q9NZN3	EHD3	EH Domain Containing 3	disgenet
148	P23588	EIF4B	Eukaryotic Translation Initiation Factor 4B	disgenet
149	P54849	EMP1	Epithelial Membrane Protein 1	disgenet
150	Q07075	ENPEP	Glutamyl Aminopeptidase	disgenet
151	P01588	EPO	Erythropoietin	TTD
152	P19235	EPOR	Erythropoietin Receptor	TTD
153	P21860	ERBB3	Erb-B2 Receptor Tyrosine Kinase 3	disgenet
154	Q5RHP9	ERICH3	Glutamate-Rich Protein 3	pharmGBK
155	P03372	ESR1	Estrogen Receptor 1	disgenet
156	Q15485	FCN2	Ficolin-2	pharmGBK
157	Q99581	FEV	FEV, ETS Transcription Factor	disgenet
158	O60258	FGF17	Fibroblast Growth Factor 17	disgenet
159	P09038	FGF2	Fibroblast Growth Factor 2	pharmGBK
160	Q9NP95	FGF20	Fibroblast Growth Factor 20	disgenet
161	P11362	FGFR1	Fibroblast Growth Factor Receptor 1	disgenet
162	P21802	FGFR2	Fibroblast Growth Factor Receptor 2	disgenet
163	P49789	FHIT	Bis	pharmGBK
164	Q02790	FKBP4	FK506 Binding Protein 4	disgenet
165	Q13451	FKBP5	FK506 Binding Protein 5	disgenet, OMIM, pharmGBK
166	Q06787	FMR1	Synaptic Functional Regulator FMR1	OMIM
167	Q04609	FOLH1	Folate Hydrolase 1	disgenet
168	Q9UJU5	FOXD3	Forkhead Box D3	disgenet

169	O15409	FOXP2	Forkhead Box P2	disgenet
170	P0C091	FREM3	FRAS1-Related Extracellular Matrix Protein 3	disgenet
171	P02794	FTH1	Ferritin Heavy Chain 1	disgenet
172	P02792	FTL	Ferritin Light Chain	disgenet
173	Q9C0B1	FTO	FTO, Alpha-Ketoglutarate Dependent Dioxygenase	disgenet
174	O75899	GABBR2	Gamma-Aminobutyric Acid Type B Receptor Subunit 2	disgenet
175	Q06546	GABPA	GA Binding Protein Transcription Factor Subunit Alpha	disgenet
176	P14867	GABRA1	Gamma-Aminobutyric Acid Receptor Subunit Alpha-1	TTD
177	P34903	GABRA3	Gamma-Aminobutyric Acid Type A Receptor Alpha3 Subunit	disgenet
178	Q16445	GABRA6	Gamma-Aminobutyric Acid Receptor Subunit Alpha-6	pharmGBK
179	P47870	GABRB2	Gamma-Aminobutyric Acid Receptor Subunit Beta-2	TTD
180	P28472	GABRB3	Gamma-Aminobutyric Acid Type A Receptor Beta3 Subunit	disgenet
181	O14764	GABRD	Gamma-Aminobutyric Acid Receptor Subunit Delta	TTD
182	P18507	GABRG2	Gamma-Aminobutyric Acid Receptor Subunit Gamma-2	TTD
183	Q99928	GABRG3	Gamma-Aminobutyric Acid Receptor Subunit Gamma-3	TTD
184	O00591	GABRP	Gamma-Aminobutyric Acid Receptor Subunit Pi	pharmGBK
185	Q9UN88	GABRQ	Gamma-Aminobutyric Acid Receptor Subunit Theta	pharmGBK
186	Q99259	GAD1	Glutamate Decarboxylase 1	disgenet
187	Q05329	GAD2	Glutamate Decarboxylase 2	disgenet
188	P22466	GAL	Galanin Peptides [Cleaved Into: Galanin; Galanin Message-Associated Peptide	pharmGBK
189	P47211	GALR1	Galanin Receptor 1	disgenet
190	O43603	GALR2	Galanin Receptor Type 2	disgenet
191	O60755	GALR3	Galanin Receptor 3	disgenet
192	P17677	GAP43	Growth Associated Protein 43	disgenet
193	Q9NZC3	GDE1	Glycerophosphodiester Phosphodiesterase 1	disgenet
194	P39905	GDNF	Glial Cell Line-Derived Neurotrophic Factor	pharmGBK
195	P14136	GFAP	Glial Fibrillary Acidic Protein	OMIM
196	P01241	GH1	Growth Hormone 1	disgenet
197	Q9UBU3	GHRL	Ghrelin And Obestatin Prepropeptide	disgenet
198	P17302	GJA1	Gap Junction Protein Alpha 1	disgenet
199	P23378	GLDC	Glycine Dehydrogenase	pharmGBK
200	Q04760	GLO1	Lactoylglutathione Lyase	disgenet
201	Q68CQ7	GLT8D1	Glycosyltransferase 8 Domain Containing 1	disgenet
202	P15104	GLUL	Glutamate-Ammonia Ligase	disgenet
203	Q9P107	GMIP	GEM Interacting Protein	disgenet
204	P62873	GNB1	G Protein Subunit Beta 1	disgenet
205	P16520	GNB3	G Protein Subunit Beta 3	disgenet
206	P07203	GPX1	Glutathione Peroxidase 1	disgenet
207	P42261	GRIA1	Glutamate Ionotropic Receptor AMPA Type Subunit 1	disgenet
208	P42262	GRIA2	Glutamate Receptor 2	OMIM
209	P42263	GRIA3	Glutamate Receptor 3	pharmGBK

210	Q9ULK0	GRID1	Glutamate Ionotropic Receptor Delta Type Subunit 1	disgenet
211	O43424	GRID2	Glutamate Receptor Ionotropic, Delta-2	OMIM
212	Q13003	GRIK3	Glutamate Ionotropic Receptor Kainate Type Subunit 3	disgenet
213	Q16099	GRIK4	Glutamate Receptor Ionotropic, Kainate 4	pharmGBK
214	Q12879	GRIN2A	Glutamate Receptor Ionotropic, NMDA 2A	OMIM
215	Q13224	GRIN2B	Glutamate Receptor Ionotropic, NMDA 2B	TTDOMIM
216	Q13255	GRM1	Metabotropic Glutamate Receptor 1	OMIM
217	P41594	GRM5	Metabotropic Glutamate Receptor 5	TTD
218	Q14831	GRM7	Glutamate Metabotropic Receptor 7	disgenet
219	P28799	GRN	Granulin Precursor	disgenet
220	P30550	GRPR	Gastrin Releasing Peptide Receptor	disgenet
221	P49840	GSK3A	Glycogen Synthase Kinase 3 Alpha	disgenet
222	P49841	GSK3B	Glycogen Synthase Kinase-3 Beta	disgenet, OMIM, pharmGBK
223	P09488	GSTM1	Glutathione S-Transferase Mu 1	disgenet
224	P30711	GSTT1	Glutathione S-Transferase Theta 1	disgenet
225	P15421	GYPE	Glycophorin E	disgenet
226	O60741	HCN1	Hyperpolarization Activated Cyclic Nucleotide Gated Potassium Channel 1	disgenet
227	O43612	HCRT	Hypocretin Neuropeptide Precursor	disgenet
228	O43613	HCRTR1	Hypocretin Receptor 1	disgenet
229	O43614	HCRTR2	Orexin Receptor Type 2	TTD
230	Q92769	HDAC2	Histone Deacetylase 2	disgenet
231	P56524	HDAC4	Histone Deacetylase 4	disgenet
232	Q9UQL6	HDAC5	Histone Deacetylase 5	disgenet
233	Q9UBN7	HDAC6	Histone Deacetylase 6	disgenet
234	Q9UKV0	HDAC9	Histone Deacetylase 9	disgenet
235	Q16665	HIF1A	Hypoxia Inducible Factor 1 Subunit Alpha	disgenet
236	Q16534	HLF	HLF, PAR Bzip Transcription Factor	disgenet
237	Q86YM7	HOMER1	Homer Scaffold Protein 1	disgenet
238	P00738	HP	Haptoglobin	disgenet
239	Q9Y251	HPSE	Heparanase	disgenet
240	P28845	HSD11B1	Hydroxysteroid 11-Beta Dehydrogenase 1	disgenet
241	P0DMV8	HSPA1A	Heat Shock Protein Family A (Hsp70) Member 1A	disgenet
242	P0DMV9	HSPA1B	Heat Shock Protein Family A (Hsp70) Member 1B	disgenet
243	P34931	HSPA1L	Heat Shock Protein Family A (Hsp70) Member 1 Like	disgenet
244	Q12988	HSPB3	Heat Shock Protein Family B	disgenet
245	P08908	HTR1A	5-Hydroxytryptamine Receptor 1A	TTD, disgenet, pharmGBK
246	P28222	HTR1B	5-Hydroxytryptamine Receptor 1B	TTDOMIM, pharmGBK
247	P28221	HTR1D	5-Hydroxytryptamine Receptor 1D	TTD
248	P28223	HTR2A	5-Hydroxytryptamine Receptor 2A	TTDdisgenet, OMIM, pharmGBK
249	P41595	HTR2B	5-Hydroxytryptamine Receptor 2B	TTD
250	P28335	HTR2C	5-Hydroxytryptamine Receptor 2C	TTD
251	P46098	HTR3A	5-Hydroxytryptamine Receptor 3A	TTD
252	O95264	HTR3B	5-Hydroxytryptamine Receptor 3B	OMIM

253	Q13639	HTR4	5-Hydroxytryptamine Receptor 4	OMIM
254	P34969	HTR7	5-Hydroxytryptamine Receptor 7	TTDdisgenet
255	P42858	HTT	Huntingtin	disgenet
256	P14902	IDO1	Indoleamine 2,3-Dioxygenase 1	TTD
257	P01562	IFNA1	Interferon Alpha 1	disgenet
258	P01563	IFNA2	Interferon Alpha 2	disgenet
259	P01579	IFNG	Interferon Gamma	disgenet
260	P05019	IGF1	Insulin Like Growth Factor 1	disgenet
261	Q14164	IKBKE	Inhibitor Of Nuclear Factor Kappa B Kinase Subunit Epsilon	disgenet
262	P20809	IL11	Interleukin-11	pharmGBK
263	Q14116	IL18	Interleukin 18	disgenet
264	P01583	IL1A	Interleukin 1 Alpha	disgenet
265	P01584	IL1B	Interleukin-1 Beta	disgenet
266	P60568	IL2	Interleukin 2	disgenet
267	Q9NYY1	IL20	Interleukin 20	disgenet
268	Q13007	IL24	Interleukin 24	disgenet
269	P01589	IL2RA	Interleukin 2 Receptor Subunit Alpha	disgenet
270	P05231	IL6	Interleukin-6	TTDdisgenet
271	P08887	IL6R	Interleukin-6 Receptor Subunit Alpha	TTDdisgenet
272	O14732	IMPA2	Inositol Monophosphatase 2	disgenet
273	Q9Y283	INVS	Inversin	pharmGBK
274	Q92985	IRF7	Interferon Regulatory Factor 7	disgenet
275	P19827	ITIH1	Inter-Alpha-Trypsin Inhibitor Heavy Chain 1	disgenet
276	O60229	KALRN	Kalirin Rhogef Kinase	disgenet
277	P78508	KCNJ10	ATP-Sensitive Inward Rectifier Potassium Channel 10	OMIM
278	P48051	KCNJ6	Potassium Voltage-Gated Channel Subfamily J Member 6	disgenet
279	O95069	KCNK2	Potassium Channel Subfamily K Member 2	disgenet
280	P35968	KDR	Kinase Insert Domain Receptor	disgenet
281	O15229	KMO	Kynurenine 3-Monooxygenase	disgenet
282	Q8IZD2	KMT2E	Inactive Histone-Lysine N-Methyltransferase 2E	pharmGBK
283	O00505	KPNA3	Karyopherin Subunit Alpha 3	disgenet
284	Q6YP21	KYAT3	Kynurenine Aminotransferase 3	disgenet
285	P00338	LDHA	Lactate Dehydrogenase A	disgenet
286	P41159	LEP	Leptin	OMIM
287	O95970	LGI1	Leucine-Rich Glioma-Inactivated Protein 1	OMIM
288	Q9H008	LHPP	Phospholysine Phosphohistidine Inorganic Pyrophosphate Phosphatase	disgenet
289	P15018	LIF	LIF, Interleukin 6 Family Cytokine	disgenet
290	Q07954	LRP1	LDL Receptor Related Protein 1	disgenet
291	Q14114	LRP8	LDL Receptor Related Protein 8	disgenet
292	Q13449	LSAMP	Limbic System Associated Membrane Protein	disgenet
293	P09960	LTA4H	Leukotriene A4 Hydrolase	disgenet
294	P20645	M6PR	Mannose-6-Phosphate Receptor, Cation Dependent	disgenet

295	P21397	MAOA	Monoamine Oxidase A	disgenet
296	P27338	MAOB	Monoamine Oxidase B	disgenet
297	P11137	MAP2	Microtubule Associated Protein 2	disgenet
298	Q02750	MAP2K1	Mitogen-Activated Protein Kinase Kinase 1	disgenet
299	Q13163	MAP2K5	Mitogen-Activated Protein Kinase Kinase 5	disgenet
300	P27361	MAPK3	Mitogen-Activated Protein Kinase 3	disgenet
301	P45983	MAPK8	Mitogen-Activated Protein Kinase 8	disgenet
302	Q01726	MC1R	Melanocyte-Stimulating Hormone Receptor	pharmGBK
303	P32245	MC4R	Melanocortin 4 Receptor	disgenet
304	Q99705	MCHR1	Melanin-Concentrating Hormone Receptor 1	OMIM
305	Q6DN12	MCTP2	Multiple C2 And Transmembrane Domain Containing 2	disgenet
306	Q7Z553	MDGA2	MAM Domain-Containing Glycosylphosphatidylinositol Anchor Protein 2	pharmGBK
307	O15151	MDM4	MDM4, P53 Regulator	disgenet
308	Q93074	MED12	Mediator Complex Subunit 12	disgenet
309	Q8WXB1	METTL21A	Protein N-Lysine Methyltransferase METTL21A	pharmGBK
310	Q96C03	MIEF2	Mitochondrial Dynamics Protein MID49	pharmGBK
311	P14174	MIF	Macrophage Migration Inhibitory Factor	disgenet
312	Q96EY8	MMAB	Methylmalonic Aciduria	disgenet
313	P22894	MMP8	Matrix Metallopeptidase 8	disgenet
314	Q86VD1	MORC1	MORC Family CW-Type Zinc Finger 1	disgenet
315	P42898	MTHFR	Methylenetetrahydrofolate Reductase	disgenet, OMIM, pharmGBK
316	P48039	MTNR1A	Melatonin Receptor Type 1A	TTD
317	P42345	MTOR	Mechanistic Target Of Rapamycin Kinase	disgenet
318	Q99707	MTR	5-Methyltetrahydrofolate-Homocysteine Methyltransferase	disgenet
319	Q9UGC7	MTRF1L	Peptide Chain Release Factor 1-Like, Mitochondrial	pharmGBK
320	Q9UL68	MYT1L	Myelin Transcription Factor 1 Like	disgenet
321	Q8NFP9	NBEA	Neurobeachin	pharmGBK
322	P13591	NCAM1	Neural Cell Adhesion Molecule 1	pharmGBK
323	P49821	NDUFV1	NADH:Ubiquinone Oxidoreductase Core Subunit V1	disgenet
324	P19404	NDUFV2	NADH:Ubiquinone Oxidoreductase Core Subunit V2	disgenet
325	Q96PU5	NEDD4L	E3 Ubiquitin-Protein Ligase NEDD4-Like	pharmGBK
326	P07196	NEFL	Neurofilament Light	disgenet
327	P07197	NEFM	Neurofilament Medium	disgenet
328	Q92832	NELL1	Neural EGFL Like 1	disgenet
329	P01138	NGF	Beta-Nerve Growth Factor	disgenet
330	P08138	NGFR	Tumor Necrosis Factor Receptor Superfamily Member 16	OMIM
331	P29475	NOS1	Nitric Oxide Synthase 1	disgenet
332	P35228	NOS2	Nitric Oxide Synthase 2	disgenet
333	P29474	NOS3	Nitric Oxide Synthase 3	disgenet
334	Q9UM47	NOTCH3	Neurogenic Locus Notch Homolog Protein 3	OMIM
335	Q99743	NPAS2	Neuronal PAS Domain Protein 2	disgenet
336	Q8IXF0	NPAS3	Neuronal PAS Domain Protein 3	disgenet

337	P0C0P6	NPS	Neuropeptide S	disgenet
338	Q6W5P4	NPSR1	Neuropeptide S Receptor 1	disgenet
339	P01303	NPY	Pro-Neuropeptide Y	disgenet
340	P25929	NPY1R	Neuropeptide Y Receptor Y1	disgenet
341	P20393	NR1D1	Nuclear Receptor Subfamily 1 Group D Member 1	disgenet
342	P55055	NR1H2	Oxysterols Receptor LXR-Beta	TTD
343	Q13133	NR1H3	Oxysterols Receptor LXR-Alpha	TTD
344	P04150	NR3C1	Glucocorticoid Receptor	TTDdisgenet
345	P08235	NR3C2	Nuclear Receptor Subfamily 3 Group C Member 2	disgenet
346	P43354	NR4A2	Nuclear Receptor Subfamily 4 Group A Member 2	disgenet
347	Q02297	NRG1	Neuregulin 1	disgenet
348	P58400	NRXN1	Neurexin-1-Beta	pharmGBK
349	P20783	NTF3	Neurotrophin 3	disgenet
350	Q9P121	NTM	Neurotrimin	disgenet
351	Q16620	NTRK2	BDNF/NT-3 Growth Factors Receptor	disgenet
352	Q16288	NTRK3	Neurotrophic Receptor Tyrosine Kinase 3	disgenet
353	P30990	NTS	Neurotensin	disgenet
354	P29728	OAS2	2'-5'-Oligoadenylate Synthetase 2	disgenet
355	P41143	OPRD1	Delta-Type Opioid Receptor	TTD
356	P41145	OPRK1	Opioid Receptor Kappa 1	disgenet
357	P41146	OPRL1	Nociceptin Receptor	TTD
358	P35372	OPRM1	Mu-Type Opioid Receptor	OMIM, pharmGBK
359	Q8NG98	OR7D4	Olfactory Receptor Family 7 Subfamily D Member 4	disgenet
360	P32243	OTX2	Homeobox Protein OTX2	OMIM
361	P01178	OXT	Oxytocin/Neurophysin I Prepropeptide	disgenet
362	P30559	OXTR	Oxytocin Receptor	disgenet
363	Q99572	P2RX7	P2X Purinoceptor 7	TTD
364	Q8IVL6	P3H3	Prolyl 3-Hydroxylase 3	pharmGBK
365	Q96RD7	PANX1	Pannexin-1	OMIM
366	O95428	PAPLN	Papilin	pharmGBK
367	Q9NR21	PARP11	Protein Mono-ADP-Ribosyltransferase PARP11	pharmGBK
368	Q96IZ0	PAWR	Pro-Apoptotic WT1 Regulator	disgenet
369	Q9Y6V0	PCLO	Protein Piccolo	disgenet
370	O95613	PCNT	Pericentrin	disgenet
371	Q9HCR9	PDE11A	Phosphodiesterase 11A	disgenet
372	Q01064	PDE1B	Phosphodiesterase 1B	disgenet
373	P27815	PDE4A	Phosphodiesterase 4A	disgenet
374	Q07343	PDE4B	Phosphodiesterase 4B	disgenet
375	Q08499	PDE4D	Camp-Specific 3',5'-Cyclic Phosphodiesterase 4D	disgenet
376	P09619	PDGFRB	Platelet-Derived Growth Factor Receptor Beta	OMIM
377	Q96HC4	PDLIM5	PDZ And LIM Domain 5	disgenet
378	P01213	PDYN	Prodynorphin	disgenet
379	Q15121	PEA15	Proliferation And Apoptosis Adaptor Protein 15	disgenet
380	P01210	PENK	Proenkephalin	disgenet

381	O15055	PER2	Period Circadian Protein Homolog 2	disgenet
382	P56645	PER3	Period Circadian Protein Homolog 3	disgenet, OMIM
383	Q8IYB4	PEX5L	Peroxisomal Biogenesis Factor 5 Like	disgenet
384	Q16875	PFKFB3	6-Phosphofructo-2-Kinase/Fructose-2,6-Biphosphatase 3	disgenet
385	Q9NRD5	PICK1	PRKCA-Binding Protein	OMIM
386	Q9BXM7	PINK1	Serine/Threonine-Protein Kinase PINK1, Mitochondrial	OMIM
387	O75364	PITX3	Paired Like Homeodomain 3	disgenet
388	P14555	PLA2G2A	Phospholipase A2 Group IIA	disgenet
389	Q9NQ66	PLCB1	1-Phosphatidylinositol 4,5-Bisphosphate Phosphodiesterase Beta-1	pharmGBK
390	P00747	PLG	Plasminogen	disgenet
391	P20382	PMCH	Pro-Melanin Concentrating Hormone	disgenet
392	Q13519	PNOC	Prepronociceptin	disgenet
393	P01189	POMC	Pro-Opiomelanocortin	disgenet
394	P27169	PON1	Serum Paraoxonase/Arylesterase 1	disgenet
395	Q15165	PON2	Serum Paraoxonase/Arylesterase 2	pharmGBK
396	Q96A00	PPP1R14A	Protein Phosphatase 1 Regulatory Subunit 14A	OMIM
397	Q9UD71	PPP1R1B	Protein Phosphatase 1 Regulatory Inhibitor Subunit 1B	disgenet
398	P48454	PPP3CC	Protein Phosphatase 3 Catalytic Subunit Gamma	disgenet
399	Q86XR5	PRIMA1	Proline Rich Membrane Anchor 1	disgenet
400	P17252	PRKCA	Protein Kinase C Alpha Type	OMIM
401	P05771	PRKCB	Protein Kinase C Beta	disgenet
402	P05129	PRKCG	Protein Kinase C Gamma Type	OMIM
403	P41743	PRKCI	Protein Kinase C Iota	disgenet
404	Q05513	PRKCZ	Protein Kinase C Zeta Type	OMIM
405	P04156	PRNP	Major Prion Protein	disgenet, OMIM
406	Q9HC23	PROK2	Prokineticin 2	disgenet
407	O43490	PROM1	Prominin 1	disgenet
408	P49768	PSEN1	Presenilin-1	disgenet, OMIM
409	P28070	PSMB4	Proteasome Subunit Beta 4	disgenet
410	P35354	PTGS2	Prostaglandin G/H Synthase 2	disgenet
411	Q15256	PTPRR	Protein Tyrosine Phosphatase, Receptor Type R	disgenet
412	P26022	PTX3	Pentraxin 3	disgenet
413	P10082	PYY	Peptide YY	disgenet
414	Q96PU8	QKI	QKI, KH Domain Containing RNA Binding	disgenet
415	Q15276	RABEP1	Rab Gtpase-Binding Effector Protein 1	pharmGBK
416	P63000	RAC1	Rac Family Small Gtpase 1	disgenet
417	Q92565	RAPGEF5	Rap Guanine Nucleotide Exchange Factor 5	pharmGBK
418	Q70E73	RAPH1	Ras Association	disgenet
419	Q00765	REEP5	Receptor Expression-Enhancing Protein 5	pharmGBK
420	P78509	RELN	Reelin	disgenet
421	P00797	REN	Renin	disgenet
422	Q13127	REST	RE1 Silencing Transcription Factor	disgenet
423	Q969G6	RFK	Riboflavin Kinase	pharmGBK

424	Q08116	RGS1	Regulator Of G Protein Signaling 1	disgenet
425	Q9UGC6	RGS17	Regulator Of G-Protein Signaling 17	pharmGBK
426	Q86UR5	RIMS1	Regulating Synaptic Membrane Exocytosis Protein 1	OMIM
427	Q5XPI4	RNF123	Ring Finger Protein 123	disgenet
428	Q9H4P4	RNF41	Ring Finger Protein 41	disgenet
429	P35398	RORA	Nuclear Receptor ROR-Alpha	pharmGBK
430	P60903	S100A10	Protein S100-A10	disgenet, OMIM
431	P04271	S100B	Protein S100-B	disgenet
432	Q9NTJ5	SACM1L	Phosphatidylinositide Phosphatase SAC1	pharmGBK
433	P21673	SAT1	Spermidine/Spermine N1-Acetyltransferase 1	disgenet
434	P35498	SCN1A	Sodium Channel Protein Type 1 Subunit Alpha	OMIM
435	P53992	SEC24C	SEC24 Homolog C, COPII Coat Complex Component	disgenet
436	P16109	SELP	Selectin P	disgenet
437	P01009	SERPINA1	Alpha-1-Antitrypsin	TTD
438	P08185	SERPINA6	Corticosteroid-Binding Globulin	OMIM
439	P05121	SERPINE1	Plasminogen Activator Inhibitor 1	pharmGBK
440	Q8N474	SFRP1	Secreted Frizzled Related Protein 1	disgenet
441	O43556	SGCE	Epsilon-Sarcoglycan	disgenet, OMIM
442	O00141	SGK1	Serum/Glucocorticoid Regulated Kinase 1	disgenet
443	Q9NRF2	SH2B1	SH2B Adapter Protein 1	pharmGBK
444	Q9H2X9	SLC12A5	Solute Carrier Family 12 Member 5	OMIM
445	Q9P2U8	SLC17A6	Solute Carrier Family 17 Member 6	disgenet
446	P54219	SLC18A1	Solute Carrier Family 18 Member A1	disgenet
447	Q05940	SLC18A2	Solute Carrier Family 18 Member A2	disgenet
448	P43005	SLC1A1	Solute Carrier Family 1 Member 1	disgenet
449	P43004	SLC1A2	Solute Carrier Family 1 Member 2	disgenet
450	P48664	SLC1A6	Solute Carrier Family 1 Member 6	disgenet
451	Q9BZD2	SLC29A3	Solute Carrier Family 29 Member 3	disgenet
452	Q15043	SLC39A14	Zinc Transporter ZIP14	pharmGBK
453	Q9GZV3	SLC5A7	Solute Carrier Family 5 Member 7	disgenet
454	P30531	SLC6A1	Solute Carrier Family 6 Member 1	disgenet
455	Q9NSD5	SLC6A13	Solute Carrier Family 6 Member 13	disgenet
456	Q9H2J7	SLC6A15	Solute Carrier Family 6 Member 15	disgenet
457	P23975	SLC6A2	Sodium-Dependent Noradrenaline Transporter	disgenet
458	Q01959	SLC6A3	Solute Carrier Family 6 Member 3	disgenet
459	P31645	SLC6A4	Sodium-Dependent Serotonin Transporter	TTDdisgenet, OMIM
460	Q9NYB5	SLCO1C1	Solute Carrier Organic Anion Transporter Family Member 1C1	disgenet
461	O75094	SLIT3	Slit Guidance Ligand 3	disgenet
462	P60880	SNAP25	Synaptosomal-Associated Protein 25	disgenet
463	P00441	SOD1	Superoxide Dismutase [Cu-Zn]	disgenet
464	P04179	SOD2	Superoxide Dismutase 2	disgenet
465	Q9UPU3	SORCS3	VPS10 Domain-Containing Receptor Sorcs3	OMIM
466	P48436	SOX9	SRY-Box 9	disgenet

467	P18405	SRD5A1	Steroid 5 Alpha-Reductase 1	disgenet
468	P09132	SRP19	Signal Recognition Particle 19 Kda Protein	pharmGBK
469	Q9BXP5	SRRT	Serrate, RNA Effector Molecule	disgenet
470	P61278	SST	Somatostatin	disgenet
471	P35346	SSTR5	Somatostatin Receptor 5	disgenet
472	P40763	STAT3	Signal Transducer And Activator Of Transcription 3	disgenet
473	P31948	STIP1	Stress Induced Phosphoprotein 1	disgenet
474	P16949	STMN1	Stathmin 1	disgenet
475	P61764	STXBP1	Syntaxin-Binding Protein 1	OMIM
476	P17600	SYN1	Synapsin I	disgenet
477	Q9BQG1	SYT3	Synaptotagmin-3	OMIM
478	O43581	SYT7	Synaptotagmin-7	OMIM
479	Q96RJ0	TAAR1	Trace Amine-Associated Receptor 1	TTD
480	Q96RI8	TAAR6	Trace Amine Associated Receptor 6	disgenet
481	P20366	TAC1	Tachykinin Precursor 1	disgenet
482	P25103	TACR1	Substance-P Receptor	TTD
483	P29371	TACR3	Neuromedin-K Receptor	TTD
484	O43435	TBX1	T-Box Transcription Factor TBX1	OMIM
485	O60806	TBX19	T-Box 19	disgenet
486	Q9UL17	TBX21	T-Box 21	disgenet
487	P48775	TDO2	Tryptophan 2,3-Dioxygenase	disgenet
488	Q10587	TEF	TEF, PAR Bzip Transcription Factor	disgenet
489	O14746	TERT	Telomerase Reverse Transcriptase	disgenet
490	Q9NXF1	TEX10	Testis-Expressed Protein 10	pharmGBK
491	Q12800	TFCP2	Transcription Factor CP2	disgenet
492	P01137	TGFB1	Transforming Growth Factor Beta 1	disgenet
493	P07101	TH	Tyrosine 3-Monooxygenase	disgenet
494	Q9UNS1	TIMELESS	Timeless Circadian Regulator	disgenet
495	Q04724	TLE1	Transducin Like Enhancer Of Split 1	disgenet
496	Q9NYK1	TLR7	Toll Like Receptor 7	disgenet
497	P01375	TNF	Tumor Necrosis Factor	disgenet
498	P19438	TNFRSF1A	TNF Receptor Superfamily Member 1A	disgenet
499	P20333	TNFRSF1B	TNF Receptor Superfamily Member 1B	disgenet
500	O96008	TOMM40	Translocase Of Outer Mitochondrial Membrane 40	disgenet
501	O14656	TOR1A	Torsin-1A	OMIM
502	P17752	TPH1	Tryptophan 5-Hydroxylase 1	disgenet, OMIM
503	Q8IWU9	TPH2	Tryptophan 5-Hydroxylase 2	disgenet, OMIM, pharmGBK
504	Q6UXN2	TREML4	Trem-Like Transcript 4 Protein	pharmGBK
505	P20396	TRH	Thyrotropin Releasing Hormone	disgenet
506	Q9UBN4	TRPC4	Short Transient Receptor Potential Channel 4	TTD
507	Q9UL62	TRPC5	Short Transient Receptor Potential Channel 5	TTD
508	Q8NET8	TRPV3	Transient Receptor Potential Cation Channel Subfamily V Member 3	TTD
509	Q6PGP7	TTC37	Tetratricopeptide Repeat Protein 37	pharmGBK

510	P02766	TTR	Transthyretin	OMIM
511	Q96RR1	TWNK	Twinkle Protein, Mitochondrial	OMIM
512	Q9NNW7	TXNRD2	Thioredoxin Reductase 2, Mitochondrial	pharmGBK
513	P29597	TYK2	Tyrosine Kinase 2	disgenet
514	P19971	TYMP	Thymidine Phosphorylase	TTD
515	P49459	UBE2A	Ubiquitin-Conjugating Enzyme E2 A	OMIM
516	P55089	UCN	Urocortin	disgenet
517	Q969E3	UCN3	Urocortin 3	disgenet
518	P62068	USP46	Ubiquitin Specific Peptidase 46	disgenet
519	Q9Y2C2	UST	Uronyl 2-Sulfotransferase	pharmGBK
520	P63027	VAMP2	Vesicle Associated Membrane Protein 2	disgenet
521	P15692	VEGFA	Vascular Endothelial Growth Factor A	disgenet
522	O15240	VGF	VGF Nerve Growth Factor Inducible	disgenet
523	O76024	WFS1	Wolframin	disgenet
524	Q8IX03	WWC1	WW And C2 Domain Containing 1	disgenet
525	P47989	XDH	Xanthine Dehydrogenase	disgenet
526	P51811	XK	Membrane Transport Protein XK	OMIM
527	Q9H6B1	ZNF385D	Zinc Finger Protein 385D	pharmGBK
